# Pharmacologic treatment of attention deficit hyperactivity disorder in adults: A systematic review and network meta-analysis

**DOI:** 10.1371/journal.pone.0240584

**Published:** 2020-10-21

**Authors:** Jesse Elliott, Amy Johnston, Don Husereau, Shannon E. Kelly, Caroline Eagles, Alice Charach, Shu-Ching Hsieh, Zemin Bai, Alomgir Hossain, Becky Skidmore, Eva Tsakonas, Dagmara Chojecki, Muhammad Mamdani, George A. Wells

**Affiliations:** 1 Cardiovascular Research Methods Centre, University of Ottawa Heart Institute, Ottawa, Ontario, Canada; 2 School of Epidemiology and Public Health, University of Ottawa, Ottawa, Ontario, Canada; 3 Brain and Heart Nexus Research Program, University of Ottawa Heart Institute, Ottawa, Ontario, Canada; 4 Department of Psychiatry, University of Toronto, Toronto, Ontario, Canada; 5 The Hospital for Sick Children, Toronto, Ontario, Canada; 6 Independent Information Specialist, Ottawa, Ontario, Canada; 7 Independent Research Consultant, Montreal, Quebec, Canada; 8 Independent Information Specialist, Edmonton, Alberta, Canada; 9 Li Ka Shing Knowledge Institute, St. Michael's Hospital, Toronto, Ontario, Canada; Copenhagen University Hospital, DENMARK

## Abstract

**Background:**

Attention deficit hyperactivity disorder (ADHD) affects approximately 3% of adults globally. Many pharmacologic treatments options exist, yet the comparative benefits and harms of individual treatments are largely unknown. We performed a systematic review and network meta-analysis to assess the relative effects of individual pharmacologic treatments for adults with ADHD.

**Methods:**

We searched English-language published and grey literature sources for randomized clinical trials (RCTs) involving pharmacologic treatment of ADHD in adults (December 2018). The primary outcome was clinical response; secondary outcomes were quality of life, executive function, driving behaviour, withdrawals due to adverse events, treatment discontinuation, serious adverse events, hospitalization, cardiovascular adverse events, and emergency department visits. Data were pooled via pair-wise meta-analyses and Bayesian network meta-analyses. Risk of bias was assessed by use of Cochrane’s Risk of Bias tool, and the certainty of the evidence was assessed by use of the GRADE framework.

**Results:**

Eighty-one unique trials that reported at least one outcome of interest were included, most of which were at high or unclear risk of at least one important source of bias. Notably, only 5 RCTs were deemed at overall low risk of bias. Included pharmacotherapies were methylphenidate, atomoxetine, dexamfetamine, lisdexamfetamine, guanfacine, bupropion, mixed amphetamine salts, and modafinil. As a class, ADHD pharmacotherapy improved patient- and clinician-reported clinical response compared with placebo (range: 4 to 15 RCTs per outcome); however, these findings were not conserved when the analyses were restricted to studies at low risk of bias, and the certainty of the finding is very low. There were few differences among individual medications, although atomoxetine was associated with improved patient-reported clinical response and quality of life compared with placebo. There was no significant difference in the risk of serious adverse events or treatment discontinuation between ADHD pharmacotherapies and placebo; however, the proportion of participants who withdrew due to adverse events was significantly higher among participants who received any ADHD pharmacotherapy. Few RCTs reported on the occurrence of adverse events over a long treatment duration.

**Conclusions:**

Overall, despite a class effect of improving clinical response relative to placebo, there were few differences among the individual ADHD pharmacotherapies, and most studies were at risk of at least one important source of bias. Furthermore, the certainty of the evidence was very low to low for all outcomes, and there was limited reporting of long-term adverse events. As such, the choice between ADHD pharmacotherapies may depend on individual patient considerations, and future studies should assess the long-term effects of individual pharmacotherapies on patient-important outcomes, including quality of life, in robust blinded RCTs.

**Registration:**

PROSPERO no. CRD 42015026049

## Introduction

Attention deficit hyperactivity disorder (ADHD) is a chronic, multifaceted condition that affects approximately 3% of adults [1] and contributes to important functional impairment and reduced quality of life [2, 3]. Prescriptions for ADHD medication have steadily increased over the last decade [4], leading to important expenditure to public payer health care systems [5]. Practice guidelines recommend a multi-modal approach, including psychosocial interventions and pharmacologic treatments, with continued support and follow-up; the choice between pharmacologic treatments should be based on efficacy and tolerability, as well as duration of effect, affordability, and use of co-medications [2].

Pharmacologic treatment options for ADHD in adults comprise both psychostimulant (e.g., methylphenidate- and amphetamine-based products) and non-stimulant options (e.g., atomoxetine, guanfacine). The availability of individual pharmacotherapies varies by jurisdiction [6], and treatment guidelines similarly differ by region [2, 6]. Canadian guidelines recommend long-acting amphetamine mixture, methylphenidate, or lisdexamfetamine as first-line pharmacologic options, with atomoxetine or short-acting dextroamphetamine or methylphenidate as second-line options for patients whose ADHD does not respond to first-line treatments [7]. British guidelines recommend psychostimulants as first-line treatment for adults, with atomoxetine considered as first-line treatment in some clinical situations (substance use disorder, contraindications to stimulants) [6].

Systematic reviews of randomized clinical trials (RCTs) are generally considered to be the gold standard for decision making in evidence-based medicine. Ideally, RCTs would simultaneously compare all available interventions to treat a given condition; however, in practice, such direct evidence from head-to-head treatment comparisons may be limited or insufficient. Further, pair-wise meta-analyses compare the benefits and harms of two treatments that have been directly compared in head-to-head trials, which may not provide decision-makers (i.e., patients, clinicians, policy makers) with information about the relative benefits and harms of all individual treatments of interest. In contrast, network meta-analyses (NMAs) use direct and indirect comparisons of interventions (i.e., treatments that have not necessarily been studied in head-to-head trials) and provide estimates of the relative effects of each treatment. As such, these types of analyses provide a more robust evidence base for decision-making.

There are multiple available treatment options for ADHD, and few have been directly compared in head-to-head trials. To inform clinical and policy decision-making, we performed a systematic review and NMA of pharmacotherapies available to treat ADHD in adults to assess the relative benefits and harms of each treatment.

## Methods

This review was registered a priori (PROSPERO no. CRD 42015026049) and followed guidance for the conduct and reporting of systematic reviews from the Cochrane handbook [8] and the PRISMA NMA checklist (**S1 File**) [9].

### Search strategy

We updated the search of an earlier evidence synthesis that met the population, intervention, and comparator requirements [10] through an iterative process by an experienced medical information specialist in consultation with the review team. Using the Ovid platform, we searched Ovid MEDLINE®, including In-Process and Other Non-Indexed Citations EMBASE and PsycINFO. We also searched the Cochrane Central Register of Controlled Trials (CENTRAL) using the Wiley version of the Cochrane Library. Grey literature was sought by use of Grey Matters Light [11], which includes the TRIP database, ClinicalTrials.gov and the ICTRP Search Portal The searches were updated from January 2011 onward, except for bupropion, for which there was no date restriction. The initial search was conducted in April 2015 and was updated twice (July 2017, December 2018). The 2008 Cochrane Highly Sensitive Search Strategy, sensitivity and precision-maximizing version, slightly amended, was applied to restrict publications to RCTs. The search strategy is available in **Appendix A in S2 File**.

### Study selection

We included English-language RCTs, including cross-over trials, that included adult (≥ 18 yr) outpatients with attention deficit disorders (attention deficit disorder or ADHD) administered an ADHD pharmacotherapy (**Appendix B in S2 File**). Eligible comparators were placebo (or no treatment), another pharmacotherapy, or the same pharmacotherapy at a different dose. Titles and abstracts identified by the literature search were screened in duplicate, and the full text of any potentially relevant record was evaluated. Records that met the population, intervention, comparator, and design criteria were selected for inclusion; notably, studies were not selected for inclusion on the basis of reported outcomes.

### Data extraction

Data were extracted using DistillerSR software (Evidence Partners Inc.) by one reviewer using piloted standardized abstraction forms; extracted data were checked for accuracy and completeness by a second reviewer. First-period data were extracted from cross-over trials. The primary publication of each unique study was used for data extraction, with additional information obtained from companion publications, supplements, and clinical trial registries, if available.

We extracted information on study design (e.g., first author, year of publication, funding source, country of study), participant characteristics (e.g., age, sex), intervention and comparator details (e.g., type of treatment, dose, duration), and primary and secondary outcomes. If an RCT used more than one scale to assess an outcome (e.g., clinical response), we extracted data from the scale defined by the author as the primary measure or the scale used to inform the sample size calculation. If neither of these were stated, we extracted data from the scale mentioned first in the results section.

### Risk of bias and certainty of the evidence

Risk of bias (ROB) was assessed by two independent reviewers using the Cochrane Collaboration’s ROB tool for RCTs [8]. Disagreements were resolved by discussion. When sufficient data were available, we examined the impact of blinding on subjective outcomes in post-hoc sensitivity analyses involving only studies judged to be at low risk of bias for blinding because of the potential for bias due to patient or investigator expectations. Specifically, studies were deemed to be at low risk of bias for blinding if they involved use of a matched placebo and described that the placebo was indistinguishable from the intervention (e.g., in terms of taste, smell, appearance) or involved a double-dummy placebo. Studies that involved an open-label design or used a method of blinding that was deemed insufficient to prevent knowledge of group assignment were considered to be at high risk of bias. Finally, studies that mentioned blinding but did not provide details about the method of blinding were judged to be at unclear risk of bias. To be considered at overall low risk of bias, trials had to be at low risk of bias for each of the following domains: allocation concealment, blinding, incomplete data.

The risk of publication bias was addressed by conducing comprehensive searches for both published and grey literature and including all eligible studies, regardless of publication status. We also sought additional information from companion publications (e.g., supplements, clinical trial registries, post-hoc analyses), when available, and assessed the source of study funding. Furthermore, we looked for evidence that positive findings were more likely to have been reported in earlier-published studies and visually inspected the funnel plots of outcomes that included data from at least 10 RCTs for obvious signs of asymmetry [8].

The certainty of the evidence for each outcome was assessed by use of the GRADE method [12, 13]. For outcomes based on network meta-analyses, the GRADE assessment was operationalized by use of the CINEMA approach [14].

### Outcomes

The primary outcome was clinical response (patient- or clinician-reported). Secondary outcomes were quality of life, executive function, driving behaviour, withdrawals due to adverse events, treatment discontinuation, serious adverse events, hospitalization, cardiovascular adverse events, and emergency department visits. Eligible scales for clinical response, quality of life, and executive function were based on a previous systematic review [10] (**Appendix C in S2 File**).

### Data analysis and synthesis of results

Before analyses were conducted, included studies with available outcome data were compared to identify obvious sources of between-study heterogeneity. Clinical heterogeneity was assessed by comparing data on study participants, interventions, and comparators; methodological diversity was assessed by comparing study designs and the results of ROB assessments. Based on input from a clinical expert, a decision was made to restrict all analyses of benefits to data reported by RCTs with a treatment duration of at least 12 weeks, whenever possible; an approach that is in keeping with the current clinical recommendations to follow up every three months [2]. Two sets of analyses were conducted for harms; data reported by RCTs with a treatment duration of at least 12 weeks and data reported by RCTs of any treatment duration.

We performed pair-wise meta-analyses (MAs) by use of RevMan (v.5.3; Cochrane Collaboration) and NMAs were performed by use of WinBUGS (v.1.4.3; MRC Biostatistics Unit). All network diagrams were constructed using NodeXL (v.1.0.1.251). Outcomes with insufficient data for analysis are summarized narratively (hospitalizations, visits to the emergency department, cardiovascular events). Evidence suggests that the drug release mechanism and dose of at least some ADHD medications may impact their efficacy in adults [15]. As such, all analyses in this study compared individual ADHD medications, which were further categorized in terms of both drug release formulation (e.g., extended release, immediate release) and dose classification (e.g., low-, standard- or high-dose). Dose classification was based on the Health Canada-approved product monograph, if available, otherwise recommended doses from other jurisdictions were used (**Appendix B in S2 File**).

For MAs, between-study heterogeneity was assessed using the *I*^*2*^ statistic and a visual inspection of forest plots. *I*^*2*^ values of between 75% and 100% were interpreted as possibly representing a considerable amount of heterogeneity [8]; thus, all MAs with *I*^*2*^ values ≥75% were explored to determine if the heterogeneity could be explained by a clinical or methodological feature of one or more studies contributing data to the analysis. In a sensitivity analysis, such studies were removed, and their impact on the summary statistics and *I*^*2*^ value was evaluated [16].

The decision to conduct NMA was based on the sufficiency of data available to derive robust and consistent network models. We used a binomial likelihood model for dichotomous outcomes and a normal likelihood model for continuous outcomes, allowing for the inclusion of multi-arm trials [17]. NMAs were conducted using vague priors (e.g., N[0,100^2^]) assigned for basic parameters throughout [17]. To verify model convergence, trace plots and Brooks-Gelman-Rubin statistics were assessed [18]. Three chains were fit for each analysis with at least 20,000 iterations and a burn-in of at least 20,000 iterations. For all NMAs, we explored both random- and fixed-effects models. When closed loops (i.e., mixed evidence informed by more than one RCT [19]) were present, we assessed inconsistency by comparing deviance information criterion (DIC) statistics in fitted consistency and inconsistency models and examined the resulting inconsistency plot [20].

The choice of model was determined by assessing the goodness of fit statistics, i.e., we compared the mean residual deviance with the number of unconstrained data points (the total number of trials for outcomes analyzed using standardized mean difference (SMDs) and the total number of trial arms for binary data), deviance information criterion (DIC), and between study variance. We considered the most appropriate model to be the one that reported the lowest DIC, and where the total residual deviance was closest to the number of unconstrained data points.

Analyses were based on SMD from baseline as the measurement scale for clinical response, executive function, and quality of life. The total number of participants randomized was used as the denominator for clinical response (number of responders), and the number who received treatment was used for harms (serious adverse events, withdrawals due to adverse events, treatment discontinuations).

Summary measures for continuous outcomes (clinical response, quality of life, executive function) are reported as MDs and 95% confidence intervals (CIs) for pairwise meta-analyses and median MDs and 95% credible intervals (CrIs) for NMAs. SMDs were converted to a common scale by multiplication of the pooled SMD by the pooled standard deviation of baseline scores [8] (patient-reported clinical response: Conners’ Adult ADHD Rating Scale–Self, Short Version [CAARS-S-SV]; clinician-reported clinical response: CAARS–Observer, Short Version [CAARS-O-SV]; executive function: Brief Rating Inventory of Executive Function for Adults [BRIEF-A]; quality of life: Adult ADHD Quality of Life Scale [AAQoL]). Negative MDs indicate improvement in clinical response and executive function, while positive MDs indicate improvement in quality of life. Summary measures for binary data (number of responders, serious adverse events, withdrawals due to adverse events, treatment discontinuations) are reported as relative risks (RRs) with 95% CIs.

## Results

### Study characteristics

**Fig 1:**
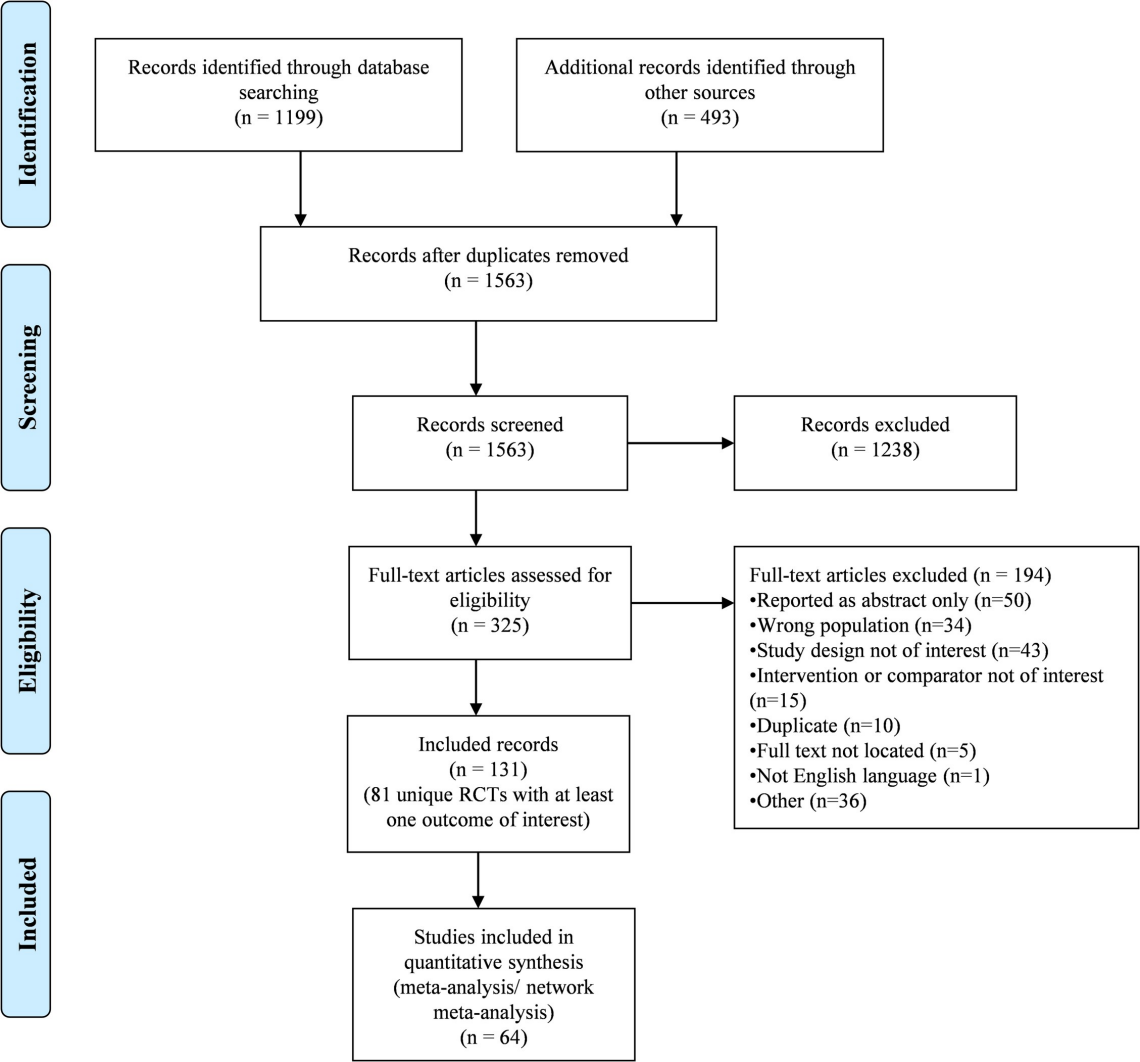
PRISMA flowchart of study selection.

The literature search yielded 1563 unique titles and abstracts (**Fig 1**) of which 131 records met the eligibility criteria (**Appendix D in S2 File)**. These records correspond to a total of 84 unique RCTs, owing to multiple companion records for most RCTs (e.g., one RCT described across multiple publications). Of the 84 unique RCTs, 81 reported at least one outcome of interest (**Table 1**). Two records each reported on two RCTs [21, 22].

**Table 1:** Characteristics of RCTs with at least one reported outcome of interest.

Author year, page no. (companion publications)	Country or region	No. of study sites	Duration	Diagnosis of ADHD	Population	Intervention (no. randomized)	Age, mean (SD)	Male, %	Funding source
**PARALLEL GROUP DESIGN**									
**Paterson 1999, p. 494 [65]**	Australia	NR	6 wk	DSM-IV ADHD symptom checklist	Adults who reported the presence of at least four inattentive and/or five hyperactive symptoms during the previous 6 months (on the DSM-IV ADHD symptom checklist)	Placebo (21)	35.5	60	Non-pharma
						DEX (24)			
**Butterfield 2016, p. 136 [43]**	US	1	12 wk	DSM-IV-TR	Adults with suboptimal response to stimulant ADHD at time of screening	Placebo	37.5 (12.2)	46.2	Pharma
						GUAN 1–4 mg			
						Total: 26			
**Biederman 2012, p. 484 [23] (Biederman 2012)**	US	NR	6 wk	DSM-IV	18–26 yr with childhood-onset ADHD	Placebo (34)	21.6 (2.1)	Total: 62	Pharma
						LIS-DEX 30–70 mg/d (35)			
**Sutherland 2012, p. 445 [74]**	US	8	8 wk	DSM-IV-TR, ACDS v1.2	18–60 yr with ADHD	Placebo (47)	Total:	Total:	Pharma
						ATX 40–100 mg/d (97)	37 (NR)	59	
**Adler 2009, p. 212 [35]**	US	30	12 wk	DSM-IV-TR by CAADID	18–65 yr with ADHD and social anxiety disorder	Placebo (218)	Total:	Total:	Pharma
						ATX 40–100 mg/d (224)	38 (NR)	53.6	
**Weiss 2006, p. 611 [79]**	US, Canada	5	12 wk	DSM-IV	18–66 yr with ADHD	Placebo (26)	NR	64	Pharma
						DEX-IR 10–40 mg/d (23)			
**Biehl 2016, p. 1 [42]**	Germany	1	6 wk	DSM-IV-TR	Adult patients with ADHD	Placebo (16)	35.5 (10.1)	57.1	Mixed
						MPH IR up to 60mg/d (19)	36.7 (10.0)	66.7	
**Schrantee 2016, p. 955 (Bottelier 2014) [26]**	Netherlands	3	16 wk	DSM-IV; confirmed with the Diagnostic Interview for ADHD in Adults	23–40 yr old stimulant treatment-naïve males with ADHD	Placebo (24)	29.0 (4.9)	100	Non-pharma
						MPH (25)	28.6 (4.6)	100	
**Fan 2017, p. 4850 [46]**	Taiwan	1	10 wk	DSM-IV	Adults with ADHD	Placebo (12)	32.5 (9.8)	41.7	Non-pharma
						ATX 1.2 mg/kg (12)	28.9 (7.8)	41.7	
**Goodman 2017, p. 105 [49]**	US	35	6 wk	DSM-IV	18–65 yr with ADHD	Placebo (179)	34.7 (11.60)	54.9	Pharma
						MPH-OROS up to 72 mg/d (178)	36.8 (11.95)	50.9	
**Goto 2017, p. 100 [50]**	Japan, South Korea, Taiwan	45	10 wk	DSM-IV-TR	Asian patients aged 18 years and older with a childhood diagnosis of ADHD	Placebo (196)	31.7 (7.8)	48.7	Pharma
						ATX 80–120 mg/d (195)	32.8 (8.1)	46.6	
**Philipsen 2010, p. 203 [66] (Philipsen 2014, Philipsen 2015)**	Germany	7	1 yr	DSM-IV	18–60 yr with childhood diagnosis of ADHD	Placebo (107)	35 (10)	43.7	Mixed
						MPH-SR up to 60 mg/kg/d (110)	35 (10)	40.5	
**Hamedi 2014, p. 675 [51]**	Iran	1	6 wk	DSM-IV-TR	20–60 yr with ADHD	Placebo (21)	33.19 (4.00)	71.4	Non-pharma
						BUP-SR 75–150 mg/d (21)	33.90 (4.83)	57.1	
**Lee 2014, p. 386 [56]**	Korea, Japan, Taiwan	45	10 wk	DSM-IV-TR, CAADID	Adults with childhood-onset ADHD	Placebo (37)	31.5 (8.6)	35.1	Pharma
						ATX 40–120 mg/d (37)	35.2 (8.8)	41.7	
**Kollins 2014, p. 158 [53]**	US	NR	4 wk	DSM-IV	18–50 yr smokers with ADHD	Placebo (15)	33.5 (9.1)	60.0	Pharma
						LIS-DEX 30–70 mg/d (17)	29.6 (8.4)	64.7	
**Konstenius 2014, p. 440 [28]**	Sweden	1	24 wk	DSM-IV	18–65 yr prison inmates with ADHD	Placebo (27)	42 (11.7)	100	Non-pharma
						MPH-OROS 18–180 mg/d (27)	41 (7.5)	100	
**Takahashi 2014, p. 488 [75]**	Japan	39	8 wk	DSM-IV, CAADID, CAARS-O:SV	18–64 yr with childhood-onset ADHD	Placebo (141)	34.1 (9.05)	48.2	Pharma
						MPH-OROS 18–72 mg/d (143)	33.4 (8.85)	49.7	
**Adler 2013, p. 694 [33] (Adler 2013,Weisler 2017, p.1198)**	US	35	10 wk	DSM-IV-TR	18–85 yr with ADHD	Placebo (81)	34.9 (11.02)	53.8	Pharma
						LIS-DEX 30–70 mg/d (80)	34.2 (10.58)	50.6	
**Durell 2013, p. 45 [25](Leuchter 2014, Adler 2014)**	US	32	12 wk	DSM-IV-TR, ACDS v1.2	18–30 yr with ADHD	Placebo (225)	24.7 (3.5)	56.4	Pharma
						ATX 40–100 mg/d (220)	24.7 (3.4)	58.2	
**Ni 2013, p. 1959 [64] (Ni 2016, Ni 2017)**	Taiwan	1	8–10 wk	DSM-IV, Adult ADHD Self-Report Scale v1.1	18–55 yr with ADHD	MPH-IR 30–60 mg/d (31)	31.4 (7.2)	58.1	Non-pharma
						ATX 0.5–1.2mg/kg/d (32)	31.2 (8.4)	59.4	
**Ginsberg 2012, p. 68 [48] (Ginsberg 2012, p. 705)**	Sweden	1	5 wk	DSM-IV	21–61 yr male prison inmates with ADHD	Placebo (15)	35.3 (NR)	100	Non-pharma
						MPH-OROS 36–72 mg/d (15)	33.5 (NR)	100	
**Retz 2012, p. 48 [68]**	Germany	10	8 wk	DSM-IV ADHD-DC	≥18 yr with childhood-onset ADHD	Placebo (78)	38.2 (9.9)	56	Pharma
						MPH-ER 10–120 mg/d (84)	36.6 (10.4)	38	
**Sobanski 2012, p. 100 [70] (Sobanski 2013)**	Germany	1	12 wk	DSM-IV, CAARS	18–50 yr with ADHD	Placebo (37)	37.4 (9.5)	42.9	Pharma
						ATX 18–80 mg/d (27)	34.7 (8.8)	59.1	
**Young 2011, p. 51 [32] (Wietecha 2012)**	US	42	24 wk	DSM-IV-TR, CAADID	≥18 yr with childhood-onset ADHD	Placebo (234)	41.4 (7.5)	43.6	Pharma
						ATX 40–100 mg/d (268)	41.2 (6.9)	51.1	
**Konstenius 2010, p. 130 [54]**	Sweden	1	12 wk	DSM-IV, CAARS-O:SV	Amphetamine addiction clinic outpatients with newly diagnosed ADHD	Placebo (12)	39.7 (9.8)	83	Non-pharma
						MPH-OROS 18–72 mg/d (12)	34.6 (10.1)	75	
**Biederman 2010, p. 549 [41] (Biederman 2011, Beiderman 2012)**	US	NR	6 wk	DSM-IV, AISRS	19–60 yr with childhood-onset ADHD	Placebo (115)	36.4 (8.6)	52	Pharma
						MPH-OROS 36 to > 100 mg/d (112)	34.7 (9.2)	39	
**McRae-Clark 2010, p. 481 [62]**	US	NR	12 wk	DSM-IV	18–65 yr with ADHD and marijuana dependence	Placebo (39)	30.4 (13.0)	68	Non-pharma
						ATX 25–100 mg/d (39)	29.4 (10.0)	84	
**Winhusen 2010, p. 1680 [82] (Heffner 2013, Westover 2013, Nunes 2013, Berlin 2012, Covey 2011, Covey 2010, Covey 2011, Mamey 2017)**	US	6	11 wk	DSM-IV, ACDS v1.2, ADHD-RS	18–55 yr smokers with ADHD	Placebo (128)	37.5 (9.6)	52.3	Mixed
						MPH-OROS 18–72 mg/d (127)	38.1 (10.4)	60.6	
**Adler 2009, p. 239 [35]**	US	27	7 wk	DSM-IV, AISRS	18–65 yr with childhood-onset ADHD	Placebo (116)	38.2 (11.40)	55.2	Pharma
						MPH-OROS (113)	39.9 (12.27)	57.3	
**Adler 2009, p. 44 [36] (Brown 2011)**	US	21	6 mo	DSM-IV-TR, ACDS v1.2	18–54 yr with ADHD	Placebo (251)	37.4 (9.9)	51.8	Pharma
						ATX 25–100 mg/d (250)	37.7 (10.4)	49.6	
**Rosler 2009, p. 120 [69] (Rosler 2010)**	Germany	28	24 wk	DSM-IV, ADHD RS-IV	≥18 yr with ADHD	Placebo (118)	33.8 (10.6)	50	Pharma
						MPH-ER 10–60 mg/d (241)	35.2 (10.1)	50	
**Adler 2008, p. 720 [34]**	US	22	6 mo	DSM-IV-TR	18–50 yr with childhood-onset ADHD	Placebo (139)	36.0 (8.4)	63.3	Pharma
						ATX 40–100 mg/d (271)	37.1 (8.3)	56.1	
**Spencer 2008, p. 1437 [73] (Spencer 2008)**	US	39	7 wk	DSM-IV-TR, ADHD-RS-IV	18–55 yr with ADHD	Placebo (137)	37.0 (10.3)	49.6	Pharma
						MAS-XR 12.5–75 mg/d (137)	36.1 (10.1)	50.4	
**Wilens 2008, p. 145 [80] (Wilens 2011)**	US, Canada	14	12 wk	DSM-IV-TR, AISRS	≥18yr with ADHD	Placebo (75)	34.8 (9.9)	85.3	Pharma
						ATX 25–100 mg/d (72)	34.3 (10.2)	84.7	
**Levin 2007, p. 20 [58]**	US	2	13 wk	DSM-IV	Adults with ADHD seeking outpatient treatment for cocaine use	Placebo (53)	37 (6)	83	Non-pharma
						MPH-SR 10–60 mg/d (53)	37 (7)	83	
**Biederman 2006, p. 829 [40]**	US	NR	6 wk	DSM-IV	19–60 yr with ADHD	Placebo (77)	37.6 (8.4)	47	Pharma
						MPH-OROS 36 to > 100 mg/d (72)	32.7 (18.5)	57	
**Reimherr 2005, p. 245 [67]**	US	NR	6 wk	DSM-IV, Utah Criteria, WRAADDS	Childhood-onset ADHD	Placebo (24)	34.6 (11.2)	75.0	Pharma
						BUP-SR 100–400 mg/d (35)	34.3 (14.8)	71.4	
**Spencer 2005, p. 456 [71]**	US	NR	6 wk	DSM-IV	19–60 yr with ADHD	Placebo (42)	40.3 (10.0)	54.8	Mixed
						MPH-IR 1.3 mg/kg/d (max) (104)	35.6 (9.7)	59.6	
**Wilens 2005, p. 793 [31]**	US	16	8 wk	DSM-IV	18–60 yr with childhood-onset ADHD	Placebo (81)	41.4 (10.0)	59	Pharma
						BUP-ER 150–450 mg/d (81)	39.1 (10.3)	60	
**Michelson 2003, p. 112 [22] (Faraone 2005)**	US	17	10 wk	DSM-IV by CAADID	Adults with at least moderate ADHD symptoms	Placebo (139)	40.3 (11.6)	62.6	Pharma
						ATX 60–120 mg/d (141)	40.2 (11.7)	64.5	
**Trial 1**									
**Michelson 2003, p. 112 [22] (Faraone 2005)**	US	14	10 wk	DSM-IV by CAADID	Adults with at least moderate ADHD symptoms	Placebo (127)	41.2 (11.2)	68.5	Pharma
						ATX 60–120 mg/d (129)	43.0 (10.3)	64.3	
**Trial 2**									
**Levin 2001, p. 83 [57]**	US	1	4 wk	DSM-IV-TR, CAADID, WRAADDS	19–56 yr with ADHD	Placebo (10)	35.3 (2.1)	50	NR
						MPH-SR 20 mg/d (10)	40.2 (3.6)	80	
**Wilens 2001, p. 282 [81]**	US	1	6 wk	DSM-IV	20–59 yr with ADHD	Placebo (19)	39.6 (10.4)	53	Mixed
						BUP-SR 100–400 mg/d (21)	37.0 (11.8)	57	
**Matochik 1994, p. 658 [61]**	US	2	NR	DSM-III-R, Utah Criteria, CAARS-O:SV	Adults with childhood-onset ADHD	MPH 10–50 mg/d (19)	35.5	68.4	NR
						DEX-IR 10–30 mg/d (18)	35.6	44.4	
**Weisler 2017, p. 685 [77]**	US	43	4 wk	DSM-V	18–55 yr with ADHD and ADHD-RS-AP total score ≥28	Placebo (91)	34.5 (10.8)	47.2	Pharma
						Triple bead MAS 12.5 mg/d (92)	33.0 (10.4)	62.0	
						Triple bead MAS 37.5 mg/d (92)	32.4 (10.0)	56.7	
**Levin 2015, p. 593 [60] (Levin 2018)**	US	2	13 wk	DSM-IV-TR	Adults with co-occurring ADHD and cocaine use disorder	Placebo (43)	39.3 (7.4)	88.4	Non-pharma
						MAS-ER 60 mg (40)	43.9 (7.5)	82.5	
						MAS-ER 80 mg (43)	38.4 (8.6)	81.4	
**Casas 2013, p. 268 [44](Kooij 2013)**	Europe	42	12 wk	DSM-IV-TR by CAADID, CAARS-O:SV	18–65 yr with ADHD	Placebo (97)	35.5 (8.8)	53.6	Pharma
						MPH-OROS 54 mg/d (90)	35.8 (11.7)	48.9	
						MPH-OROS 72 mg/d (92)	35.8 (10.1)	54.3	
**Weisler 2012, p. 421 [78]**	US	37	6 wk	DSM-IV-TR by CAADID, CAARS-S:SV	18–55 yr with ADHD	Placebo (74)	33.4 (10.34)	58.9	Pharma
						MPH-OROS 36–54 mg/d (68)	33.2 (9.73)	66.2	
						ATX 40–80 mg/d (74)	34.6 (10.43)	53.4	
**Levin 2006, p. 137 [59]**	US	5	12 wk	DSM-IV	18–60 yr with ADHD and opiate dependence	Placebo (33)	39 (8)	55	Non-pharma
						MPH-SR 20–80 mg/d (32)	40 (6)	59	
						BUP-SR 100–400 mg/d (33)	38 (8)	66	
**Kuperman 2001, p. 129 [55]**	US	1	7 wk	DSM-IV	Childhood-onset ADHD	Placebo (12)	32.2 (9.8)	73	Pharma
						MPH-IR 0.9 mg/kg/d (max) (12)	31.4 (7.3)	75	
						BUP-SR 300 mg/d (max) (13)	33.2 (10.8)	64	
**Frick 2017, p. 1 [47]**	US	48	6 wk	DSM-IV-TR	18–55 yr	Placebo (104)	35.6 (9.79)	55.8	Pharma
						Triple bead MAS 25 mg (104)	38.0 (9.92)	51.9	
						Triple bead MAS 50 mg (101)	37.2 (9.51)	65.3	
						Triple bead MAS 75 mg (102)	37.9 (9.70)	53.9	
**Huss 2014, p. 44 [52]**	Belgium, Colombia, Denmark, Germany, Norway, Singapore, South Africa, Sweden, US	NR	9wk	DSM-IV, ADHD-RS	18–60 yr with childhood-onset ADHD	Placebo (181)	36.8 (12.15)	55.8	Pharma
						MPH-LAC 40 mg/d (181)	35.1 (11.37)	51.9	
						MPH-LAC 60 mg/d (182)	34.8 (10.79)	57.7	
						MPH-LAC 80 mg/d (181)	34.9 (11.13)	52.5	
**Adler 2008, p. 1364 [34] (Faraone 2012, Mattingly 2013, Babcock 2012, Kollins 2011, Ginsberg 2011, Adler 2009, Adler 2009)**	US	48	4 wk	DSM-IV-TR, ADHD by ADHD-RS-IV-Inv	18–55 yr with ADHD	Placebo (62)	35.2 (10.9)	52	Pharma
						LIS-DEX 30 mg/d (119)	35.3 (10.1)	56	
						LIS-DEX 50 mg/d (117)	34.2 (10.0)	56	
						LIS-DEX 70 mg/d (122)	35.8 (10.5)	52	
**Medori 2008 [63] (Buitelaar 2012, Rosler 2013, Buitelaar 2011)**	Europe	51	5 wk	DSM-IV by CAADID	18–65 yr with childhood-onset ADHD	Placebo (96)	34.5 (NR)	61.5	Pharma
						MPH-OROS 18 mg/d (101)	34.2 (NR)	57.4	
						MPH-OROS 36 mg/d (102)	33.8 (NR)	45.1	
						MPH-OROS 72 mg/d (102)	33.6 (NR)	53.9	
**Weisler 2006, p. 625 [76]**	US	Multisite	4 wk	DSM-IV-TR, CAARS-O:SV	≥ 18yr with ADHD	Placebo (64)	39.3 (NR)	68	Pharma
						MAS-XR 20 mg/d (66)	38.8 (NR)	64	
						MAS-XR 40 mg/d (64)	38.9 (NR)	59	
						MAS-XR 60 mg/d (61)	39.9 (NR)	48	
**Spencer 2007, p. 1380 [72]**	US	18	5 wk	DSM-IV, ADHD-RS	18–60 yr with childhood-onset ADHD	Placebo (53)	38.1 (NR)	50.9	Pharma
						Total DEX-MPH-ER: 168	38.8 (NR)	59.5	
						DEX-MPH-ER 20 mg (58)			
						DEX-MPH-ER 30 mg (55)			
						DEX-MPH-ER 40 mg (55)			
**Arnold 2014, p. 133 [39]**	US	18	9 wk	DSM-IV-TR, ACDS v1.2	Childhood-onset ADHD	Placebo (74)	38.6 (11.15)	53	Pharma
						MODA 255 mg/d (73)	41.1 (10.52)	70	
						MODA 340 mg/d (73)	37.6 (11.49)	58	
						MODA 425 mg/d (74)	39.6 (12.05)	55	
						MODA 510 mg/d (44)	39.6 (12.64)	66	
**CROSS-OVER DESIGN**									
**Herring 2012, p. E891 [88]**	US	6	2 x 4 wk; 1 wk washout	DSM-IV, ACDS v1.2	18–55 yr with childhood-onset ADHD	Placebo (58)	38.4 (11.4)	64.1	Pharma
						MPH-OROS 36–72 mg/d (45)			
						Total: 103			
**Wigal 2018, p. 111 [27]**	US	4	2 x 1 wk	DSM-IV-TR (with ≥6 of 9 subtype criteria met) established by DSM-IV-TR [SCID]	Adults aged 18–55 yrs with baseline ADHD-RS-IV total score ≥24 and an intelligence quotient ≥80 based on the Kaufman Brief Intelligence Test	Placebo	29.1 (10.4)	55.1	Pharma
						Triple bead MAS 25 mg			
						Total: 79			
**Bron 2014, p. 519 [87]**	The Netherlands	1	2 x 2 wk; 1 wk washout	DSM-IV	Drug-naïve adults with childhood-onset ADHD	Placebo	30.5 (7.4)	77.3	Non-pharma
						MPH-OROS 36–72 mg/d			
						Total: 22			
**Martin 2014, p. 147 [91]**	US	1	3 x 1 wk	ACDS v1.2, ADHD-RS-IV	Adults with ADHD and previous successful treatment with an amphetamine-based agent	Placebo	30.8 (10.75)	61.1	Pharma
						LIS-DEX 50 mg/d			
						Total: 18			
**Bain 2013, p. 405 [83]**	US	20	2 x 4 wk; 2 wk washout	DSM-IV-TR, ACDS v1.2, CAARS:Inv	18–60 yr with ADHD	Placebo	36.2 (11.85)	53	Pharma
						ATX 40–80 mg/d			
						Total: 53			
**Wender 2011, p. 36 [98]**	US	NR	2 x 2 wk	Utah Criteria, WRAADDS	21–55 yr with ADHD	Placebo	36.9 (8.5)	72.4	Non-pharma
						MPH-IR 30–60 mg/d			
						Total: 116			
**Wigal 2010, p. 34 [101] (Wigal 2011, Brams 2011)**	US	5	2 x 1 wk	DSM-IV-TR, ACDS v1.2	18–55 yr with ADHD;	Placebo	30.5 (10.7)	62.0	Pharma
						LIS-DEX 30–70 mg/d			
						Total: 127			
**Kay 2009, p. 316 [21]**	US	1	2 x 3 wk	DSM-IV-TR	19–25 yr with ADHD	MAS-XR 20–50 mg/d	22.3 (2.1)	89.5	Pharma
						Placebo			
**Trial 1**						Total: 19			
**Kay 2009, p. 316 [21]**	US	1	2 x 3 wk	DSM-IV-TR	19–25 yr with ADHD	ATX 40–80 mg/d	22.4 (1.8)	87.5	Pharma
						Placebo			
**Trial 2**						Total: 16			
**Verster 2008, p. 230 [97] (Verster 2014)**	The Netherlands	NR	2 x 1 d; 6–7 d washout	DSM-IV	Adults with ADHD	Placebo	38.3 (7.7)	61.1	Non-pharma
						MPH			
						Total: 18			
**Barkley 2007, p. 306 [84]**	US	1	2 x 4 wk	DSM-IV	21–65 yr with childhood-onset ADHD	Placebo	36.1 (12.2)	44	Mixed
						ATX 0.6 to 1.2 mg/kg/d			
						Total: 22			
**Jain 2007, p. 268 [89]**	Canada	Multisite	2 x 2-3wk	DSM-IV	Adults with childhood-onset ADHD	Placebo	37.2 (11.2)	62.5	Pharma
						MPH-MR-STD			
						Total: 50			
**Reimherr 2007, p. 93 [92] (Robison 2010)**	US	NR	2 x 4 wk	DSM-IV-TR. Utah Criteria	18–65 yr adults with ADHD	Placebo	30.6 (10.8)	66	Pharma
						MPH-OROS 18–90 mg/d			
						Total: 47			
**Kooij 2004, p. 973 [90] (Boonstra 2007, Boonstra 2005)**	The Netherlands	1	2 x 3 wk; 1 wk washout	DSM-IV	Adults with childhood-onset ADHD	Placebo (20)	39.1 (20–56)	53.3	Non-pharma
						MPH-IR, 0.5–1 mg/kg/d (25)			
						Total: 45			
**Bouffard 2003, p. 546 [86]**	Canada	NR	2 x 4 wk; 1 wk washout	DSM-IV, CAADID	17–51 yr with childhood onset ADHD	Placebo	34 (NR)	80.0	Non-pharma
						MPH-IR 15–45 mg/d			
						Total: 38			
**Tenenbaum 2002, p. 49 [30]**	US	NR	3 x 3 wk; 2x 1 wk washout	DSM-IV, ADSA	24–53 yr with ADHD	Placebo	42 (NR)	45.8	Pharma
						MPH-IR 10–45 mg/d			
						Total: 33			
**Spencer 2001, p. 775 [93]**	US	NR	2 x 3 wk; 1 wk washout	DSM-IV	19–60 yr with childhood-onset ADHD	Placebo	38.8 (9.27)	56	Mixed
						MAS-XR 20–60 mg/d			
						Total: 30			
**Spencer 1998, p. 693 [94]**	US	NR	2 x 3 wk; 1 wk washout	DSM-III-R	19–60 yr with ADHD	Placebo	34 (9)	47.6	Mixed
						ATX 40–80 mg/d			
						Total: 22			
**Spencer 1995, p. 434 [95]**		US	1	2 x 3 wk; 1 wk washout	DSM-III-R	18–60 yr with childhood-onset ADHD	Placebo	40 (2.1)	43	NR
							MPH-IR up to 1 mg/kg/d			
							Total: 25			
**Wender 1985, p. 547 [99]**		US	1	2 x 2 wk; 1 wk washout	Utah criteria	ADD, residual type	Placebo	31.1 (6.7)	54.1	Non-pharma
							MPH-IR 10–90 mg/d			
							Total: 37			
**Barkley 2005, p. 121 [85]**		US	1	1 x 3 doses; 2 x 7–14 day washout	DSM-IV	18–65 yr with childhood onset ADHD	Placebo	31.3 (11.3)	74	Non-pharma
							MPH-IR- 10 mg			
							MPH-IR- 20 mg			
							Total: 56			
**Taylor 2001, p. 223 [29]**		US	1	3 x 2 wk; 2x 4 d washout	DSM-IV	Adults with childhood onset of ADHD	Placebo	41.2 (11.4)	41	NR
							GUAN-IR 0.25–2 mg/d			
							DEX-IR 2.5–20 mg/d			
							Total: 17			
**Taylor 2000, p. 311 [96]**		US	1	3 x 2 wk; 2x 4 d washout	DSM-IV	≥21 yr with childhood-onset ADHD	Placebo	40.8 (12.5)	59	NR
							MODA 100–400 mg/d			
							DEX-IR 10–40 mg/d			
							Total: 22			
**Wigal 2018, p. 481 [100]**		US	4	3 x 1 wk	DSM-IV-TR	Adults aged 18–55 yrs with baseline ADHD-RS-IV total scores ≥24 and an intelligence quotient ≥80	Placebo	33.1(9.0)	61.6	Pharma
							MAS IR 25 mg			
							Triple bead MAS 50 mg/ Triple bead MAS 75 mg			
							Total: 86			

ADHD = Attention deficit hyperactivity disorder, ADD = attention deficit disorder, ATX = atomoxetine, BUP = bupropion, DEX = dexamphetamine, HD = high dose, ER = extended release, GUAN = guanfacine, LD = low dose, LIS = lisdexamfetamine, MAS = mixed amphetamine salts, MODA = modafinil, MPH = methylphenidate, NR = not reported, OROS = osmotic-release oral system SD = standard deviation, SR = sustained release, STD = standard dose.

In total, 12,423 participants (between 16 and 725 participants randomized per trial) were included in the 81 RCTs that reported an outcome of interest. All included studies required that participants have a clinical diagnosis of ADHD, which was predominantly based on either the 3^rd^, 4^th^, or 5^th^ edition of the *Diagnostic Statistical Manual (DSM) for Mental Disorders* (96%). Participants were both treatment naïve and experienced; most studies required a washout period if ADHD pharmacotherapy had been used before enrollment, although the duration of washout varied by route of administration (**Appendix E in S2 File**). The mean age of participants in the RCTs was generally between 30 and 40 years of age, although six RCTs involved participants with a mean age of less than 30 years [21, 23–27] and six involved participants with a mean age of 41 years and older [22, 28–32].The interventions and comparators were varied among the included RCTs, with 98% involving a placebo control. The interventions considered included methylphenidate (36 RCTs), atomoxetine (20 RCTs), mixed amphetamine salts (9 RCTs), bupropion (6 RCTs), dexamfetamine (6 RCTs), lisdexamfetamine (6 RCTs), guanfacine (2 RCTs), and modafinil (2 RCTs). Most RCTs (70%) involved a parallel-group design [22–24, 26, 28, 31–82], while 30% involved a cross-over design [21, 27, 29, 30, 83–101] (Table **1**). In total, funding by a pharmaceutical company was reported by 57 trials (49 studies fully funded by pharmaceutical industry sources [60%]; 8 with mixed industry and non-industry sources) [21–25, 27, 30–36, 39–45, 47, 49, 50, 52, 53, 55, 56, 63, 66–84, 88, 89, 91–94, 100, 101]. The treatment period of all included studies ranged from one day to 52 weeks; however, approximately 75% of studies involved treatment for less than 12 weeks. Most were conducted in the United States (72%).

### Risk of bias

**Fig 2:**
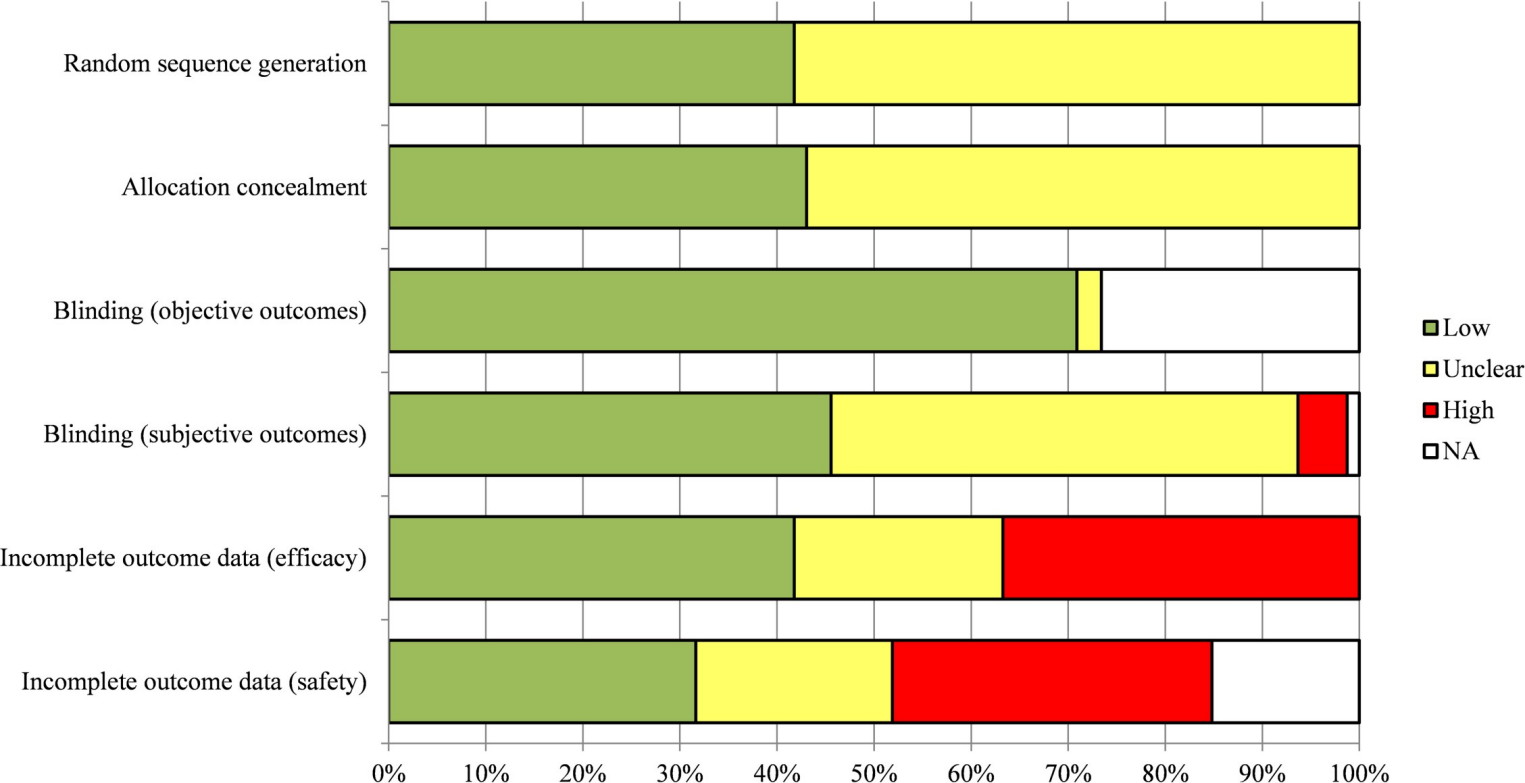
Risk of bias of included randomized controlled trials.

Five RCTs [26, 47–49, 75] were judged to be at low risk of bias across the six domains assessed (**Fig 2**). Less than half (46%) of the included RCTs were at low ROB for blinding, which is an important consideration for subjective outcomes. Fifty-eight percent of studies were judged to be at unclear or high ROB because of incomplete data pertaining to benefit outcomes (e.g., studies were missing outcome data, authors performed as-treated analyses, or incompletely reported discontinuations across study groups). Similarly, just over half of trials (53%) were judged to be at unclear or high ROB because of incomplete harms data. Overall, most studies (91%) were at high or unclear risk of at least one important risk of bias (allocation concealment, blinding, incomplete data). A detailed summary of all ROB judgements across all domains is located in **Appendix F in S2 File**.

Publication bias was not detected for outcomes with at least 10 included RCTs (clinical response, executive function, treatment discontinuation, withdrawal due to adverse events, and serious adverse events) based on visual inspections of funnel plots for the presence of a skewed or asymmetric shape (**Appendix G in S2 File**). Furthermore, we did not identify clear evidence that earlier-published studies were more likely to have published positive results compared to studies published more recently.

### Synthesis of results

Both NMAs and MAs were performed for both patient- and clinician-reported clinical response using data reported by studies with a treatment duration of 12 weeks or longer. These analyses were based on clinical response as measured on a continuous scale and as a dichotomous measure (treatment response yes versus no). MAs were performed for quality of life (≥ 12-week data only), executive function (all treatment durations), and all harms outcomes (**Table 2**). A summary of findings is presented in **Table 3.**

**Table 2:** Summary of analyses completed.

Outcome	Treatment durations analyzed	Type of measure	No. of trials*	No. of participants	Duration (range, wk)
**BENEFITS**^£^					
**Patient-reported clinical response**	≥12 wk	Continuous	9	1462	12–52
		Dichotomous	4	322	12–24
**Clinician-reported clinical response**	≥12 wk	Continuous	15	3365	12–52
		Dichotomous	8	1902	12–24
**Quality of Life†**	≥12 wk	Continuous	5^‡^	1862	12–26
**Executive Function†**	Any duration**	Continuous	11	3024	2–10
**HARMS**					
**Serious adverse events†**	≥12 wk	Dichotomous	5	1380	12–24
	Any duration	Dichotomous	33	7161	1–24
**Withdrawals due to adverse events†**	≥12 wk	Dichotomous	16	3650	12–52
	Any duration	Dichotomous	52	10726	2–52
**Treatment discontinuation†**	≥12 wk	Dichotomous	16	3568	12–26
	Any duration	Dichotomous	51	9959	3–26

* Data are for trials included in analyses. Two included publications each reported data from two unique trials, thus, the number of trials is greater than the number of included publications.

^**£**^ Patient and clinician-reported clinical response were analyzed by both pairwise meta-analysis and Bayesian network meta-analysis; quality of life and executive function were analyzed by pairwise meta-analysis only.

†Analyzed by pair-wise meta-analysis.

‡All studies involved atomoxetine.

**No studies with a treatment duration of at least than 12 weeks assessed executive function.

**Table 3:** Summary of findings.

**A. PATIENT-REPORTED CLINICAL RESPONSE (Continuous; Conner's ADULT ADHD Rating Scales—Self-report, short version)**						
**Intervention options (9 RCTs; 1462 participants in total**)	**Relative effect (95% CI)**		**№ of participants**		**Certainty of the evidence**	**Comments**
	**(network estimates)**		**(studies)**		**(GRADE)**	
**ATX-STD**	**MD: -5.9 (-12.6, -0.4)**		388		⨁◯◯◯	Downgraded because of within-study bias, indirectness, incoherence
			(4 RCTs)		VERY LOW	
**BUP-SR-STD**	MD: -0.8 (-13.5, 11.4)		33		⨁◯◯◯	Downgraded because of within-study bias, indirectness, imprecision, incoherence
			(1 RCT)		VERY LOW	
**MPH-OROS-STD**	MD: -4.7 (-14.1, 3.7)		194	⨁◯◯◯	Downgraded because of within-study bias, indirectness, incoherence
			(2 RCTs)			VERY LOW
**MPH-OROS-HD**	MD: -6.3 (-19.2, 6.4)		27		⨁◯◯◯	Downgraded because of within-study bias, indirectness, incoherence
			(1 RCT)		VERY LOW	
**MPH-SR-STD**	MD: -4.8 (-16.5, 7.1)		110		⨁◯◯◯	Downgraded because of within-study bias, indirectness, incoherence
			(1 RCT)		VERY LOW	
**MPH-SR-HD**	MD: 3.3 (-9.3, 15.6)		32		⨁◯◯◯	Downgraded because of within-study bias, indirectness, incoherence
			(1 RCT)		VERY LOW	
**PLACEBO**	Reference comparator		NA		NA	Network reference comparator
**B. PATIENT-REPORTED CLINICAL RESPONSE (Dichotomous)**						
**Intervention options (4 RCTs; 322 participants in total**)	Anticipated absolute effects* **(95% CI)**		**Relative effect; (95% CI)**	**№ of participants**	**Certainty of the evidence**	**Comments**
	**Without intervention†**	**Risk with ADHD pharmacotherapies**	**(network estimates)**	**(studies)**	**(GRADE)**	
**ATX-STD**	347 per 1,000	1000 per 1,000	**RR 3.34**	27	⨁◯◯◯	Downgraded because of within-study bias, indirectness, heterogeneity, incoherence
		(652 to 1,000)	**(1.88, 5.85)**	(1 RCT)	VERY LOW	
**BUP-SR-STD**	347 per 1,000	378 per 1,000	RR 1.09	33	⨁◯◯◯	Downgraded because of within-study bias, indirectness, imprecision, incoherence
		(139 to 811)	(0.40,2.34)	(1 RCT)	VERY LOW	
**MPH-OROS-HD**	347 per 1,000	832 per 1,000	**RR 2.40**	27	⨁◯◯◯	Downgraded because of within-study bias, indirectness, heterogeneity, incoherence
		(361 to 1,000)	**(1.10,4.06)**	(1 RCT)	VERY LOW	
**MPH-SR-STD**	347 per 1,000	277 per 1,000	RR 0.80	53	⨁◯◯◯	Downgraded because of within-study bias, indirectness, imprecision, incoherence
		(104 to 600)	(0.30,1.73)	(1 RCT)	VERY LOW	
**MPH-SR-HD**	347 per 1,000	239 per 1,000	RR 0.69	32	⨁◯◯◯	Downgraded because of within-study bias, indirectness, imprecision, incoherence
		(80 to 600)	(0.23,1.73)	(1 RCT)	VERY LOW	
**PLACEBO**	Reference comparator	NA	NA	NA	NA	Network reference comparator
**C. CLINICIAN-REPORTED CLINICAL RESPONSE (Continuous)**						
**Intervention options (15 RCTs; 3365 participants in total**)	**Relative effect (95% CI)**		**№ of participants**		**Certainty of the evidence**	**Comments**
	**(network estimates)**		**(studies)**		**(GRADE)**	
**MAS-XR-HD**	MD: -4.2 (-12.1, 3.5)		83		⨁◯◯◯	Downgraded because of within-study bias, indirectness, incoherence
			(1 RCT)		VERY LOW	
**ATX-STD**	MD: **-3.7 (-6.7, -0.9)**		1100		⨁◯◯◯	Downgraded because of within-study bias, indirectness, incoherence
			(7 RCTs)		VERY LOW	
**GUAN-STD**	MD: -0.6 (-9.4, 8.3)		13		⨁◯◯◯	Downgraded because of within-study bias, indirectness, imprecision, incoherence
			(1 RCTs)		VERY LOW	
**MPH-OROS-STD**	MD: -1.4 (-7.0, 4.4)		194		⨁◯◯◯	Downgraded because of within-study bias, indirectness, incoherence
			(2 RCTs)		VERY LOW	
**MPH-ER-STD**	MD: -3.9 (-11.5, 3.7)		241		⨁◯◯◯	Downgraded because of within-study bias, indirectness, incoherence
			(1 RCT)		VERY LOW	
**MPH-SR-STD**	**MD: -5.7 (-11.2, -0.3)**		163		⨁◯◯◯	Downgraded because of within-study bias, indirectness, incoherence
			(2 RCTs)		VERY LOW	
**MPH-LD**	**MD: -10.4 (-19.0, -2.1)**		25		⨁◯◯◯	Downgraded because of within-study bias, indirectness, incoherence
			(1 RCTs)		VERY LOW	
**PLACEBO**	Reference comparator		NA		NA	Network reference comparator
**D. CLINICIAN-REPORTED CLINICAL RESPONSE (Dichotomous)**						
**Intervention options (8 RCTs; 1902 participants in total**)	Anticipated absolute effects* **(95% CI)**		**Relative effect (95% CI)**	**№ of participants**	**Certainty of the evidence**	**Comments**
	**Without intervention†**	**Risk with ADHD pharmacotherapies**	**(network estimates)**	**(studies)**	**(GRADE)**	
**ATX-STD**	298 per 1,000	594 per 1,000	RR 1.99	1336	⨁◯◯◯	Downgraded because of within-study bias, indirectness, incoherence
		(286 to 1,000)	(0.96 to 4.14)	(3 RCTs)	VERY LOW	
**BUP-SR-STD**	298 per 1,000	215 per 1,000	RR 0.72	854	⨁◯◯◯	Downgraded because of within-study bias, indirectness, imprecision, incoherence
		(18 to 976)	(0.06 to 3.27)	(1 RCT)	VERY LOW	
**MPH-OROS-STD**	298 per 1,000	451 per 1,000	RR 1.51	1003	⨁◯◯◯	Downgraded because of within-study bias, indirectness, imprecision, incoherence
		(54 to 1,000)	(0.18 to 3.95)	(1 RCT)	VERY LOW	
**MPH-ER-STD**	298 per 1,000	471 per 1,000	RR 1.58	1062	⨁◯◯◯	Downgraded because of within-study bias, indirectness, imprecision, incoherence
		(125 to 889)	(0.42 to 2.98)	(1 RCT)	VERY LOW	
**MPH-SR-STD**	298 per 1,000	340 per 1,000	RR 1.14	874	⨁◯◯◯	Downgraded because of within-study bias, indirectness, imprecision, incoherence
		(33 to 1,000)	(0.11 to 3.78)	(1 RCT)	VERY LOW	
**MPH-SR-HD**	298 per 1,000	122 per 1,000	RR 0.41	853	⨁◯◯◯	Downgraded because of within-study bias, indirectness, imprecision, incoherence
		(9 to 806)	(0.03 to 2.70)	(1 RCT)	VERY LOW	
**MPH-LD**	298 per 1,000	1000 per 1,000	RR 3.57	846	⨁◯◯◯	Downgraded because of within-study bias, indirectness, heterogeneity, incoherence
		(337 to 1,000)	(1.13 to 5.44)	(1 RCT)	VERY LOW	
**PLACEBO**	Reference comparator	NA	NA	NA	NA	Network reference comparator

*Full GRADE assessment for all outcome is available in Appendix H in S2 File.

#### Benefits

*Patient-reported clinical response*. Among RCTs with a treatment duration of at least 12 weeks, nine trials (n = 1462) [28, 36, 44, 54, 59, 62, 66, 70, 80] assessed patient-reported clinical response by use of a continuous scale, and four trials (n = 322) [28, 58, 59, 70] assessed the outcome as a dichotomous measure (treatment response yes versus no) (**Table 2**). Only one [59] of the four trials contributing data to the analysis of patient-reported clinical response as a dichotomous measure was judged to be of low ROB for blinding compared to three of nine (33%) trials [44, 59, 62] contributing data to the analysis of continuous data for this outcome. None of the studies that reported this outcome were at low risk of bias across all domains. Additional information is contained within **Appendix I in S2 File**.

**Fig 3:**
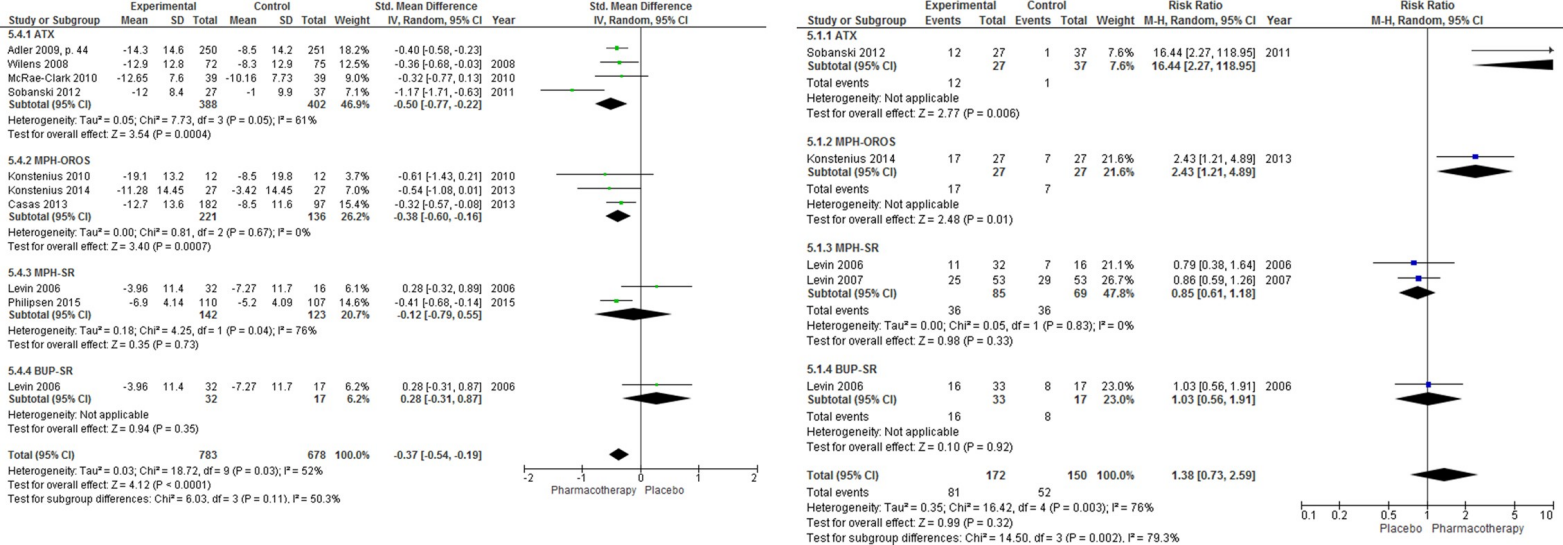
Patient-reported clinical response. Clinical response among patients who received any ADHD pharmacotherapy (versus placebo) in trials with a treatment duration of at least 12 weeks. (A) Continuous measure of response. (B) Dichotomous (treatment response: yes versus no).

The results of pairwise MA showed that, compared with placebo, treatment with any ADHD pharmacotherapy was associated with a small but statistically significant improvement in patient-reported clinical response (MD –4.34, 95% CI –6.34 to –2.23) (**Fig 3A**); with no statistically significant difference in the number of clinical responders (RR 1.38, 95% CI 0.73 to 2.59; **Fig 3B**). Moderate to high heterogeneity was noted for both continuous and dichotomous measures of clinical response (*I*^2^ = 52% and 76%, respectively).

**Fig 4:**
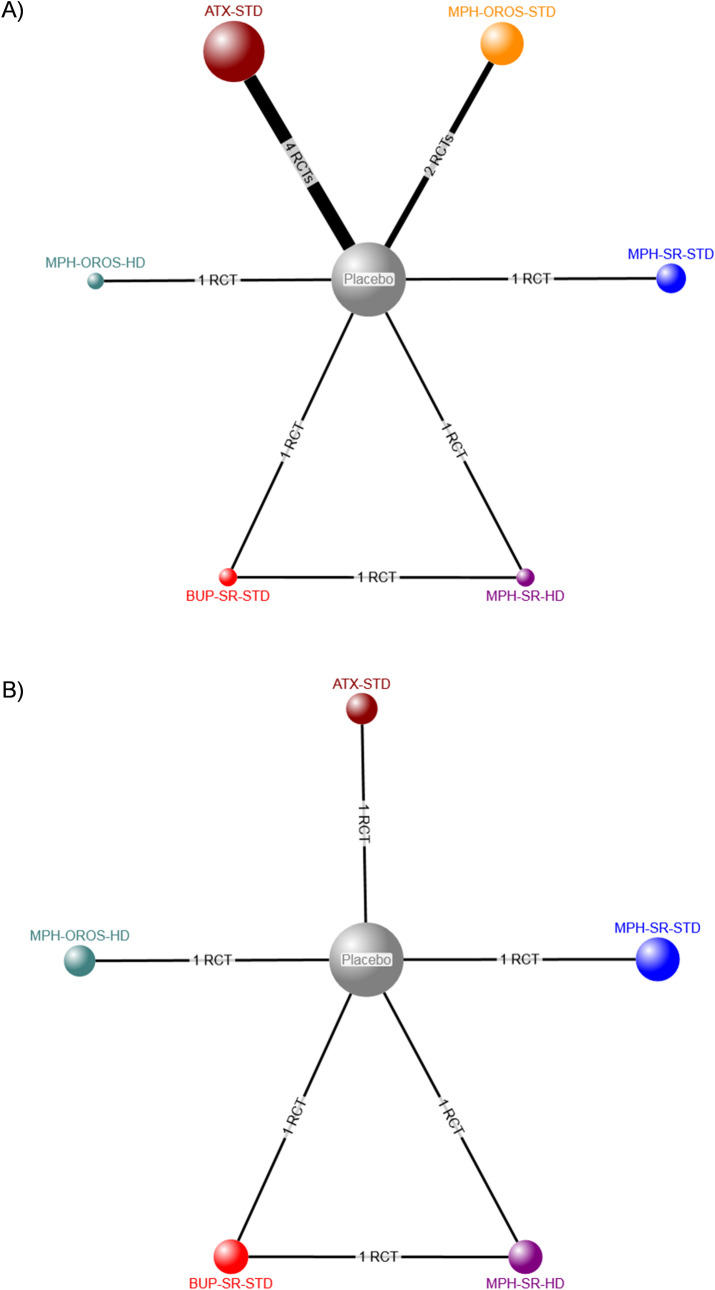
Network diagrams for patient-reported clinical response. Treatment nodes are proportional to the number of patients who took the corresponding treatment, while the width of each edge is proportional to the number of trials included in the comparison. Intervention abbreviations: ATX = atomoxetine, BUP-SR = sustained release bupropion, HD = high dose, MPH = methylphenidate, OROS = osmotic-release oral system, STD = standard dose, SMD = standardized mean difference, SR = sustained release. (A) Continuous measure of response. (B) Dichotomous (treatment response: yes versus no).

The evidence network for patient-reported clinical response as measured by a continuous scale included nine RCTs (eight two-arm, one three-arm) [28, 36, 44, 54, 59, 62, 70, 80, 102] and 1462 participants randomized to six pharmacotherapies, placebo, or no treatment (**Fig 4A**), while the network for clinical response assessed as a dichotomous variable included four RCTs (three two-arm, one three-arm) [28, 58, 59, 70] and involved 322 participants (**Fig 4B**). Consistency could not be formally evaluated for either NMA because of a lack of closed loops informed by more than one RCT [19].

When clinical response to individual ADHD pharmacotherapies was assessed as a continuous measure, only standard-dose atomoxetine resulted in moderate significant improvements versus placebo (MD –5.9, 95% CrI –12.6 to –0.4). There were no significant differences in patient-reported continuous response between any of the ADHD pharmacotherapies (**Table 4A**). When assessed as a dichotomous outcome, clinical response with standard-dose atomoxetine and high-dose osmotic-release oral-system methylphenidate were significantly better than placebo at improving the number of patients who reported a clinical response (RR 3.34, 95% CrI 1.88 to 5.85 and RR 2.40, 95% CrI 1.10 to 4.06, respectively).

**Table 4:** Patient-reported clinical response in trials with a treatment duration of at least 12 weeks–indirect comparison of ADHD pharmacotherapies.

**A) CONTINUOUS measure of response**							
	Mean difference (95% credible interval)*						
	**Placebo**	**ATX-STD**	**BUP-SR-STD**	**MPH-OROS-STD**	**MPH-OROS-HD**	**MPH-SR-STD**	**MPH-SR- HD**
**Placebo**	—						
**ATX-STD**	**-5.9**	—					
	**(-12.6, -0.4)**						
**BUP-SR-STD**	-0.8	5.2	—				
	(-13.5, 11.4)	(-8.3, 19.3)					
**MPH-OROS-STD**	-4.7	1.2	-3.9	—			
	(-14.1, 3.7)	(-9.6, 11.9)	(-19.4, 11.3)				
**MPH-OROS-HD**	-6.3	-0.4	-5.6	-1.6	—		
	(-19.2, 6.4)	(-14.0, 14.3)	(-23.5, 12.4)	(-17.0, 14.1)			
**MPH-SR-STD**	-4.8	1.2	-4	0	1.6	—	
	(-16.5, 7.1)	(-11.5, 15.1)	(-20.7, 13.5)	(-14.4, 15.3)	(-15.4, 19.3)		
**MPH-SR-HD**	3.3	9.3	4.1	8.1	9.7	8.1	—
	(-9.3, 15.6)	(-4.0, 23.6)	(-8.1, 16.6)	(-6.6, 23.7)	(-8.4, 27.5)	(-9.2, 25.1)	
Note: ATX = atomoxetine, BUP-SR = sustained release bupropion, HD = high dose, MPH = methylphenidate, OROS = osmotic-release oral system, STD = standard dose, SR = sustained release.							
*Random-effects model. A negative value indicates improvement in clinical response. Pooled mean differences expressed on the Conners’ ADULT ADHD Rating Scale-Self (CAARS-S), short form. Statistically significant changes are indicated by use of bold and colour (green indicates that the row treatment is significantly better than the column treatment). White indicates no significant difference between treatments.							
**B) DICHOTOMOUS (treatment response yes versus no)**							
	Relative risk (95% credible interval)*						
	**Placebo**	**ATX-STD**	**BUP-SR-STD**	**MPH-OROS-HD**	**MPH-SR-STD**	**MPH-SR-HD**	
**Placebo**	—						
**ATX-STD**	**3.34**	—					
	**(1.88, 5.85)**						
**BUP-SR-STD**	1.09	**0.33**	—				
	(0.40,2.34)	**(0.11,0.78)**					
**MPH-OROS-HD**	**2.4**	0.73	2.21	—			
	**(1.10,4.06)**	(0.32,1.26)	(0.77,6.41)				
**MPH-SR-STD**	0.8	**0.24**	0.74	**0.34**	—		
	(0.30,1.73)	**(0.08,0.60)**	(0.22,2.59)	**(0.11,0.98)**			
**MPH-SR-HD**	0.69	**0.21**	0.65	**0.29**	0.87	—	
	(0.23,1.73)	**(0.06,0.59)**	(0.23,1.73)	**(0.09,0.94)**	(0.22,3.33)		

Note: ATX = atomoxetine, BUP-SR = sustained release bupropion, HD = high dose, MPH = methylphenidate, OROS = osmotic-release oral system, STD = standard dose, SR = sustained release.

*Random-effects model. A relative risk greater than one (green) indicates that a statistically significantly higher proportion of participants in the row treatment achieved treatment response relative to the column treatment (e.g., significantly more participants who received ATX-STD achieved treatment response compared with placebo). A relative risk lower than one (red) indicates that a statistically significantly higher proportion of participants in the column treatment achieved treatment response relative to the row treatment (e.g., significantly more participants who received ATX-STD achieved treatment response compared with BUP-SR-STD). White indicates no statistically significant difference between treatments.

Among the other treatments, use of standard-dose sustained-release bupropion resulted in significantly fewer responders compared with standard-dose atomoxetine (RR 0.33, 95% CrI 0.11, 0.78). High- and standard-dose sustained-release methylphenidate also resulted in significantly fewer responders compared to standard-dose atomoxetine (RR 0.21 and 0.24, respectively). High-dose osmotic-release oral system methylphenidate was significantly better than both standard- and high-dose sustained-release methylphenidate at improving the number of clinical responders (**Table 4B**).

*Clinician-reported clinical response*. Among RCTs with a treatment duration of at least 12 weeks, clinical response was assessed by a clinician as a continuous measure in 15 RCTs (n = 3365) [25, 26, 32, 35, 36, 43, 44, 54, 58, 60, 62, 66, 69, 70, 80] and as a dichotomous measure in eight RCTs (n = 1902) [25, 26, 32, 44, 58, 59, 69, 70] (**Table 2**). Only half [25, 26, 44, 59] of the trials contributing data to the analysis of clinician- reported clinical response as a dichotomous measure were judged to be at low ROB for blinding compared to 40% of studies contributing data to the analysis of continuous data for this outcome. Further, only one study [26] was at low risk of bias across all ROB domains, reporting a significant improvement in clinician-reported clinical response among men (n = 25) who received 16 weeks of methylphenidate treatment but not among those who received placebo (n = 24).

**Fig 5:**
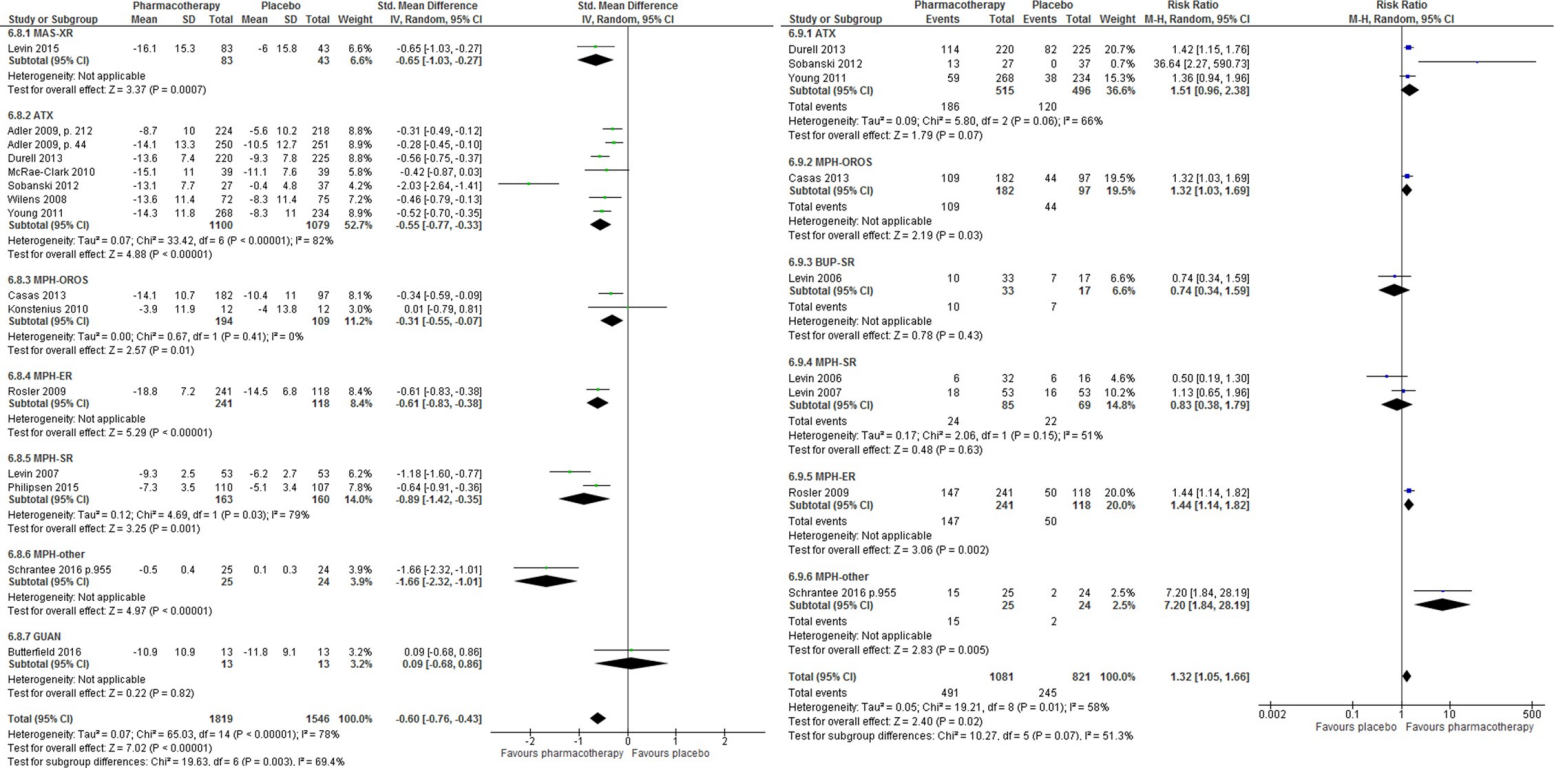
Clinician-reported clinical response. Clinical response among patients who received any ADHD pharmacotherapy (versus placebo) in trials with a treatment duration of at least 12 weeks. (A) Continuous measure of response. (B) Dichotomous (treatment response: yes versus no).

The results of pairwise MA showed that, compared with placebo, the number of patients who showed a clinical response was significantly higher among those using any ADHD pharmacotherapy (RR 1.32, 95% CI 1.05 to 1.66, *I*^2^ = 58%). Further, the use of any ADHD pharmacotherapy revealed a significant moderate improvement in clinical response compared to placebo on the CAARS-O-SV scale (MD –3.89, 95%CI –4.49 to –2.76), although the *I*^2^ value associated with this analysis was 78%, which indicates a considerable amount of heterogeneity between studies (**Fig 5**).

**Fig 6:**
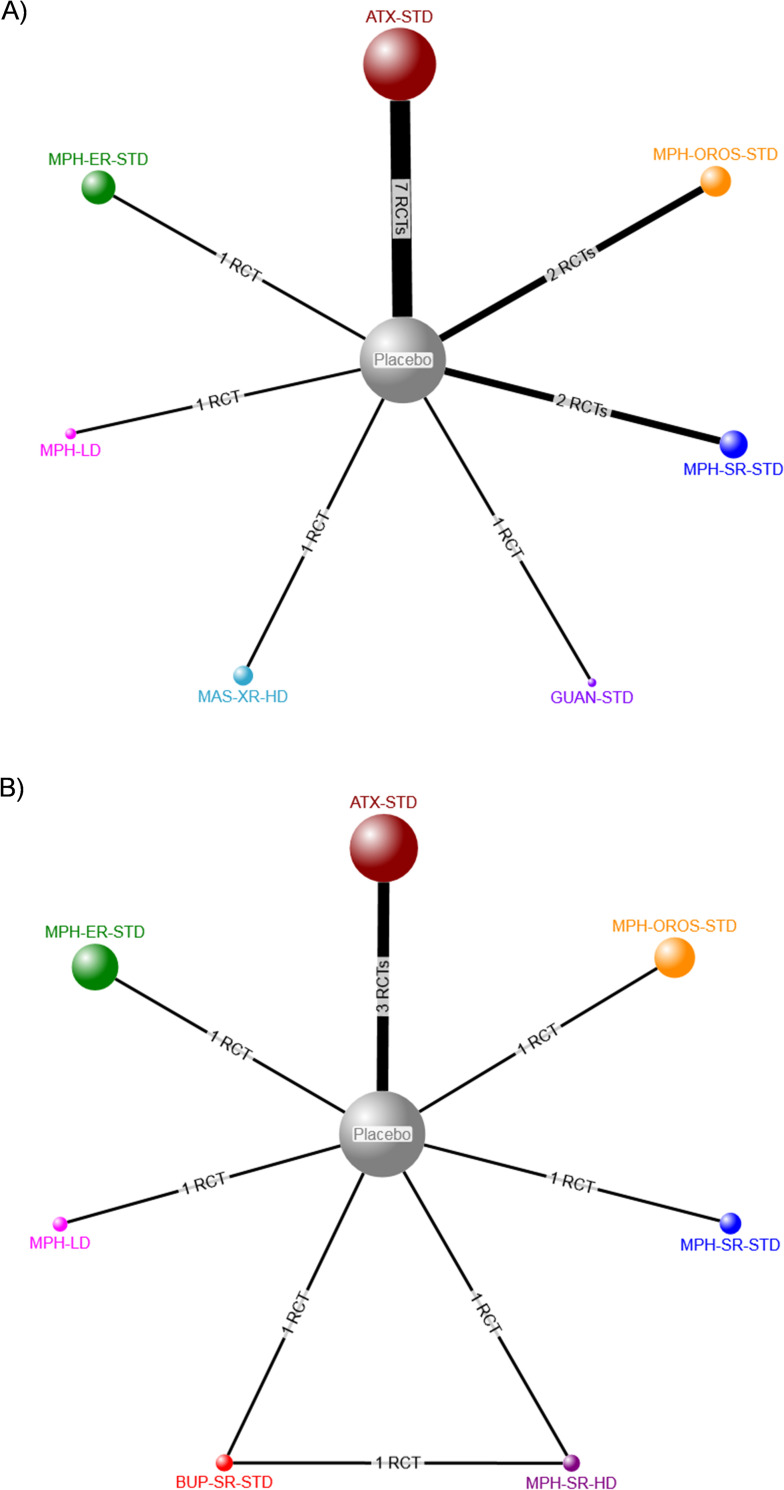
Network diagrams for patient-reported clinical response. Treatment nodes are proportional to the number of patients who took the corresponding treatment, while the width of each edge is proportional to the number of trials included in the comparison. Intervention abbreviations: ATX = atomoxetine, GUAN = guanfacine, HD = high dose, MAS-XR = mixed amphetamine salts, MPH = methylphenidate, OROS = osmotic-release oral system, ER = extended release, SR = sustained release, STD = standard dose. (A) Continuous measure of response. (B) Dichotomous (treatment response: yes versus no).

The evidence network for the effect of individual ADHD pharmacotherapies on clinician-reported clinical response (continuous) included 15 RCTs (15 two-arm trials) [25, 26, 32, 35, 36, 43, 44, 54, 58, 60, 62, 66, 69, 70, 80] involving 3365 participants who were randomized to seven pharmacotherapies, placebo, or no treatment (**Fig 6A**). The network for the number of responders (dichotomous) included 1902 participants randomized to seven pharmacotherapies, placebo, or no treatment in eight RCTs (seven 2-arm, one 3-arm) (**Fig 6B**) [25, 32, 44, 58, 59, 69, 70]. Consistency could not be formally evaluated for any NMA conducted because of a lack of closed loops informed by more than one RCT [19]. Additional information is contained within **Appendix I in S2 File**.

Relative to placebo, we found that low-dose methylphenidate was significantly better at improving clinical response, as measured by clinicians, by both continuous and dichotomous measures (**Table 5**). Standard-dose atomoxetine, standard-dose sustained-release methylphenidate, and low-dose methylphenidate were significantly better than placebo at improving clinical response by continuous assessment, with no significant difference in the number of responders. Compared with patients on placebo and those on high-dose sustained release methylphenidate, the number of clinical responders was significantly higher in the low-dose methylphenidate group. Caution is urged when interpreting these results, however, given that all estimates for low-dose methylphenidate were informed by a single study [26] and the wide credible interval associated with the effect estimate for this treatment compared to its high-dose sustained-release counterpart (RR 8.43, 95% Crl 1.04 to 118.40) reflects a high level of uncertainty. No other significant differences relative to placebo or among the pharmacotherapies were detected.

**Table 5:** Clinician-reported clinical response in trials with a treatment duration of at least 12 weeks–indirect comparison of ADHD pharmacotherapies.

**A) CONTINUOUS measure of response**							
	Mean difference (95% credible interval)*						
	**Placebo**	**MAS-XR-HD**	**ATX-STD**	**GUAN-STD**	**MPH-OROS-STD**	**MPH-ER-STD**	**MPH-SR-STD**
**Placebo**	—						
**MAS-XR-HD**	-4.2	—					
	(-12.1, 3.5)						
**ATX-STD**	**-3.7**	0.6	—				
	**(-6.7, -0.9)**	(-7.8, 8.8)					
**GUAN-STD**	-0.6	3.6	3.1	—			
	(-9.4, 8.3)	(-8.2, 15.5)	(-6.1, 12.6)				
**MPH-OROS-STD**	-1.4	2.8	2.3	-0.8	—		
	(-7.0, 4.4)	(-6.7, 12.6)	(-3.9, 8.9)	(-11.4, 9.8)			
**MPH-ER-STD**	-3.9	0.3	-0.2	-3.3	-2.5	—	
	(-11.5, 3.7)	(-10.6, 11.3)	(-8.3, 8.0)	(-15.1, 8.4)	(-12.2, 6.8)		
**MPH-SR-STD**	**-5.7**	-1.5	-2	-5.1	-4.3	-1.8	—
	**(-11.2, -0.3)**	(-11.1, 8.0)	(-8.1, 4.2)	(-15.5, 5.1)	(-12.4, 3.5)	(-11.1, 7.5)	
**MPH-LD**	**-10.4**	-6.2	-6.8	-9.9	-9.1	-6.5	-4.7
	**(-19.0, -2.1)**	(-17.6, 5.2)	(-15.7, 2.2)	(-22.2, 2.4)	(-19.5, 0.9)	(-17.8, 4.8)	(-14.8, 5.3)
Note: ATX = atomoxetine, GUAN = guanfacine, HD = high dose, LD = low dose, MAS-XR =mixed amphetamine salts, MPH = methylphenidate, OROS = osmotic-release oral system, ER = extended release, SR = sustained release, STD = standard dose.							
*Random-effects model. Pooled mean differences expressed on the Conners’ ADULT ADHD Rating Scale-Self (CAARS-S), short form. A negative value indicates improvement in clinical response. Statistically significant changes are indicated by use of bold and colour (green indicates that the row treatment is significantly better than the column treatment). White indicates no significant difference between treatments.							
**B) DICHOTOMOUS (treatment response yes versus no)**							
	Relative risk (95% credible interval)*						
	**Placebo**	**ATX-STD**	**BUP-SR-STD**	**MPH-OROS-STD**	**MPH-ER-STD**	**MPH-SR-STD**	**MPH-SR- HD**
**Placebo**	—						
**ATX-STD**	1.99	—					
	(0.96, 4.14)						
**BUP-SR-STD**	0.72	0.37	—				
	(0.06, 3.27)	(0.02, 1.66)					
**MPH-OROS-STD**	1.51	0.78	2.01	—			
	(0.18, 3.95)	(0.07, 2.32)	(0.16, 28.71)				
**MPH-ER-STD**	1.58	0.87	2.21	1.1	—		
	(0.42, 2.98)	(0.09, 2.42)	(0.20, 30.14)	(0.13, 10.74)			
**MPH-SR-STD**	1.14	0.59	1.55	0.77	0.7	—	
	(0.11, 3.78)	(0.05, 2.12)	(0.11, 24.80)	(0.07, 8.49)	(0.06, 6.97)		
**MPH-SR-HD**	0.41	0.21	0.58	0.28	0.26	0.37	—
	(0.03, 2.70	(0.01, 1.30)	(0.06, 5.20)	(0.02, 4.13)	(0.02, 3.69)	(0.02, 6.61)	
**MPH-LD**	**3.57**	1.73	4.68	2.26	2.04	2.98	**8.43**
	**(1.13, 5.44)**	(0.45, 3.85)	(0.84, 59.28)	(0.58, 20.17)	(0.54, 15.85)	(0.67, 30.78)	**(1.04,118.40)**

Note: ATX = atomoxetine, ER = extended release, HD = high dose, LD = low-dose, MPH = methylphenidate, OROS = osmotic-release oral system, SR = sustained release, STD = standard dose.

*Random-effects model. A relative risk greater than one (green) indicates that a statistically significantly higher proportion of participants in the row treatment achieved treatment response relative to the column treatment. A relative risk lower than one (red) indicates that a statistically significantly higher proportion of participants in the column treatment achieved treatment response relative to the row treatment. White indicates no statistically significant difference between treatments.

*Executive function*. No studies with a treatment duration of 12 weeks or longer assessed executive function. Data from 12 RCTs (n = 3024) that assessed executive function on a continuous scale after treatment for 2–10 weeks (**Table 2**) [27, 30, 33, 48–50, 52, 63, 67, 73, 74, 100] were analyzed by pair-wise MA. Just over half (58%) of these trials [30, 33, 48–50, 52, 100] were judged to be at low ROB for blinding. Compared with placebo, we found that the use of any ADHD pharmacotherapy was associated with a moderate statistically significant improvement in executive function (MD on BRIEF-A –5.72, 95% CI –7.15 to –4.29; *I*^*2*^ = 58%) (**Appendix J in S2 File**).

*Quality of life*. In total, quality of life was assessed in five RCTs with a treatment duration of 12 weeks or longer [25, 34–36, 70]. Of these, all involved the use of standard-dose atomoxetine compared with placebo or no treatment (n = 1862) (**Table 2**). Only one trial [25] was judged to be at low ROB for blinding; two others [34, 70] were judged to be at high ROB, and the remaining two [35, 36] were judged as unclear. Pair-wise MA showed that atomoxetine was associated with a small favourable improvement response when compared to placebo (MD on AAQoL scale 4.21, 95% CI 2.04 to 6.38) (**Appendix K in S2 File**).

*Driving behaviour*. Five studies evaluated driving behavior among participants with ADHD [23, 25, 34, 70, 84] (**Appendix L in S2 File**). Of these, three studies reported no significant difference in self-assessed driving behaviour following treatment with atomoxetine [25, 34, 70]. One study reported no self-assessed difference in driving anger following treatment with atomoxetine [84]. Two studies reported improved self-reported driving following treatment (lisdexamfetamine, atomoxetine) [23, 84].

Two studies reported no change in clinician-reported driving behaviour following treatment with atomoxetine [25, 84], while one study reported improved driving following treatment with atomoxetine [34]. Of note, the study that reported an improvement had a longer treatment duration (six months) compared to the studies that reported no difference (four or 12 weeks).

#### Harms

Network meta-analyses for harms were not robust owing to the large number of zero-event counts in the networks for each of these outcomes. As such, only pair-wise MAs were performed for serious adverse events, withdrawals due to adverse events, and treatment discontinuations. Hospitalizations and cardiovascular adverse events are summarized narratively. No studies reported on emergency room visits during the study period.

*Serious adverse events*. In total 33 studies reported serious adverse events; of these, five involved a treatment duration of at least 12 weeks. Among RCTs involving treatment for at least 12 weeks, three [32, 35, 44] reported the occurrence of serious adverse events during the treatment period, while two additional RCTs [26, 80] reported that no serious adverse events had occurred during the study (Table **2**). Compared with placebo, there was no significant difference in the risk of a serious adverse event with use of an ADHD pharmacotherapy (RR 1.46, 95% CI 0.52 to 4.09; *I*^2^ = 0%) (**Appendix M in S2 File**). A total of 16 RCTs with any treatment duration reported the occurrence of serious adverse events [32, 34, 35, 44, 47, 49, 50, 52, 56, 63, 68, 72, 73, 75, 78, 82], while an additional 17 RCTs reported that no serious adverse events had occurred during the study [21, 26, 31, 33, 40, 41, 48, 53, 76–78, 80, 81, 88, 93, 101, 103]. Compared with placebo, there was no significant difference in the risk of a serious adverse event with use of an ADHD pharmacotherapy (RR 1.21, 95% CI 0.67 to 2.18; *I*^2^ = 0%).

*Withdrawals due to adverse events*. Seventeen RCTs with a treatment duration of at least 12 weeks reported withdrawals due to adverse events (n = 3650) [25, 26, 28, 32, 34–36, 44, 58–60, 62, 69, 70, 79, 80, 102]. Compared with placebo, there was a significant increase in withdrawals due to adverse events among participants who received an ADHD medication, with moderate heterogeneity between trials (RR 2.30, 95% CI 1.62 to 3.25; *I*^2^ = 37%) (**Appendix N in S2 File**). Among the 52 RCTs (n = 10,726) of any treatment duration [21–23, 25, 26, 28, 31–36, 39–41, 44, 47–50, 52, 53, 55, 56, 58, 59, 62, 63, 65, 67–73, 75, 76, 78–82, 89, 94, 95, 102] reporting on withdrawals, use of an ADHD pharmacotherapy was associated with a higher risk of withdrawal due to an adverse event (RR 2.54, 95% CI 2.14 to 3.03; *I*^2^ = 0%) compared with placebo.

*Treatment discontinuation*. Sixteen RCTs with a treatment duration of at least 12 weeks reported treatment discontinuations (n = 3568) [25, 26, 28, 32, 34–36, 44, 58–60, 62, 69, 70, 79, 80]. Compared with placebo, there was no significant difference in the number of discontinuations between participants who received an ADHD pharmacotherapy and those who received placebo with moderate heterogeneity between trials (RR 1.04, 95% CI 0.91 to 1.20; *I*^*2*^ = 63%) (**Appendix O in S2 File**). The effect estimate was similar among 52 RCTs (n = 9959) with any treatment duration (RR 1.10, 95% CI 1.00 to 1.21; *I*^*2*^ = 46%).

*Hospitalization*. Three studies reported hospitalizations during the study period [60, 72, 73]. In their 2007 study, Spencer et al. [72] reported that two patients randomized to treatment with extended-release methylphenidate each experienced a serious adverse event that required hospitalization (ulcerative colitis/hypovolemic shock, fever/loss of consciousness). Neither patient was withdrawn from the study. In their 2008 study [73], Spencer et al. also reported that one patient assigned to treatment with mixed amphetamine salt group was admitted to hospital with a possible transient ischemic attack. The patient was discharged the following day, with a diagnosis of Tourette syndrome with vocal tic. The investigator disagreed with this diagnosis and felt that transient ischemic attack could not be ruled out.

Levin and colleagues [60] reported that two participants, both in the placebo group, experienced serious adverse events requiring admission to hospital (sexual assault, pneumothorax). Neither of these events was considered by the investigators to be study-related.

*Cardiovascular events*. No studies reported myocardial infarction during the study period. One study [73] reported that one patient experienced a possible transient ischemic attack during the study period, as previously described. No studies reported on cardiovascular death during the treatment period.

### Sensitivity analyses

After removing studies judged to be at unclear or high ROB for blinding for subjective outcomes, some important differences were noted. When only trials at low ROB for blinding were included in the pair-wise meta-analyses, there was no longer a significant difference between ADHD pharmacotherapy and placebo for clinical response (clinician- or patient-reported) (**Appendix P in S2 File).** When the effects of individual ADHD pharmacotherapies were analyzed by network meta-analysis, the beneficial effect of atomoxetine on clinical response (clinician-reported, continuous scale) was no longer evident (**Appendix P in S2 File)**. Similarly, when analyzed as a dichotomous variable (responder yes v. no), the beneficial of effect of atomoxetine was no longer evident. However, in this analysis, a low-dose MPH showed a significant benefit relative to placebo, atomoxetine, bupropion, osmotic-release oral system methylphenidate, and sustained release methylphenidate. This finding was based on a limited number of trials (k = 4) and requires additional investigation.

We further examined the influence of heterogeneity on the effect estimates, for two pair-wise MAs that had considerable heterogeneity: patient-reported clinical response (dichotomous) and clinician-reported clinical response (continuous). We identified a single study [70] that was methodologically different than the others in that it randomized participants to 12 weeks of standard-dose atomoxetine or no treatment; all other trials included participants randomized to an ADHD pharmacotherapy or placebo. After removing this study from the MA of patient-reported clinical response (dichotomous scale), while the *I*^2^ value was reduced (76% to 59%), there was no substantial difference in the overall effect estimate (**Appendix P in S2 File**). Similarly, while the *I*^2^ value for clinician-reported clinical response (continuous measure) was reduced from 78% to 68% after removing this study, there was no substantial difference in the overall effect estimate.

## Discussion

In this study, we performed a comprehensive systematic review of RCTs that assessed the benefits and harms of pharmacotherapies for ADHD in adults. We found that, as a class, ADHD pharmacotherapies were more effective than placebo at improving patient- and clinician-reported clinical response, quality of life, and executive function; however, the clinical importance of these changes is unclear, and when the analyses were restricted to studies at low risk of bias due to blinding, there was no significant difference between ADHD pharmacotherapy and placebo in the meta-analysis, and few differences were evident in the network meta-analyses. Furthermore, when assessed by the GRADE approach, the certainty of the findings for all outcomes was very low to low, indicating that we are uncertain whether there are true differences between ADHD pharmacotherapies owing to the low quality of the available evidence and the high level of uncertainty.

When all studies were considered, atomoxetine was associated with better clinical response compared to most other pharmacotherapies; however, we note that the beneficial effect of atomoxetine was not conserved when only studies at low-risk of bias for blinding were considered. Additionally, participants who received atomoxetine were more likely to discontinue treatment in both shorter- (up to 12 weeks) and longer-term studies. This is consistent with the findings of a previous review which reported increased discontinuation with atomoxetine treatment in shorter-term studies [104]. Similarly, Bushe et al. [104] also noted increased participant discontinuation after short-term treatment with osmotic-release methylphenidate compared with those who received placebo. Our finding of no significant difference in the risk of treatment discontinuation among participants who received this medication for at least 12 weeks may suggest that patients discontinue osmotic-release oral-system methylphenidate at an earlier time point.

Most of the RCTs included in this review included participants who were not naïve to ADHD treatment (**Appendix E in S2 File**). Although this is not surprising, it may complicate the generalization of the study findings to a more broad patient population and may underestimate harms. Similarly, the use of a “washout” period for participants with prior treatment experience complicates the interpretation of individual study findings: effectively, a potentially beneficial treatment may be removed during the washout period and reinstated after randomization, leading to “breaking the blind” and/or overestimation of treatment effects. Despite this, few of the included RCTs assessed whether the blind had been broken during the study period or assessed the impact on subjective outcomes. This is an important consideration for treatments such as methylphenidate, which are associated with detectable effects that may render it difficult to fully blind such studies. Because most trials did not involve use of an active treatment, participants may have been aware of group assignment despite a “double blind” design, which could have introduced bias that was not detected as part of our risk of bias assessment. This inherent problem of maintaining the blind in ADHD trials has been highlighted for studies involving children and adults [105, 106], and contributed to the retraction of a 2014 Cochrane review of methylphenidate for ADHD in adults [107].

We identified a recently published systematic review and NMA by Cortese et al. [108] on a similar topic; however, several methodological differences between studies make direct comparisons with our study difficult. For example, in our NMA, we considered individual pharmacotherapies, formulations, and dose classifications separately, while Cortese et al. [108] grouped together all ADHD pharmacotherapies, regardless of formulation or dose (although some restrictions were applied). Furthermore, whenever possible, we restricted our analyses of benefits to data from studies with a treatment duration of 12 weeks or longer, whereas Cortese et al.’s [108] primary endpoint included outcome data available at times that were “closest to 12 weeks.” In addition, while we completed sensitivity analyses to examine the impact of studies that were judged to be of unclear or high ROB, we included all RCTs regardless as to whether they were open label, single-, or double-blind. In contrast, Cortese et al. [108] limited inclusion to RCTs that were double-blind, which is similar to the approach we adopted in sensitivity analyses. Despite these methodological differences, our finding that atomoxetine was associated with a moderate improvement in clinician-reported clinical response compared with placebo was consistent with the findings of Cortese et al. [108] Similarly, we also found that sustained-release methylphenidate was significantly better than placebo at improving clinical response, which is consistent with Cortese’s [108] findings. However, the effect sizes for each outcome were in the small to moderate range and the clinical importance of such differences is unclear [8, 109].

### Strengths and limitations

The strengths of this review include the use of established systematic review methodology [8] and *a priori* registration of the review protocol. By using NMA methodology, we were able to compare the relative effects of individual ADHD pharmacotherapies, some of which have not been compared in head-to-head trials. This review was undertaken to inform policy recommendations for coverage of ADHD pharmacotherapies on Canadian provincial formularies; however, because we included a broad range of ADHD pharmacotherapies, the results of our review may be of interest to decision-makers in other jurisdictions.

Several limitations should be considered. First, the treatment duration employed in the included RCTs was variable. To minimize heterogeneity due to treatment duration, we restricted the NMAs to studies with a minimum duration of 12 weeks. This approach is consistent with Canadian clinical guidelines to assess outcomes after 12 weeks of treatment; [2] however, it precludes making conclusions about the benefits of short-term treatment. Further, since treatment for ADHD may be life-long, the short-term duration of most trials limits their generalizability to the real-world context. The generalizability of the findings to a larger clinical population is also hampered by the exclusion of participants with psychiatric comorbidities from most included RCTs. Second, the geometry of the evidence networks precluded formal evaluation of inconsistency, and the findings of most outcomes were downgraded in the GRADE assessment because of incoherence, imprecision, indirectness, and within-study bias (risk of bias). We also note the potential for publication bias, as 3 RCTs were included but did not report outcomes of interest for this review. Of these, the NCT records for two studies [110, 111] list quality of life, executive function, and clinical response among the outcome measures; however, these data have not been reported. Furthermore, some RCTs reported clinical response (observer and self-reported) as a dichotomous variable (i.e., number of patients who experienced a clinical response). The categorization of a continuous variable (as “response” or “no response”) introduces additional issues such as information loss, variation in the chosen-cut-point, failure to consider variation in response, and may increase the chance of a false positive result [112]. Third, as with all network meta-analyses, there is a risk of type I error. In particular, because of repeated testing, meta-analyses may particularly susceptible to making claims of efficacy when there is no true effect [113]. Our analyses did not explicitly consider the possibility of type 1 error and this should be considered in the interpretation of the findings, particularly for those where the confidence or credible intervals narrowly includes the null value. Fourth, some commonly prescribed interventions were under-represented in the evidence networks owing to a lack of published RCTs that met the eligibility criteria. Fifth, more than half of the included RCTs were at unclear or high risk of bias for blinding, which may have had an impact on the reliability of subjective data. Additionally, few trials assessed whether blinding was maintained over the treatment period, which may further confound the interpretation of the individual trial results. Sixth, we included only English-language publications, which may have excluded some relevant trials. However, we compared our included studies list to that of a recent systematic review with no language restrictions and did not identify any relevant non-English RCTs [108]. Finally, limited data from RCTs were available for some outcomes, especially for harms and quality of life over a long treatment period. This is highlighted by the small number of RCTs reporting serious adverse events, withdrawals due to adverse events, and treatment discontinuations beyond 12 weeks of treatment.

Because AHDH is a chronic condition, this lack of robust long-term clinical studies represents an important gap in the evidence base, and additional evidence from non-randomized and observational studies may be required to inform policy and clinical decision making. Further, policy decisions about which therapies are most appropriate may need to consider additional relevant factors, which may include burden of disease, therapeutic impact, safety profile, and socioeconomic impact [114]. For individual patients, the choice between treatments should reflect shared decision-making between the clinician and patient, with consideration of the patient’s values, preferences, and individual circumstances, as well as the potential risks and benefits of the treatment options [115].

## Conclusions

Overall, we found that ADHD pharmacotherapies, as a class, improved clinical response relative to placebo; however, most studies were at risk of at least one important source of bias, and the beneficial effects were primarily observed in English-language studies in which participants and/or investigators were aware of treatment assignment. While there was limited evidence of serious harms, harm outcomes were inadequately reported over a long treatment duration. Overall, the certainty of the evidence was very low to low across outcomes, and additional long-term high-quality RCTs are needed to inform on patient-important outcomes such as quality of life and adverse events over a long treatment duration.

## Supporting information

S1 File PRISMA NMA checklist. (DOCX)

S2 File Supplementary online content (Appendices A–P). Appendix A: Literature search strategy. Appendix B: ADHD pharmacotherapies included in the systematic review. Appendix C: Eligible scales for review. Appendix D: Included studies. Appendix E: Detailed inclusion and exclusion criteria. Appendix F: Risk of bias assessment. Appendix G: Publication bias. Appendix H: GRADE assessment. Appendix I: Clinical response. Appendix J: Executive function. Appendix K: Quality of life. Appendix L: Driving behavior. Appendix M: Serious adverse events. Appendix N: Withdrawals due to adverse events. Appendix O: Treatment discontinuation. Appendix P: Sensitivity analyses.
(DOCX)
