# Supplementary material for: Pharmacologic treatment of attention deficit hyperactivity disorder in adults: A systematic review and network meta-analysis
Source: PLoS One. 2020 Oct 21;15(10):e0240584. doi: 10.1371/journal.pone.0240584 (PMC7577505; doi:10.1371/journal.pone.0240584)
Supplement: S2 File — Appendix A: Literature search strategy. Appendix B: ADHD pharmacotherapies included in the systematic review. Appendix C: Eligible scales for review. Appendix D: Included studies. Appendix E: Detailed inclusion and exclusion criteria. Appendix F: Risk of bias assessment. Appendix G: Publication bias. Appendix H: GRADE assessment. Appendix I: Clinical response. Appendix J: Executive function. Appendix K: Quality of life. Appendix L: Driving behavior. Appendix M: Serious adverse events. Appendix N: Withdrawals due to adverse events. Appendix O: Treatment discontinuation. Appendix P: Sensitivity analyses. (DOCX) [file pone.0240584.s002.docx]

**S2 File: Supplementary Online Content (Appendix A to P)**Elliott et al.

Contents

[**Appendix A. Literature search strategy** 2](#_Toc42279390)

[**Appendix B. ADHD pharmacotherapies included in the systematic review** 10](#_Toc42279391)

[**Appendix C. Eligible scales for review** 11](#_Toc42279392)

[**Appendix D. Included studies** 12](#_Toc42279393)

[**Appendix E. Detailed inclusion and exclusion criteria** 16](#_Toc42279394)

[**Appendix F. Risk of bias assessment** 60](#_Toc42279395)

[**Appendix G. Publication bias** 63](#_Toc42279396)

[**Appendix H. GRADE assessment** 67](#_Toc42279397)

[**Appendix I. Clinical response** 73](#_Toc42279398)

[**Appendix J. Executive function** 74](#_Toc42279399)

[**Appendix K. Quality of life** 75](#_Toc42279400)

[**Appendix L. Driving behavior** 76](#_Toc42279401)

[**Appendix M. Serious adverse events** 77](#_Toc42279402)

[**Appendix N. Withdrawals due to adverse events** 79](#_Toc42279403)

[**Appendix O. Treatment discontinuation** 82](#_Toc42279404)

[**Appendix P. Sensitivity analyses** 84](#_Toc42279405)

# **Appendix A. Literature search strategy**

**ADHD – Adults – Update**

Final

2015 Apr 27

[(Note: Numbers in parentheses below reflect the 2015 search; the search was subsequently December 12, 13^th^ (Ovid and Embase) and 23^rd^ (Cochrane Library) 2018]

**OVID**

Database: Ovid MEDLINE(R) In-Process & Other Non-Indexed Citations and Ovid MEDLINE(R) <1946 to Present>, PsycINFO <1806 to April Week 3 2015>, Embase <1980 to 2015 Week 17> Search Strategy:

--------------------------------------------------------------------------------

1 Attention Deficit Disorder with Hyperactivity/dt (14332)

2 (Adderall or amphetamine aspartate or amfetamine aspartate or Obetrol or UNII-H527KAP6L5).tw,kw. (1090)

3 ((amphetamine or amfetamine) adj2 (mixed or mixture or salt or salts)).tw,kw. (526)

4 (atomoxetin* or LY 139603 or HSDB 7352 or strattera or tomoxetine or UNII-ASW034S0B8 or UNII-57WVB6I2W0).tw,kw. (3949)

5 Bupropion/ (17791)

6 (bupropion or amfebutamon* or aplenzin or BRN 2101062 or buproprion or budeprion or buproban or elontril or forfivo or UNII-01ZG3TPX31 or wellbutrin).tw,kw. (10924)

7 Clonidine/ (48922)

8 (Apo-Clonidine or clonidine or capresin or caprysin or catapres* or CLON-IR or CLON-XR or Novo-Clonidine or Nu-Clonidine or kapvay or katapres*).tw,kw. (32796)

9 Dexmethylphenidate/ (570)

10 (dexmethylphenidate or D-MPH or d-threo-methylphenidate or dex methylphenidate or dexmethylphenidate or Focalin or methyl d-phenidate or ritadex or UNII-M32RH9MFGP).tw,kw. (626)

11 Dextroamphetamine/ (18538)

12 (Dextroamphetamine or Dextroamfetamine or Curban or d-Amphetamine or d-Amfetamine or Dexamphetamine or Dexamfetamine or Dexedrine or dextro-Amphetamine or dextro-Amfetamine or DextroStat or Oxydess).tw,kw. (15300)

13 Guanfacine/ (3131)

14 (Guanfacine or BS-100-141 or Estulic or Lon798 or Tenex or Intuniv or EINECS 249-442-8 or UNII-30OMY4G3MK).tw,kw. (2246)

15 (lisdexamfetamine dimesylate or lis-dexamfetamine dimesylate or lisdexamfetamine or elvanse or NRP-104 or NRP104 or SPD-489 or SPD489 or Tyvense or Venvanse or Vyvanse).tw,kw. (800)

16 Methamphetamine/ (23591)

17 (Methamphetamin* or deoxyephedrine or desoxyephedrine or Desoxyn* or Madrine or metamfetamin* or methylamphetamin* or methyl amphetamine* or methylamfetamin* or methyl amfetamin* or N-methylamphetamin* or N-methylamfetamin*).tw,kw. (23718)

18 Methylphenidate/ (25281)

19 (Methylphenidate* or Attenta or Biphentin* or Calocain* or CCRIS 6258 or Centedrin* or Concerta* or Daytrana* or Dexmethylphenidate* or d-methylphenidate* or d-MPH or EINECS 206-065-3 or EINECS 204-028-6 or Equasym* or EqXL or HSDB 3126 or Medikinet or Meridil* or Metadate* or Methyl phenidate* or Methylin* or MPH or NCI-C56280 or NSC-169868 or OROS-MPH or Quillivant* or Phenidylate* or Rilaline* or Ritalin* or Ritaphen or Rubifen or Tranquilyn or Tsentedrin* or UNII-4B3SC438HI).tw,kw. (25380)

20 (Apo-methylphenidate* or Novo-methylphenidate* or PHL-methylphenidate* or PMS-methylphenidate* or Ratio-methylphenidate* or Teva-methylphenidate).tw,kw. (9)

21 (Apo-MPH or Novo-MPH or PHL-MPH or PMS-MPH or Ratio-MPH or Teva-MPH).tw,kw. (3)

22 (modafinil or alertec or benzhydrylsulfinylacetamide or CRL 40476 or modavigil or modiodal or provigil or sparlon or vigil).tw,kw. (4745)

23 or/1-22 (164264)

24 Attention Deficit Disorder with Hyperactivity/ (71284)

25 attention deficit*.tw,kw. (68793)

26 ADHD.tw,kw. (56766)

27 hyperkinetic syndrome*.tw,kw. (886)

28 or/24-27 (99185)

29 23 and 28 (23355)

30 exp Adult/ (11392812)

31 (adult or adults or adulthood or middle age$1).tw,kw. (2313589)

32 (older adj2 (age$1 or female$1 or male$1 or patient$1 or person$1 or people$1 or population$1)).tw,kw. (300191)

33 (senior$1 or elderly or geriatric* or gerontolog*).tw,kw. (640253)

34 or/30-33 (12960143)

35 29 and 34 (6522)

36 (controlled clinical trial or randomized controlled trial).pt. (476586)

37 clinical trials as topic.sh. (172295)

38 (randomi#ed or randomly or RCT$1 or placebo*).tw. (1647669)

39 ((singl* or doubl* or trebl* or tripl*) adj (mask* or blind* or dumm*)).tw. (323401)

40 trial.ti. (331134)

41 or/36-40 (2063668)

42 35 and 41 (1777)

43 exp Animals/ not (exp Animals/ and Humans/) (8392903)

44 42 not 43 (1757)

45 (comment or editorial or interview or news).pt. (1529756)

46 (letter not (letter and randomized controlled trial)).pt. (1744910)

47 44 not (45 or 46) (1749)

48 (2011* or 2012* or 2013* or 2014* or 2015*).dc. (4427196)

49 47 and 48 (238)

50 49 use prmz (238)

51 attention deficit disorder/dt [Drug Therapy] (14905)

52 amphetamine plus dexamphetamine/ (92)

53 (Adderall or amphetamine aspartate or amfetamine aspartate or Obetrol or UNII-H527KAP6L5).tw,kw. (1090)

54 ((amphetamine or amfetamine) adj2 (mixed or mixture or salt or salts)).tw,kw. (526)

55 atomoxetine/ (4137)

56 (atomoxetin* or LY 139603 or HSDB 7352 or strattera or tomoxetine or UNII-ASW034S0B8 or UNII-57WVB6I2W0).tw,kw. (3949)

57 amfebutamone/ (16973)

58 (bupropion or amfebutamon* or aplenzin or BRN 2101062 or buproprion or budeprion or buproban or elontril or forfivo or UNII-01ZG3TPX31 or wellbutrin).tw,kw. (10924)

59 clonidine/ (48922)

60 (Apo-Clonidine or clonidine or capresin or caprysin or catapres* or CLON-IR or CLON-XR or Novo-Clonidine or Nu-Clonidine or kapvay or katapres*).tw,kw. (32796)

61 dexmethylphenidate/ (570)

62 (dexmethylphenidate or D-MPH or d-threo-methylphenidate or dex methylphenidate or dexmethylphenidate or Focalin or methyl d-phenidate or ritadex or UNII-M32RH9MFGP).tw,kw. (626)

63 dexamphetamine/ (18538)

64 (Dextroamphetamine or Dextroamfetamine or Curban or d-Amphetamine or d-Amfetamine or Dexamphetamine or Dexamfetamine or Dexedrine or dextro-Amphetamine or dextro-Amfetamine or DextroStat or Oxydess).tw,kw. (15300)

65 guanfacine/ (3131)

66 (Guanfacine or BS-100-141 or Estulic or Lon798 or Tenex or Intuniv or EINECS 249-442-8 or UNII-30OMY4G3MK).tw,kw. (2246)

67 lisdexamfetamine/ (636)

68 (lisdexamfetamine dimesylate or lis-dexamfetamine dimesylate or lisdexamfetamine or elvanse or NRP-104 or NRP104 or SPD-489 or SPD489 or Tyvense or Venvanse or Vyvanse).tw,kw. (800)

69 methamphetamine/ (23591)

70 (Methamphetamin* or deoxyephedrine or desoxyephedrine or Desoxyn* or Madrine or metamfetamin* or methylamphetamin* or methyl amphetamine* or methylamfetamin* or methyl amfetamin* or N-methylamphetamin* or N-methylamfetamin*).tw,kw. (23718)

71 methylphenidate/ (25281)

72 (Methylphenidate* or Attenta or Biphentin* or Calocain* or CCRIS 6258 or Centedrin* or Concerta* or Daytrana* or Dexmethylphenidate* or d-methylphenidate* or d-MPH or EINECS 206-065-3 or EINECS 204-028-6 or Equasym* or EqXL or HSDB 3126 or Medikinet or Meridil* or Metadate* or Methyl phenidate* or Methylin* or MPH or NCI-C56280 or NSC-169868 or OROS-MPH or Quillivant* or Phenidylate* or Rilaline* or Ritalin* or Ritaphen or Rubifen or Tranquilyn or Tsentedrin* or UNII-4B3SC438HI).tw,kw. (25380)

73 (Apo-methylphenidate* or Novo-methylphenidate* or PHL-methylphenidate* or PMS-methylphenidate* or Ratio-methylphenidate* or Teva-methylphenidate).tw,kw. (9)

74 (Apo-MPH or Novo-MPH or PHL-MPH or PMS-MPH or Ratio-MPH or Teva-MPH).tw,kw. (3)

75 modafinil/ (4169)

76 (modafinil or alertec or benzhydrylsulfinylacetamide or CRL 40476 or modavigil or modiodal or provigil or sparlon or vigil).tw,kw. (4745)

77 or/51-76 (165695)

78 attention deficit disorder/ (65594)

79 attention deficit*.tw,kw. (68793)

80 ADHD.tw,kw. (56766)

81 hyperkinetic syndrome*.tw,kw. (886)

82 or/78-81 (100044)

83 77 and 82 (23659)

84 adult/ (8809272)

85 (adult or adults or adulthood or middle age$1).tw,kw. (2313589)

86 (older adj2 (age$1 or female$1 or male$1 or patient$1 or person$1 or people$1 or population$1)).tw,kw. (300191)

87 (senior$1 or elderly or geriatric* or gerontolog*).tw,kw. (640253)

88 or/84-87 (10817223)

89 83 and 88 (6484)

90 randomized controlled trial/ or controlled clinical trial/ (981326)

91 exp "clinical trial (topic)"/ (142239)

92 (randomi#ed or randomly or RCT$1 or placebo*).tw. (1647669)

93 ((singl* or doubl* or trebl* or tripl*) adj (mask* or blind* or dumm*)).tw. (323401)

94 trial.ti. (331134)

95 or/90-94 (2224416)

96 89 and 95 (1851)

97 exp animal experimentation/ or exp models animal/ or exp animal experiment/ or nonhuman/ or exp vertebrate/ (38322153)

98 exp humans/ or exp human experimentation/ or exp human experiment/ (29583693)

99 97 not 98 (8740045)

100 96 not 99 (1835)

101 (letter not (letter and randomized controlled trial)).pt. (1744910)

102 editorial.pt. (846129)

103 100 not (101 or 102) (1826)

104 (2011* or 2012* or 2013* or 2014* or 2015*).dd. (6240425)

105 103 and 104 (408)

106 105 use emez (408)

107 exp Attention Deficit Disorder/ (80606)

108 drug therapy/ (446138)

109 107 and 108 (4646)

110 (Adderall or amphetamine aspartate or amfetamine aspartate or Obetrol or UNII-H527KAP6L5).tw. (1084)

111 atomoxetine/ (4137)

112 (atomoxetin* or LY 139603 or HSDB 7352 or strattera or tomoxetine or UNII-ASW034S0B8 or UNII-57WVB6I2W0).tw. (3891)

113 bupropion/ (17791)

114 (bupropion or amfebutamon* or aplenzin or BRN 2101062 or buproprion or budeprion or buproban or elontril or forfivo or UNII-01ZG3TPX31 or wellbutrin).tw. (10829)

115 clonidine/ (48922)

116 (Apo-Clonidine or clonidine or capresin or caprysin or catapres* or CLON-IR or CLON-XR or Novo-Clonidine or Nu-Clonidine or kapvay or katapres*).tw. (32594)

117 (dexmethylphenidate or D-MPH or d-threo-methylphenidate or dex methylphenidate or dexmethylphenidate or Focalin or methyl d-phenidate or ritadex or UNII-M32RH9MFGP).tw. (625)

118 dextroamphetamine/ (18538)

119 (Dextroamphetamine or Dextroamfetamine or Curban or d-Amphetamine or d-Amfetamine or Dexamphetamine or Dexamfetamine or Dexedrine or dextro-Amphetamine or dextro-Amfetamine or DextroStat or Oxydess).tw. (15106)

120 (Guanfacine or BS-100-141 or Estulic or Lon798 or Tenex or Intuniv or EINECS 249-442-8 or UNII-30OMY4G3MK).tw. (2224)

121 (lisdexamfetamine dimesylate or lis-dexamfetamine dimesylate or lisdexamfetamine or elvanse or NRP-104 or NRP104 or SPD-489 or SPD489 or Tyvense or Venvanse or Vyvanse).tw. (796)

122 methamphetamine/ (23591)

123 (Methamphetamin* or deoxyephedrine or desoxyephedrine or Desoxyn* or Madrine or metamfetamin* or methylamphetamin* or methyl amphetamine* or methylamfetamin* or methyl amfetamin* or N-methylamphetamin* or N-methylamfetamin*).tw. (23405)

124 methylphenidate/ (25281)

125 (Methylphenidate* or Attenta or Biphentin* or Calocain* or CCRIS 6258 or Centedrin* or Concerta* or Daytrana* or Dexmethylphenidate* or d-methylphenidate* or d-MPH or EINECS 206-065-3 or EINECS 204-028-6 or Equasym* or EqXL or HSDB 3126 or Medikinet or Meridil* or Metadate* or Methyl phenidate* or Methylin* or MPH or NCI-C56280 or NSC-169868 or OROS-MPH or Quillivant* or Phenidylate* or Rilaline* or Ritalin* or Ritaphen or Rubifen or Tranquilyn or Tsentedrin* or UNII-4B3SC438HI).tw. (25034)

126 (Apo-methylphenidate* or Novo-methylphenidate* or PHL-methylphenidate* or PMS-methylphenidate* or Ratio-methylphenidate* or Teva-methylphenidate).tw. (9)

127 (Apo-MPH or Novo-MPH or PHL-MPH or PMS-MPH or Ratio-MPH or Teva-MPH).tw. (3)

128 (modafinil or alertec or benzhydrylsulfinylacetamide or CRL 40476 or modavigil or modiodal or provigil or sparlon or vigil).tw. (4683)

129 or/109-128 (161779)

130 exp attention deficit disorder/ (80606)

131 attention deficit*.tw. (67874)

132 ADHD.tw. (55909)

133 hyperkinetic syndrome*.tw. (874)

134 or/130-133 (100043)

135 129 and 134 (21248)

136 limit 135 to adulthood <18+ years> [Limit not valid in Ovid MEDLINE(R),Ovid MEDLINE(R) In-Process,Embase; records were retained] (17552)

137 (adult or adults or adulthood or middle age$1).tw. (2306217)

138 (older adj2 (age$1 or female$1 or male$1 or patient$1 or person$1 or people$1 or population$1)).tw. (299374)

139 (senior$1 or elderly or geriatric* or gerontolog*).tw. (619576)

140 or/137-139 (3021145)

141 135 and 140 (4597)

142 136 or 141 (18027)

143 clinical trials/ (46084)

144 (randomi#ed or randomly or RCT$1 or placebo*).tw. (1647669)

145 ((singl* or doubl* or trebl* or tripl*) adj (mask* or blind* or dumm*)).tw. (323401)

146 trial.ti. (331134)

147 or/143-146 (1831228)

148 142 and 147 (4038)

149 exp Animals/ not (exp Animals/ and Humans/) (8392903)

150 148 not 149 (3980)

151 (2011* or 2012* or 2013* or 2014* or 2015*).up. (31219004)

152 150 and 151 (2511)

153 152 use prmz (1448)

154 152 use emez (884)

155 152 not (153 or 154) (179)

156 50 or 106 or 155 (825)

157 remove duplicates from 156 (515) [UNIQUE REFERENCES]

158 157 use prmz (226) [MEDLINE UNIQUE REFS]

159 157 use emez (233) [EMBASE UNIQUE REFS]

160 157 not (158 or 159) (56) [PSYCINFO UNIQUE REFS]

Bupropion Search (overlap with main update, removed)

OVID

Database: Ovid MEDLINE(R) In-Process & Other Non-Indexed Citations and Ovid MEDLINE(R) <1946 to Present>, PsycINFO <1806 to April Week 3 2015>, Embase <1980 to 2015 Week 17> Search Strategy:

--------------------------------------------------------------------------------

1 Attention Deficit Disorder with Hyperactivity/dt (14332)

2 (Adderall or amphetamine aspartate or amfetamine aspartate or Obetrol or UNII-H527KAP6L5).tw,kw. (1090)

3 ((amphetamine or amfetamine) adj2 (mixed or mixture or salt or salts)).tw,kw. (526)

4 (atomoxetin* or LY 139603 or HSDB 7352 or strattera or tomoxetine or UNII-ASW034S0B8 or UNII-57WVB6I2W0).tw,kw. (3949)

5 Bupropion/ (17791)

6 (bupropion or amfebutamon* or aplenzin or BRN 2101062 or buproprion or budeprion or buproban or elontril or forfivo or UNII-01ZG3TPX31 or wellbutrin).tw,kw. (10924)

7 Clonidine/ (48922)

8 (Apo-Clonidine or clonidine or capresin or caprysin or catapres* or CLON-IR or CLON-XR or Novo-Clonidine or Nu-Clonidine or kapvay or katapres*).tw,kw. (32796)

9 Dexmethylphenidate/ (570)

10 (dexmethylphenidate or D-MPH or d-threo-methylphenidate or dex methylphenidate or dexmethylphenidate or Focalin or methyl d-phenidate or ritadex or UNII-M32RH9MFGP).tw,kw. (626)

11 Dextroamphetamine/ (18538)

12 (Dextroamphetamine or Dextroamfetamine or Curban or d-Amphetamine or d-Amfetamine or Dexamphetamine or Dexamfetamine or Dexedrine or dextro-Amphetamine or dextro-Amfetamine or DextroStat or Oxydess).tw,kw. (15300)

13 Guanfacine/ (3131)

14 (Guanfacine or BS-100-141 or Estulic or Lon798 or Tenex or Intuniv or EINECS 249-442-8 or UNII-30OMY4G3MK).tw,kw. (2246)

15 (lisdexamfetamine dimesylate or lis-dexamfetamine dimesylate or lisdexamfetamine or elvanse or NRP-104 or NRP104 or SPD-489 or SPD489 or Tyvense or Venvanse or Vyvanse).tw,kw. (800)

16 Methamphetamine/ (23591)

17 (Methamphetamin* or deoxyephedrine or desoxyephedrine or Desoxyn* or Madrine or metamfetamin* or methylamphetamin* or methyl amphetamine* or methylamfetamin* or methyl amfetamin* or N-methylamphetamin* or N-methylamfetamin*).tw,kw. (23718)

18 Methylphenidate/ (25281)

19 (Methylphenidate* or Attenta or Biphentin* or Calocain* or CCRIS 6258 or Centedrin* or Concerta* or Daytrana* or Dexmethylphenidate* or d-methylphenidate* or d-MPH or EINECS 206-065-3 or EINECS 204-028-6 or Equasym* or EqXL or HSDB 3126 or Medikinet or Meridil* or Metadate* or Methyl phenidate* or Methylin* or MPH or NCI-C56280 or NSC-169868 or OROS-MPH or Quillivant* or Phenidylate* or Rilaline* or Ritalin* or Ritaphen or Rubifen or Tranquilyn or Tsentedrin* or UNII-4B3SC438HI).tw,kw. (25380)

20 (Apo-methylphenidate* or Novo-methylphenidate* or PHL-methylphenidate* or PMS-methylphenidate* or Ratio-methylphenidate* or Teva-methylphenidate).tw,kw. (9)

21 (Apo-MPH or Novo-MPH or PHL-MPH or PMS-MPH or Ratio-MPH or Teva-MPH).tw,kw. (3)

22 (modafinil or alertec or benzhydrylsulfinylacetamide or CRL 40476 or modavigil or modiodal or provigil or sparlon or vigil).tw,kw. (4745)

23 or/1-22 (164264)

24 Attention Deficit Disorder with Hyperactivity/ (71284)

25 attention deficit*.tw,kw. (68793)

26 ADHD.tw,kw. (56766)

27 hyperkinetic syndrome*.tw,kw. (886)

28 or/24-27 (99185)

29 23 and 28 (23355)

30 exp Adult/ (11392812)

31 (adult or adults or adulthood or middle age$1).tw,kw. (2313589)

32 (older adj2 (age$1 or female$1 or male$1 or patient$1 or person$1 or people$1 or population$1)).tw,kw. (300191)

33 (senior$1 or elderly or geriatric* or gerontolog*).tw,kw. (640253)

34 or/30-33 (12960143)

35 29 and 34 (6522)

36 (controlled clinical trial or randomized controlled trial).pt. (476586)

37 clinical trials as topic.sh. (172295)

38 (randomi#ed or randomly or RCT$1 or placebo*).tw. (1647669)

39 ((singl* or doubl* or trebl* or tripl*) adj (mask* or blind* or dumm*)).tw. (323401)

40 trial.ti. (331134)

41 or/36-40 (2063668)

42 35 and 41 (1777)

43 exp Animals/ not (exp Animals/ and Humans/) (8392903)

44 42 not 43 (1757)

45 (comment or editorial or interview or news).pt. (1529756)

46 (letter not (letter and randomized controlled trial)).pt. (1744910)

47 44 not (45 or 46) (1749)

48 (2011* or 2012* or 2013* or 2014* or 2015*).dc. (4427196)

49 47 and 48 (238)

50 49 use prmz (238)

51 attention deficit disorder/dt [Drug Therapy] (14905)

52 amphetamine plus dexamphetamine/ (92)

53 (Adderall or amphetamine aspartate or amfetamine aspartate or Obetrol or UNII-H527KAP6L5).tw,kw. (1090)

54 ((amphetamine or amfetamine) adj2 (mixed or mixture or salt or salts)).tw,kw. (526)

55 atomoxetine/ (4137)

56 (atomoxetin* or LY 139603 or HSDB 7352 or strattera or tomoxetine or UNII-ASW034S0B8 or UNII-57WVB6I2W0).tw,kw. (3949)

57 amfebutamone/ (16973)

58 (bupropion or amfebutamon* or aplenzin or BRN 2101062 or buproprion or budeprion or buproban or elontril or forfivo or UNII-01ZG3TPX31 or wellbutrin).tw,kw. (10924)

59 clonidine/ (48922)

60 (Apo-Clonidine or clonidine or capresin or caprysin or catapres* or CLON-IR or CLON-XR or Novo-Clonidine or Nu-Clonidine or kapvay or katapres*).tw,kw. (32796)

61 dexmethylphenidate/ (570)

62 (dexmethylphenidate or D-MPH or d-threo-methylphenidate or dex methylphenidate or dexmethylphenidate or Focalin or methyl d-phenidate or ritadex or UNII-M32RH9MFGP).tw,kw. (626)

63 dexamphetamine/ (18538)

64 (Dextroamphetamine or Dextroamfetamine or Curban or d-Amphetamine or d-Amfetamine or Dexamphetamine or Dexamfetamine or Dexedrine or dextro-Amphetamine or dextro-Amfetamine or DextroStat or Oxydess).tw,kw. (15300)

65 guanfacine/ (3131)

66 (Guanfacine or BS-100-141 or Estulic or Lon798 or Tenex or Intuniv or EINECS 249-442-8 or UNII-30OMY4G3MK).tw,kw. (2246)

67 lisdexamfetamine/ (636)

68 (lisdexamfetamine dimesylate or lis-dexamfetamine dimesylate or lisdexamfetamine or elvanse or NRP-104 or NRP104 or SPD-489 or SPD489 or Tyvense or Venvanse or Vyvanse).tw,kw. (800)

69 methamphetamine/ (23591)

70 (Methamphetamin* or deoxyephedrine or desoxyephedrine or Desoxyn* or Madrine or metamfetamin* or methylamphetamin* or methyl amphetamine* or methylamfetamin* or methyl amfetamin* or N-methylamphetamin* or N-methylamfetamin*).tw,kw. (23718)

71 methylphenidate/ (25281)

72 (Methylphenidate* or Attenta or Biphentin* or Calocain* or CCRIS 6258 or Centedrin* or Concerta* or Daytrana* or Dexmethylphenidate* or d-methylphenidate* or d-MPH or EINECS 206-065-3 or EINECS 204-028-6 or Equasym* or EqXL or HSDB 3126 or Medikinet or Meridil* or Metadate* or Methyl phenidate* or Methylin* or MPH or NCI-C56280 or NSC-169868 or OROS-MPH or Quillivant* or Phenidylate* or Rilaline* or Ritalin* or Ritaphen or Rubifen or Tranquilyn or Tsentedrin* or UNII-4B3SC438HI).tw,kw. (25380)

73 (Apo-methylphenidate* or Novo-methylphenidate* or PHL-methylphenidate* or PMS-methylphenidate* or Ratio-methylphenidate* or Teva-methylphenidate).tw,kw. (9)

74 (Apo-MPH or Novo-MPH or PHL-MPH or PMS-MPH or Ratio-MPH or Teva-MPH).tw,kw. (3)

75 modafinil/ (4169)

76 (modafinil or alertec or benzhydrylsulfinylacetamide or CRL 40476 or modavigil or modiodal or provigil or sparlon or vigil).tw,kw. (4745)

77 or/51-76 (165695)

78 attention deficit disorder/ (65594)

79 attention deficit*.tw,kw. (68793)

80 ADHD.tw,kw. (56766)

81 hyperkinetic syndrome*.tw,kw. (886)

82 or/78-81 (100044)

83 77 and 82 (23659)

84 adult/ (8809272)

85 (adult or adults or adulthood or middle age$1).tw,kw. (2313589)

86 (older adj2 (age$1 or female$1 or male$1 or patient$1 or person$1 or people$1 or population$1)).tw,kw. (300191)

87 (senior$1 or elderly or geriatric* or gerontolog*).tw,kw. (640253)

88 or/84-87 (10817223)

89 83 and 88 (6484)

90 randomized controlled trial/ or controlled clinical trial/ (981326)

91 exp "clinical trial (topic)"/ (142239)

92 (randomi#ed or randomly or RCT$1 or placebo*).tw. (1647669)

93 ((singl* or doubl* or trebl* or tripl*) adj (mask* or blind* or dumm*)).tw. (323401)

94 trial.ti. (331134)

95 or/90-94 (2224416)

96 89 and 95 (1851)

97 exp animal experimentation/ or exp models animal/ or exp animal experiment/ or nonhuman/ or exp vertebrate/ (38322153)

98 exp humans/ or exp human experimentation/ or exp human experiment/ (29583693)

99 97 not 98 (8740045)

100 96 not 99 (1835)

101 (letter not (letter and randomized controlled trial)).pt. (1744910)

102 editorial.pt. (846129)

103 100 not (101 or 102) (1826)

104 (2011* or 2012* or 2013* or 2014* or 2015*).dd. (6240425)

105 103 and 104 (408)

106 105 use emez (408)

107 exp Attention Deficit Disorder/ (80606)

108 drug therapy/ (446138)

109 107 and 108 (4646)

110 (Adderall or amphetamine aspartate or amfetamine aspartate or Obetrol or UNII-H527KAP6L5).tw. (1084)

111 atomoxetine/ (4137)

112 (atomoxetin* or LY 139603 or HSDB 7352 or strattera or tomoxetine or UNII-ASW034S0B8 or UNII-57WVB6I2W0).tw. (3891)

113 bupropion/ (17791)

114 (bupropion or amfebutamon* or aplenzin or BRN 2101062 or buproprion or budeprion or buproban or elontril or forfivo or UNII-01ZG3TPX31 or wellbutrin).tw. (10829)

115 clonidine/ (48922)

116 (Apo-Clonidine or clonidine or capresin or caprysin or catapres* or CLON-IR or CLON-XR or Novo-Clonidine or Nu-Clonidine or kapvay or katapres*).tw. (32594)

117 (dexmethylphenidate or D-MPH or d-threo-methylphenidate or dex methylphenidate or dexmethylphenidate or Focalin or methyl d-phenidate or ritadex or UNII-M32RH9MFGP).tw. (625)

118 dextroamphetamine/ (18538)

119 (Dextroamphetamine or Dextroamfetamine or Curban or d-Amphetamine or d-Amfetamine or Dexamphetamine or Dexamfetamine or Dexedrine or dextro-Amphetamine or dextro-Amfetamine or DextroStat or Oxydess).tw. (15106)

120 (Guanfacine or BS-100-141 or Estulic or Lon798 or Tenex or Intuniv or EINECS 249-442-8 or UNII-30OMY4G3MK).tw. (2224)

121 (lisdexamfetamine dimesylate or lis-dexamfetamine dimesylate or lisdexamfetamine or elvanse or NRP-104 or NRP104 or SPD-489 or SPD489 or Tyvense or Venvanse or Vyvanse).tw. (796)

122 methamphetamine/ (23591)

123 (Methamphetamin* or deoxyephedrine or desoxyephedrine or Desoxyn* or Madrine or metamfetamin* or methylamphetamin* or methyl amphetamine* or methylamfetamin* or methyl amfetamin* or N-methylamphetamin* or N-methylamfetamin*).tw. (23405)

124 methylphenidate/ (25281)

125 (Methylphenidate* or Attenta or Biphentin* or Calocain* or CCRIS 6258 or Centedrin* or Concerta* or Daytrana* or Dexmethylphenidate* or d-methylphenidate* or d-MPH or EINECS 206-065-3 or EINECS 204-028-6 or Equasym* or EqXL or HSDB 3126 or Medikinet or Meridil* or Metadate* or Methyl phenidate* or Methylin* or MPH or NCI-C56280 or NSC-169868 or OROS-MPH or Quillivant* or Phenidylate* or Rilaline* or Ritalin* or Ritaphen or Rubifen or Tranquilyn or Tsentedrin* or UNII-4B3SC438HI).tw. (25034)

126 (Apo-methylphenidate* or Novo-methylphenidate* or PHL-methylphenidate* or PMS-methylphenidate* or Ratio-methylphenidate* or Teva-methylphenidate).tw. (9)

127 (Apo-MPH or Novo-MPH or PHL-MPH or PMS-MPH or Ratio-MPH or Teva-MPH).tw. (3)

128 (modafinil or alertec or benzhydrylsulfinylacetamide or CRL 40476 or modavigil or modiodal or provigil or sparlon or vigil).tw. (4683)

129 or/109-128 (161779)

130 exp attention deficit disorder/ (80606)

131 attention deficit*.tw. (67874)

132 ADHD.tw. (55909)

133 hyperkinetic syndrome*.tw. (874)

134 or/130-133 (100043)

135 129 and 134 (21248)

136 limit 135 to adulthood <18+ years> [Limit not valid in Ovid MEDLINE(R),Ovid MEDLINE(R) In-Process,Embase; records were retained] (17552)

137 (adult or adults or adulthood or middle age$1).tw. (2306217)

138 (older adj2 (age$1 or female$1 or male$1 or patient$1 or person$1 or people$1 or population$1)).tw. (299374)

139 (senior$1 or elderly or geriatric* or gerontolog*).tw. (619576)

140 or/137-139 (3021145)

141 135 and 140 (4597)

142 136 or 141 (18027)

143 clinical trials/ (46084)

144 (randomi#ed or randomly or RCT$1 or placebo*).tw. (1647669)

145 ((singl* or doubl* or trebl* or tripl*) adj (mask* or blind* or dumm*)).tw. (323401)

146 trial.ti. (331134)

147 or/143-146 (1831228)

148 142 and 147 (4038)

149 exp Animals/ not (exp Animals/ and Humans/) (8392903)

150 148 not 149 (3980)

151 (2011* or 2012* or 2013* or 2014* or 2015*).up. (31219004)

152 150 and 151 (2511)

153 152 use prmz (1448)

154 152 use emez (884)

155 152 not (153 or 154) (179)

156 50 or 106 or 155 (825) [ORIGINAL RESULTS – 2011-CURRENT]

157 remove duplicates from 156 (515)

158 157 use prmz (226)

159 157 use emez (233)

160 157 not (158 or 159) (56)

161 5 or 6 (20535)

162 161 and 28 (1363)

163 162 and 34 (548)

164 163 and 41 (111)

165 164 not 43 (110)

166 165 not (45 or 46) (109)

167 166 use prmz (33) [BUPROPION – MEDLINE]

168 57 or 58 (20525)

169 168 and 82 (1374)

170 169 and 88 (552)

171 170 and 95 (129)

172 171 not 99 (128)

173 172 not (101 or 102) (127)

174 173 use emez (83) [BUPRION – EMBASE]

175 113 or 114 (20510)

176 175 and 134 (1369)

177 limit 176 to adulthood <18+ years> [Limit not valid in Ovid MEDLINE(R),Ovid MEDLINE(R) In-Process,Embase; records were retained] (1290)

178 176 and 140 (460)

179 177 or 178 (1319)

180 179 and 147 (196)

181 180 not 149 (195)

182 181 use prmz (39)

183 181 use emez (133)

184 181 not (182 or 183) (23) [BUPROPION – PSYCINFO]

185 167 or 174 or 184 (139) [BUPROPION – ALL DATABASES]

186 185 not 156 (107) [OVERLAP WITH ORIGINAL UPDATE, REMOVED]

187 remove duplicates from 186 (78) [DUPLICATES REMOVED]

188 187 use prmz (24) [MEDLINE UNIQUE HITS]

189 187 use emez (47) [EMBASE UNIQUE HITS]

190 187 not (188 or 189) (7) [PSYCINFO UNIQUE HITS]

***************************

# **Appendix B. ADHD pharmacotherapies included in the systematic review**

| **Active ingredient(s)** | **Formulation** | **Format** | **Standard dose*** |
| --- | --- | --- | --- |
| **Triple-bead mixed amphetamine salts** | Mixed amphetamine salts | Oral capsule | Up to 30 mg/d |
| **Amphetamine mixture*** | Mixed amphetamine salts XR | Extended-release oral capsule | 10–30 mg/d |
| **Atomoxetine hydrochloride** | Atomoxetine | Oral capsule | 40–100 mg/d |
| **Bupropion hydrochloride** | Sustained-release bupropion | Oral tablet | 200–300 mg/d |
|  | Extended-release bupropion | Oral tablet | 200–300 mg/d |
| **Clonidine hydrochloride** | Immediate-release clonidine | Oral tablet | > 45 kg: 0.1–0.4mg |
|  | Extended-release clonidine | Extended-release oral tablet | 0.1–0.4 mg/d |
| **Dexmethylphenidate hydrochloride** | Immediate-release dexmethylphenidate | Oral tablet | 5–20 mg/d |
|  | Extended-release dexmethylphenidate | Extended-release oral capsule | 10–40 mg/d |
| **Dextroamphetamine sulfate** | Immediate-release dextroamphetamine | Oral tablet | 10–60 mg/d |
|  | Sustained-release dextroamphetamine | Sustained-release oral  capsule | 10–60 mg/d |
| **Guanfacine hydrochloride** | Immediate-release guanfacine | Oral tablet | – |
|  | Extended-release guanfacine | Extended-release oral  tablet | 1–7 mg/d |
| **Lisdexamfetamine dimesylate** | Lisdexamfetamine | Oral capsule | 20–60 mg/d |
| **Methamphetamine hydrochloride** | Methamphetamine | Oral tablet | 5–25 mg/d |
| **Methylphenidate hydrochloride** | Methylphenidate osmotic-release oral system | Extended-release oral tablet | 18–72 mg/d |
|  | Methylphenidate CD | Extended-release oral capsule | 20–60 mg/d |
|  | Methylphenidate ER | Extended-release oral tablet | 10–60 mg/d |
|  | Methylphenidate chewable or solution | Oral chewable tablet or solution | 10–60 mg/d |
|  | Immediate-release methylphenidate | Oral tablet | 10–60 mg/d |
|  | Methylphenidate long-acting | Extended-release oral capsule | 10–80 mg/d |
|  | Multilayer-release methylphenidate | Extended-release oral capsule | 10–80 mg/d |
|  | Methylphenidate sustained-release | Extended-release oral  tablet | 10–60 mg/d |
| **Modafinil** | Modafinil | Oral tablet | 100–400 mg/d |
| *Doses were categorized as low, standard, or high, based on an approved or standard range of doses for ADHD treatment as per Health Canada-approved monographs, if available. For treatments not approved in Canada or used off-label, standard doses were based on the US Food and Drug Administration monographs. | | | |

# **Appendix C. Eligible scales for review**

*Note: the list of included scales was based on McDonagh MS, Peterson K, Thakurta S, et al. Drug Class Review: Pharmacologic Treatments for Attention Deficit Hyperactivity Disorder: Final Update 4 Report [Internet]. Portland (OR): Oregon Health & Science University; 2011 Dec. Available from: https://www.ncbi.nlm.nih.gov/books/NBK84419/

***Clinical Response***

ADHD Behaviour Checklist (ADHD Rating scale)

ADHDRS-IV (ADHD Rating Scale IV)

ADHDRS-IV-PI

ADHD Investigator symptom rating scale (AISRS)

Adult Self-Report Scale (ASRS)

Clinical Global Impression Scale (CGI)

Clinical Global Impression: Improvement Scale (CGI-I), Severity Scale (CGI-S), CGI-ADHD-S, CGI ADHD

Conners’ Adult ADHD Rating Scale (CAARS)

Consensus Clinical Response (CCR)

Copeland Symptom Checklist for Adult Attention Deficit Disorder

DuPaul ADHD Rating Scale IV

Intra-individual variability in reaction time

Physician’s Global Rating Scale

Physician’s Target Symptom Scale

Swanson, Kotlin, Agler, M-Flynn and Pelham scale (SKAMP)

Swanson, Nolan, and Pelham-IV Questionnaire (SNAP-IV)

Targeted Adult Attention Deficit Disorder Scale (TAADDS)

Wender-Reimherr Adult Attention Deficit Disorder Scale (WRADDS)

***Quality of life***

Mental Component Summary (MCS)

SF-36 Health Survey

WHO QoL Brief Version (WHO QoL-BRIEF)

Quality of Life Enjoyment and Satisfaction Questionnaire Short Form (Q-LESQ-SF)

Adult ADHD Quality of Life (AAQoL)

ADHD Impact Module Adult Version (AIM-A)

***Executive Function***

Sheehan Disability Scale (SDS)

Swanson, Conners, Milich and Pelham Scale (SCAMP)

Selective Reminding Test (SRT)

Brown ADD Scale (BADDS

Behavior Rating Inventory of Executive Function: Adult (BRIEF-A)

Continuous Paired-Associate Learning Test (CPALT)

Global Assessment Scale (GAS)

Permanent Product Measure of Performance (PERMP)

Trailmaking Test (TMT)

Controlled Oral Word Association (COWAT)

***Driving behaviour***

Driver Behaviour Survey (DBS)

Impaired Driving Score (IDS)

Safe Driving Behaviour Rating Scale

# **Appendix D. Included studies**

**Note:** The full list of excluded studies available from the corresponding author on request

1. Adler LA, Liebowitz M, Kronenberger W, et al. Atomoxetine treatment in adults with attention-deficit/hyperactivity disorder and comorbid social anxiety disorder. *Depress Anxiety.* 2009;26:212-21
2. Adler LA, Clemow DB, Williams DW, et al. Atomoxetine effects on executive function as measured by the BRIEF--a in young adults with ADHD: a randomized, double-blind, placebo-controlled study. *PLoS ONE* 2014;9:e104175
3. Adler LA, Dirks B, Deas P, et al. Self-Reported quality of life in adults with attention-deficit/hyperactivity disorder and executive function impairment treated with lisdexamfetamine dimesylate: a randomized, double-blind, multicenter, placebo-controlled, parallel-group study. *BMC psychiatry.* 2013;13:253
4. Adler LA, Dirks B, Deas PF et al. Lisdexamfetamine dimesylate in adults with attention-deficit/ hyperactivity disorder who report clinically significant impairment in executive function: results from a randomized, double-blind, placebo-controlled study. *Journal of Clinical Psychiatry.* 2013;74:694
5. Adler LA, Goodman D, Weisler R, et al. Effect of lisdexamfetamine dimesylate on sleep in adults with attention-deficit/hyperactivity disorder. *Behav Brain Funct.* 2009; 5:34
6. Adler LA, Goodman DW, Kollins SH, et al. Double-blind, placebo-controlled study of the efficacy and safety of lisdexamfetamine dimesylate in adults with attention-deficit/hyperactivity disorder. *J Clin Psychiatry.* 2008;69:1364-73
7. Adler LA, Spencer T, Brown TE, et al. Once-daily atomoxetine for adult attention-deficit/hyperactivity disorder: a 6-month, double-blind trial. *J Clin Psychopharmacol.* 2009;29:44-50
8. Adler LA, Spencer TJ, Levine LR, et al. Functional outcomes in the treatment of adults with ADHD. *J Atten Disord.* 2008;11:720-7
9. Adler LA, Weisler RH, Goodman DW, et al. Short-term effects of lisdexamfetamine demesylate on cardiovascular parameters in 4-week clinical trial in adults with attention-deficits/hyperactivity disorder. *Journal of Clinical Psychiatry.* 2009;70:1652-61
10. Adler LA, Zimmerman B, Starr HL, et al. Efficacy and safety of OROS methylphenidate in adults with attention-deficit/hyperactivity disorder: a randomized, placebo-controlled, double-blind, parallel group, dose-escalation study. *J Clin Psychopharmacol.* 2009;29:239-47
11. Anouk S, Hyke GT, Cheima B, Marco AB, Esther EB, Henk-Jan MM, et al. Age-dependent effects of methylphenidate on the human dopaminergic system in young vs adult patients with attention-deficit/hyperactivity disorder: A randomized clinical trial. JAMA Psychiatry. 2016;73(9):955-62. doi: http://dx.doi.org/10.1001/jamapsychiatry.2016.1572
12. Arnold VK, Feifel D, Earl CQ, et al. A 9-week, randomized, double-blind, placebo-controlled, parallel-group, dose-finding study to evaluate the efficacy and safety of modafinil as treatment for adults with ADHD. *Journal of attention disorders.* 2014;18:133
13. Babcock T, Dirks B, Adeyi B, et al. Efficacy of lisdexamfetamine dimesylate in adults with attention-deficit/hyperactivity disorder previously treated with amphetamines: analyses from a randomized, double-blind, multicenter, placebo-controlled titration study. *BMC pharmacology & toxicology.* 2012;13:18
14. Bain EE, Robieson W, Pritchett Y, et al. A randomized, double-blind, placebo-controlled phase 2 study of alpha4beta2 agonist ABT-894 in adults with ADHD. *Neuropsychopharmacology.* 2013;38:405
15. Barkley RA, Anderson DL, Kruesi M. A pilot study of the effects of atomoxetine on driving performance in adults with ADHD. *J Atten Disord.* 2007;10:306-16
16. Barkley RA, Murphy KR, O'Connell T, et al. Effects of two doses of methylphenidate on simulator driving performance in adults with attention deficit hyperactivity disorder. *J Safety Res.* 2005;36:121-31
17. Berlin I, Hu MC, Covey LS, et al. Attention-deficit/hyperactivity disorder (ADHD) symptoms, craving to smoke, and tobacco withdrawal symptoms in adult smokers with ADHD. *Drug & Alcohol Dependence.* 2012;124:268
18. Biederman J, Mick E,Fried R, et al. Are stimulants effective in the treatment of executive function deficits? Results from a randomized double blind study of OROS-methylphenidate in adults with ADHD. *European Neuropsychopharmacology.* 2011;21:508
19. Biederman J, Fried R, Hammerness P, et al. The effects of lisdexamfetamine dimesylate on driving behaviors in young adults with ADHD assessed with the Manchester driving behavior questionnaire. *Journal of Adolescent Health.* 2012;51:601
20. Biederman J, Fried R, Hammerness P, et al. The effects of lisdexamfetamine dimesylate on the driving performance of young adults with ADHD: a randomized, double-blind, placebo-controlled study using a validated driving simulator paradigm. *Journal of Psychiatric Research.* 2012;46:484
21. Biederman J, Mick E, Spencer T, et al. Is response to OROS-methylphenidate treatment moderated by treatment with antidepressants or psychiatric comorbidity? A secondary analysis from a large randomized double blind study of adults with ADHD. *CNS Neuroscience & Therapeutics.* 2012;18:126
22. Biederman J, Mick E, Surman C, et al. A randomized, 3-phase, 34-week, double-blind, long-term efficacy study of osmotic-release oral system-methylphenidate in adults with attention-deficit/hyperactivity disorder. *J Clin Psychopharmacol.* 2010;30:549-53
23. Biederman J, Mick E, Surman C, et al. A randomized, placebo-controlled trial of OROS methylphenidate in adults with attention-deficit/hyperactivity disorder. *Biol Psychiatry.* 2006;59:829-35
24. Biehl SC, Merz CJ, Dresler T, et al. Increase or decrease of fMRI activity in adult attention deficit/hyperactivity disorder: Does it depend on task difficulty? *Int J Neuropsychopharmacol* 2016;19(10):1-10.
25. Boonstra AM, Kooij JJ, Oosterlaan J, et al. Hyperactive night and day? Actigraphy studies in adult ADHD: a baseline comparison and the effect of methylphenidate. *Sleep.* 2007;30:433-42
26. Boonstra M, Kooij JJ, Oosterlaan J, et al. Does methylphenidate improve inhibition and other cognitive abilities in adults with childhood-onset ADHD? *J Clin Exp Neuropsychol.* 2005;27:278-98
27. Bottelier MA, Schouw ML, Klomp A, et al. The effects of Psychotropic drugs On Developing brain (ePOD) study: methods and design. *BMC psychiatry.* 2014;14:48
28. Bouffard R, Hechtman L, Minde K, et al. The efficacy of 2 different dosages of methylphenidate in treating adults with attention-deficit hyperactivity disorder. *Can J Psychiatry* 2003;48:546-54
29. Brams M, Giblin J, Gasior M, et al. Effects of open-label lisdexamfetamine dimesylate on self-reported quality of life in adults with ADHD. *Postgraduate Medicine.* 2011;123:99
30. Bron TI, Bijlenga D, Boonstra AM, et al. OROS-methylphenidate efficacy on specific executive functioning deficits in adults with ADHD: a randomized, placebo-controlled cross-over study. *European Neuropsychopharmacology.* 2014;24:519
31. Brown TE, Holdnack J, Saylor K, et al. Effect of atomoxetine on executive function impairments in adults with ADHD. *Journal of attention disorders.* 2011;15:130
32. Buitelaar JK, Casas M, Philipsen A, et al. Functional improvement and correlations with symptomatic improvement in adults with attention deficit hyperactivity disorder receiving long-acting methylphenidate. *Psychological Medicine.* 2012;42:195
33. Buitelaar JK, Kooij JJ, Ramos-Quiroga JA, et al. Predictors of treatment outcome in adults with ADHD treated with OROS methylphenidate. *Progress in Neuro-Psychopharmacology & Biological Psychiatry.* 2011;35:554
34. Butterfield ME, Saal J, Young B, et al. Supplementary guanfacine hydrochloride as a treatment of attention deficit hyperactivity disorder in adults: A double blind, placebo-controlled study. *Psychiatry Research* 2016;236:136-141.
35. Casas M, Rosler M, Sandra Kooij JJ, et al. Efficacy and safety of prolonged-release OROS methylphenidate in adults with attention deficit/hyperactivity disorder: a 13-week, randomized, double-blind, placebo-controlled, fixed-dose study. *World J Biol Psychiatry.* 2013;14:268
36. Collins S. Lisdexamfetamine Dimesylate in the Treatment of Adult ADHD With Anxiety Disorder Comorbidity. *clinicaltrials.gov/show/NCT01863459.*
37. Covey LS, Hu MC, Green CA, et al. An exploration of site effects in a multisite trial of OROS-methylphenidate for smokers with attention deficit/hyperactivity disorder. *American Journal of Drug & Alcohol Abuse.* 2011;37:392
38. Covey LS, Hu MC, Weissman J, et al. Divergence by ADHD subtype in smoking cessation response to OROS-methylphenidate. *Nicotine Tob Res.* 2011;13:1003
39. Covey LS, Hu MC, Winhusen T, et al. OROS-methylphenidate or placebo for adult smokers with attention deficit hyperactivity disorder: racial/ethnic differences. *Drug Alcohol Depend.* 2010;110:156-9
40. Covey LS, Hu MC, Winhusen T, et al. Anxiety and depressed mood decline following smoking abstinence in adult smokers with attention deficit hyperactivity disorder. *J Substance Abuse Treatment* 2015;59:104-8.
41. Durell TM, Adler LA, Williams DW, et al. Atomoxetine treatment of attention-deficit/hyperactivity disorder in young adults with assessment of functional outcomes: a randomized, double-blind, placebo-controlled clinical trial. *Journal of Clinical Psychopharmacology.* 2013;33:45
42. Fan L-Y, Chou T-L, Gau SS-F. Neural correlates of atomoxetine improving inhibitory control and visual processing in drug-naive adults with attention-deficit/hyperactivity. *Human Brain Mapping* 2017 Oct;38(10):4850-4864.
43. Faraone SV, Biederman J, Spencer T, et al. Atomoxetine and stroop task performance in adult attention-deficit/hyperactivity disorder. *J Child Adolesc Psychopharmacol.* 2005;15:664-70
44. Faraone SV, Spencer TJ, Kollins SH, et al. Dose response effects of lisdexamfetamine dimesylate treatment in adults with ADHD: an exploratory study. *Journal of attention disorders.* 2012;16:118
45. Frick G, Yan B, Adler LA. Triple-bead mixed amphetamine salts (SHP465) in adults with ADHD: Results of a phase 3, double-blind, randomized, forced-dose trial. *J Attention Disorders* 2017. doi: 10.1177/1087054717696771.
46. Ginsberg L, Katic A, Adeyi B, et al. Long-term treatment outcomes with lisdexamfetamine dimesylate for adults with attention-deficit/hyperactivity disorder stratified by baseline severity. *Current Medical Research & Opinion.* 2011;27:1097
47. Ginsberg Y, Hirvikoski T, Grann M, et al. Long-term functional outcome in adult prison inmates with ADHD receiving OROS-methylphenidate. *European Archives of Psychiatry & Clinical Neuroscience.* 2012;262:705
48. Ginsberg Y, Lindefors N. Methylphenidate treatment of adult male prison inmates with attention-deficit hyperactivity disorder: randomised double-blind placebo-controlled trial with open-label extension. *British Journal of Psychiatry.* 2012;200:68
49. Goodman DW, Starr HL, Ma Y-W, et al. Randomized, 6-week, placebo-controlled study of treatment for adult attention-deficit/hyperactivity disorder: Individualized dosing of osmotic-release oral system (OROS) methylphenidate with a goal of symptom remission. *J Clin Psychiatry* 2017;78(1):105–114.
50. Goto T, Hirata Y, Trzepacz PT, et al. Efficacy and safety of atomoxetine hydrochloride in Asian adults with ADHD: A multinational 10-week randomized double-blind placebo-controlled Asian study. *J Attention Disorders* 2017;21(2):100-109.
51. Hamedi M, Mohammdi M, Ghaleiha A, et al. Bupropion in adults with Attention-Deficit/Hyperactivity Disorder: a randomized, double-blind study. *Acta Medica Iranica.* 2014;52:675
52. Heffner JL, Lewis DF, Winhusen TM. Osmotic release oral system methylphenidate prevents weight gain during a smoking-cessation attempt in adults with ADHD. *Nicotine Tob Res.* 2013;15:583
53. Herring WJ, Wilens TE, Adler LA et al. Randomized controlled study of the histamine H3 inverse agonist MK-0249 in adult attention-deficit/hyperactivity disorder. *Journal of Clinical Psychiatry.* 2012;73:e891
54. Huss H, Ginsberg Y, Tvedten T, et al. Methylphenidate hydrochloride modified-release in adults with attention deficit hyperactivity disorder: a randomized double-blind placebo-controlled trial. *Advances in Therapy.* 2014;31:44
55. Jain U, Hechtman L, Weiss M, et al. Efficacy of a novel biphasic controlled-release methylphenidate formula in adults with attention-deficit/hyperactivity disorder: results of a double-blind, placebo-controlled crossover study. *J Clin Psychiatry.* 2007;68:268-277.
56. Kay GG, Michaels MA, Pakull B. Simulated driving changes in young adults with ADHD receiving mixed amphetamine salts extended release and atomoxetine. *J Atten Disord.* 2009;12:316-29
57. Kollins SH, English JS, Itchon-Ramos N, et al. A pilot study of lis-dexamfetamine dimesylate (LDX/SPD489) to facilitate smoking cessation in nicotine-dependent adults with ADHD. *Journal of attention disorders.* 2014;18:158
58. Kollins SH, Youcha S, Lasser R, et al. Lisdexamfetamine dimesylate for the treatment of attention deficit hyperactivity disorder in adults with a history of depression or history of substance use disorder. *Innovations in Clinical Neuroscience.* 2011;8:28
59. Konstenius M, Jayaram-Lindstrom N, Beck O, et al. Sustained release methylphenidate for the treatment of ADHD in amphetamine abusers: a pilot study. *Drug Alcohol Depend.* 2010;108:130-3
60. Konstenius M, Jayaram-Lindstrom N, Guterstam J, et al. Methylphenidate for attention deficit hyperactivity disorder and drug relapse in criminal offenders with substance dependence: A 24-week randomized placebo-controlled trial. *Addiction* 2014;109:440-9
61. Kooij JJ, Burger H, Boonstra AM, et al. Efficacy and safety of methylphenidate in 45 adults with attention-deficit/hyperactivity disorder. A randomized placebo-controlled double-blind cross-over trial. *Psychol Med.* 2004;34:973-82
62. Kooij JJ, Rosler M, Philipsen A, et al. Predictors and impact of non-adherence in adults with attention-deficit/hyperactivity disorder receiving OROS methylphenidate: results from a randomized, placebo-controlled trial. *BMC psychiatry.* 2013;13:36
63. Kuperman S, Perry PJ, Gaffney GR, et al. Bupropion SR vs. methylphenidate vs. placebo for attention deficit hyperactivity disorder in adults. *Annals of Clinical Psychiatry.* 2001;13:129
64. Lee SI, Song DH, Shin DW, et al. Efficacy and safety of atomoxetine hydrochloride in Korean adults with attention-deficit hyperactivity disorder. *Asia-Pacific psychiatry: Official Journal of the Pacific Rim College of Psychiatrists* 2014; 6:386
65. Leuchter AF, McGough JJ, Korb AS, et al. Neurophysiologic predictors of response to atomoxetine in young adults with attention deficit hyperactivity disorder: a pilot project. *Journal of Psychiatric Research.* 2014;54:11
66. Levin ED, Conners CK, Silva D, et al. Effects of chronic nicotine and methylphenidate in adults with attention deficit/hyperactivity disorder. *Exp Clin Psychopharmacol.* 2001;9:83-90
67. Levin FR, Choi CJ, Pavlicova M, Mariani JJ, Mahony A, Brooks DJ, et al. How treatment improvement in ADHD and cocaine dependence are related to one another: A secondary analysis. Drug & Alcohol Dependence. 2018;188:135-40. doi: https://dx.doi.org/10.1016/j.drugalcdep.2018.03.043.
68. Levin FR, Evans SM, Brooks DJ, et al. Treatment of cocaine dependent treatment seekers with adult ADHD: double-blind comparison of methylphenidate and placebo. *Drug Alcohol Depend.* 2007;87:20-9
69. Levin FR, Evans SM, Brooks DJ, et al. Treatment of methadone-maintained patients with adult ADHD: double-blind comparison of methylphenidate, bupropion and placebo. *Drug & Alcohol Dependence.* 2006;81:137
70. Levine FR, Mariani JJ, Specker S, et al. Extended-release mixed amphetamine salts vs placebo for comorbid adult attention-deficit/hyperactivity disorder and cocaine use disorder a randomized clinical trial. *JAMA Psychiatry* 2015;72:593-602.
71. Lin HY, Gau SS. Atomoxetine treatment strengthens an anti-correlated relationship between functional brain networks in medication-naive Adults with Attention-Deficit Hyperactivity Disorder: A Randomized Double-Blind Placebo-Controlled Clinical Trial. *Int J Neuropsychopharmacol* 2015;19(3):1-15.
72. Mamey M. Using parallel latent growth curve models to better understand co-morbidity in two randomized clinical trials: Smoking with co-morbid ADHD and co-morbid SUD [Dissertation Clinical Trial; Empirical Study; Quantitative Study]: Washington State University; 2017.
73. Martin PT, Corcoran M, Zhang P, et al. Randomized, double-blind, placebo-controlled, crossover study of the effects of lisdexamfetamine dimesylate and mixed amphetamine salts on cognition throughout the day in adults with attention-deficit/hyperactivity disorder. *Clinical Drug Investigation.* 2014;34:147
74. Matochik JA, Liebenauer LL, King AC, et al. Cerebral glucose metabolism in adults with attention deficit hyperactivity disorder after chronic stimulant treatment. *Am J Psychiatry.* 1994;151:658-64
75. Mattes JA, Boswell L, Oliver H. Methylphenidate effects on symptoms of attention deficit disorder in adults. *Arch Gen Psychiatry.* 1984;41:1059-63
76. Mattingly GW, Weisler RH, Young J, et al. Clinical response and symptomatic remission in short- and long-term trials of lisdexamfetamine dimesylate in adults with attention-deficit/hyperactivity disorder. *BMC psychiatry.* 2013;13:39
77. McRae-Clark AL, Carter RE, Kileen TK, et al. A placebo-controlled trial of atomoxetine in marijuana-dependent individuals with attention deficit hyperactivity disorder. *The American Journal on Addictions.* 2010;19:481-9
78. Medori R, Ramos-Quiroga JA, Casas M, et al. A randomized, placebo-controlled trial of three fixed dosages of prolonged-release OROS methylphenidate in adults with attention-deficit/hyperactivity disorder. *Biol Psychiatry.* 2008;63:981-9
79. Michelson D, Adler L, Spencer T, et al. Atomoxetine in adults with ADHD: two randomized, placebo-controlled studies. *Biol Psychiatry.* 2003;53:112-20
80. Ni HC, Hwang Gu SL, Lin HY, et al. Atomoxetine could improve intra-individual variability in drug-naive adults with attention-deficit/hyperactivity disorder comparabl with methylphenidate: A head-to-head randomized clinical trial. *J Psychopharmacol* 2016;30(5):459-67.
81. Ni HC, Lin YJ, Gau SSF, Huang HC, Yang LK. An Open-Label, Randomized Trial of Methylphenidate and Atomoxetine Treatment in Adults With ADHD. Journal of attention disorders. 2017;21(1):27-39. doi: http://dx.doi.org/10.1177/1087054713476549.
82. Ni HC, Shang CY, Gau SS, et al. A head-to-head randomized clinical trial of methylphenidate and atomoxetine treatment for executive function in adults with attention-deficit hyperactivity disorder. *Int J Neuropsychopharmcol.* 2013;16:1959
83. Nunes EV, Covey LS, Brigham G, et al. Treating nicotine dependence by targeting attention-deficit/ hyperactivity disorder (ADHD) with OROS methylphenidate: the role of baseline ADHD severity and treatment response. *Journal of Clinical Psychiatry.* 2013; 74:983
84. Paterson R, Douglas C, Hallmayer J, Hagan M, Krupenia Z. A randomised, double-blind, placebo-controlled trial of dexamphetamine in adults with attention deficit hyperactivity disorder. Aust N Z J Psychiatry. 1999;33:494-502.
85. Philipsen A, Graf E, Jans T, et al. A randomized controlled multicenter trial on the multimodal treatment of adult attention-deficit hyperactivity disorder: enrollment and characteristics of the study sample. *Attention deficit and hyperactivity disorders.* 2014;6:35
86. Philipsen A, Graf E, Tebartz van EL, et al. Evaluation of the efficacy and effectiveness of a structured disorder tailored psychotherapy in ADHD in adults: study protocol of a randomized controlled multicentre trial. *Attention deficit and hyperactivity disorders.* 2010;2:203
87. Philipsen A, Jans T, Graf E, et al. Effects of group psychotherapy, individual counseling, methylphenidate, and placebo in the treatment of adult attention-deficit/hyperactivity disorder: A randomized clinical trial. *JAMA Psychiatry.* 2015;72(12):1199-1210.
88. Reimherr FW, Hedges DW, Strong RE, et al. Bupropion SR in adults with ADHD: a short-term, placebo-controlled trial. *Neuropsychiatric Disease & Treatment.* 2005;1:245
89. Reimherr FW, Williams ED, Strong RE, et al. A double-blind, placebo-controlled, crossover study of osmotic release oral system methylphenidate in adults with ADHD with assessment of oppositional and emotional dimensions of the disorder. *J Clin Psychiatry* 2007;68:93-101
90. Retz W, Rosler M, Ose C, et al. Multiscale assessment of treatment efficacy in adults with ADHD: a randomized placebo-controlled, multi-centre study with extended-release methylphenidate. *World J Biol Psychiatry.* 2012;13:48
91. Robison RJR. Personality disorders in ADHD Part 2: The effect of symptoms of personality disorder on response to treatment with OROS methylphenidate in adults with ADHD. *Annals of Clinical Psychiatry.* 2010;22:94
92. Rosler M, Fischer R, Ammer R, et al. A randomised, placebo-controlled, 24-week, study of low-dose extended-release methylphenidate in adults with attention-deficit/hyperactivity disorder. *Eur Arch Psychiatry Clin Neurosci.* 2009;259:120-9
93. Rosler M, Ginsberg Y, Arngrim T, et al. Correlation of symptomatic improvements with functional improvements and patient-reported outcomes in adults with attention-deficit/hyperactivity disorder treated with OROS methylphenidate. *World J Biol Psychiatry.* 2013;14:282
94. Rosler M, Retz W, Fischer R, et al. Twenty-four-week treatment with extended release methylphenidate improves emotional symptoms in adult ADHD. *World Journal of Biological Psychiatry.* 2010;11:709-718
95. Sobanski E, Sabljic D, Alm B, et al. A randomized, waiting list-controlled 12-week trial of atomoxetine in adults with ADHD. *Pharmacopsychiatry.* 2012;45:100
96. Sobanski E, Sabljic D, Alm B, et al. Driving performance in adults with ADHD: results from a randomized, waiting list controlled trial with atomoxetine. *European Psychiatry: the Journal of the Association of European Psychiatrists.* 2013;28:379
97. Spencer T, Biederman J, Wilens T, et al. A large, double-blind, randomized clinical trial of methylphenidate in the treatment of adults with attention-deficit/hyperactivity disorder. *Biol Psychiatry.* 2005;57:456-63
98. Spencer T, Biederman J, Wilens T, et al. Effectiveness and tolerability of tomoxetine in adults with attention deficit hyperactivity disorder. *Am J Psychiatry.* 1998;155:693-5
99. Spencer T, Biederman J, Wilens T, et al. Efficacy of a mixed amphetamine salts compound in adults with attention-deficit/hyperactivity disorder. *Arch Gen Psychiatry.* 2001;58:775-82
100. Spencer T, Wilens T, Biederman J, et al. A double-blind, crossover comparison of methylphenidate and placebo in adults with childhood-onset attention-deficit hyperactivity disorder. *Arch Gen Psychiatry.* 1995;52:434-43
101. Spencer TJ, Adler LA, McGough JJ, et al. Efficacy and safety of dexmethylphenidate extended-release capsules in adults with attention-deficit/hyperactivity disorder. *Biol Psychiatry.* 2007;61:1380-7
102. Spencer TJ, Adler LA, Weisler RH, et al. Triple-bead mixed amphetamine salts (SPD465), a novel, enhanced extended-release amphetamine formulation for the treatment of adults with ADHD: a randomized, double-blind, multicenter, placebo-controlled study. *J Clin Psychiatry* 2008;69:1437-48
103. Spencer TJL. Attention-deficit/hyperactivity disorder-specific quality of life with triple-bead mixed amphetamine salts (SPD465) in adults: Results of a randomized, double-blind, placebo-controlled study. *Journal of Clinical Psychiatry.* 2008;69:1766
104. Sutherland SM, Adler LA, Chen C, et al. An 8-week, randomized controlled trial of atomoxetine, atomoxetine plus buspirone, or placebo in adults with ADHD. *Journal of Clinical Psychiatry.* 2012;73:445
105. Takahashi N, Koh T, Tominaga Y, et al. A randomized, double-blind, placebo-controlled, parallel-group study to evaluate the efficacy and safety of osmotic-controlled release oral delivery system methylphenidate HCl in adults with attention-deficit/hyperactivity disorder in Japan. *World J Biol Psychiatry.* 2014;15:488
106. Taylor FB, Russo J. Comparing guanfacine and dextroamphetamine for the treatment of adult attention-deficit/hyperactivity disorder. *J Clin Psychopharmacol.* 2001;21:223-8
107. Taylor FB, Russo J. Efficacy of modafinil compared to dextroamphetamine for the treatment of attention deficit hyperactivity disorder in adults. *J Child Adolesc Psychopharmacol.* 2000;10:311-20
108. Tenenbaum S, Paull JC, Sparrow EP, et al. An experimental comparison of Pycnogenol and methylphenidate in adults with Attention-Deficit/Hyperactivity Disorder (ADHD). *J Atten Disord.* 2002;6:49-60
109. Turner DC, Blackwell AD, Dowson JH, et al. Neurocognitive effects of methylphenidate in adult attention-deficit/hyperactivity disorder. *Psychopharmacology (Berl).* 2005;178:286-95
110. Turner DC, Clark L, Dowson J, et al. Modafinil improves cognition and response inhibition in adult attention-deficit/hyperactivity disorder. *Biol Psychiatry.* 2004;55:1031-40
111. Verster J.C., Roth T. Methylphenidate significantly reduces lapses of attention during on-road highway driving in patients with ADHD. *J ClinPsychopharmacology.* 2014; 34:633
112. Verster JC, Bekker EM, de Roos M, et al. Methylphenidate significantly improves driving performance of adults with attention-deficit hyperactivity disorder: a randomized crossover trial. *J Psychopharmacol.* 2008;22:230-7
113. Weisler RH, Biederman J, Spencer TJ, et al. Mixed amphetamine salts extended-release in the treatment of adult ADHD: a randomized, controlled trial. *CNS Spectr.* 2006;11:625-39
114. Weisler RH, Pandina GJ, Daly EJ, et al. Randomized clinical study of a histamine H3 receptor antagonist for the treatment of adults with attention-deficit hyperactivity disorder. *CNS Drugs.* 2012;26:421
115. Weiss M, Hechtman L, Adhd Research Group Adult. A randomized double-blind trial of paroxetine and/or dextroamphetamine and problem-focused therapy for attention-deficit/hyperactivity disorder in adults. *J Clin Psychiatry.* 2006;67:611-9
116. Wender PH, Reimherr FW, Wood D, et al. A controlled study of methylphenidate in the treatment of attention deficit disorder, residual type, in adults. *Am J Psychiatry.* 1985;142:547-52
117. Wender PHR. A One Year Trial of Methylphenidate in the Treatment of ADHD. *Journal of attention disorders.* 2011;15:36
118. Westover AN, Nakonezny PA, Winhusen T, et al. Risk of methylphenidate-induced prehypertension in normotensive adult smokers with attention deficit hyperactivity disorder. *Journal of Clinical Hypertension.* 2013;15:124
119. Wietecha L, Young J, Ruff D, et al. Atomoxetine once daily for 24 weeks in adults with attention-deficit/hyperactivity disorder (ADHD): impact of treatment on family functioning. *Clinical Neuropharmacology.* 2012;35:125
120. Wigal T, Brams M, Gasior M, et al. Effect size of lisdexamfetamine dimesylate in adults with attention-deficit/hyperactivity disorder. *Postgraduate Medicine.* 2011;123:169
121. Wigal T, Brams M, Gasior M, et al. Randomized, double-blind, placebo-controlled, crossover study of the efficacy and safety of lisdexamfetamine dimesylate in adults with attention-deficit/hyperactivity disorder: novel findings using a simulated adult workplace environment design. *Behavioral & Brain Functions* 2010;6:34
122. Wilens TE, Adler LA, Tanaka Y, et al. Correlates of alcohol use in adults with ADHD and comorbid alcohol use disorders: exploratory analysis of a placebo-controlled trial of atomoxetine. *Current Medical Research & Opinion.* 2011;27:2309
123. Wilens TE, Adler LA, Weiss MD, et al. Atomoxetine treatment of adults with ADHD and comorbid alcohol use disorders. *Drug Alcohol Depend.* 2008;96:145-54
124. Wilens TE, Haight BR, Horrigan JP, et al. Bupropion XL in adults with attention-deficit/hyperactivity disorder: a randomized, placebo-controlled study. *Biological Psychiatry.* 2005;57:793
125. Wilens TE, Spencer TJ, Biederman J, et al. A controlled clinical trial of bupropion for attention deficit hyperactivity disorder in adults. *American Journal of Psychiatry.* 2001;158:282
126. Winhusen TMS. Impact of Attention-Deficit/Hyperactivity Disorder (ADHD) treatment on smoking cessation intervention in ADHD smokers: A randomized, double-blind, placebo-controlled trial. *Journal of Clinical Psychiatry.* 2010;71:1680
127. Young JL, Sarkis E, Qiao M, et al. Once-daily treatment with atomoxetine in adults with attention-deficit/hyperactivity disorder: a 24-week, randomized, double-blind, placebo-controlled trial. *Clinical Neuropharmacology.* 2011;34:51
128. Weisler R, Ginsberg L, Dirks B, Deas P, Adeyi B, Adler LA. Treatment With Lisdexamfetamine Dimesylate Improves Self- and Informant-Rated Executive Function Behaviors and Clinician- and Informant-Rated ADHD Symptoms in Adults: Data From a Randomized, Double-Blind, Placebo-Controlled Study. Journal of attention disorders. 2017;21(14):1198-207.
129. Weisler RH, Greenbaum M, Arnold V, Yu M, Yan B, Jaffee M, et al. Efficacy and Safety of SHP465 Mixed Amphetamine Salts in the Treatment of Attention-Deficit/Hyperactivity Disorder in Adults: results of a Randomized, Double-Blind, Placebo-Controlled, Forced-Dose Clinical Study. CNS drugs. 2017;31(8):685‐97.
130. Wigal T, Brams M, Frick G, Yan B, Madhoo M. A randomized, double-blind study of SHP465 mixed amphetamine salts extended-release in adults with ADHD using a simulated adult workplace design. Postgrad Med. 2018;130(5):481-93.
131. Wigal T, Childress A, Frick G, Yan B, Wigal S, Madhoo M. Effects of SHP465 mixed amphetamine salts in adults with ADHD in a simulated adult workplace environment. Postgrad Med. 2017:1‐11.

# **Appendix E. Detailed inclusion and exclusion criteria**

| **Study name** | **Inclusion criteria** | **Exclusion criteria** | **Excluded psychiatric comorbidities** | **Included only treatment naïve participants?** | **Washout period** |
| --- | --- | --- | --- | --- | --- |
| Paterson 1999, p. 494 [55] | “Patients were eligible for inclusion in the trial if they reported the presence of at least four inattentive and/or five hyperactive symptoms during the previous 6 months.” | “Patients were excluded from the study on the grounds of either having an insufficient ADHD score, or comorbidity for other major psychiatric disorders, including a history of current substance abuse...Patients were also screened for organic disorders that would contraindicate the use of dexamphetamine. Finally, all patients eligible for the trial had a sample of urine tested to screen for illicit substance abuse.” | “other major psychiatric disorders”, including a history of current substance abuse” | NR | NR |
| Butterfield 2016, p. 136 [32] | “All participants had a current ADHD diagnosis derived from the diagnostic criteria for adult ADHD (inattentive, hyperactive/impulsive, or combined subtypes), as specified in the Diagnostic and Statistical Manual of Mental Disorders, Fourth Edition – Text Revision (APA, 2000)… Participants were required to be on a current treatment regimen of stimulant  medications at the time of the screening interview, and all who were selected had reported a lengthy pharmaceutical treatment history for ADHD (years to decades)… Each participant presented at Visit 1 with a sub-optimal response to their treatment regimen. Sub-optimal response was defined by the participant's dissatisfaction with his/her clinical progress, a Visit 1 baseline score of greater than or equal to 28 using the ADHD – RS, or a CGI – S score greater than or equal to 4 at Visits 1… In addition to these hypothesis-specific inclusion criteria, more general inclusion criteria were also specified. Participants, in the opinion of the investigator, must have been able to understand and comply with protocol requirements, including assessments, prescribed dosage regimens, and discontinuation of concomitant medications… They demonstrated  a typical level of intellectual functioning without evidence  of significant general intellectual deficit, and they were able to swallow intact tablets. Finally, women were required to have a negative urine pregnancy test at Visit 1, and they agreed to use medically accepted means of contraception during the study.” | “Participants with severe comorbid psychiatric diagnoses (e.g., Axis I disorders such as mood disorders, anxiety disorders, posttraumatic  stress disorder, obsessive compulsive disorder, etc.) were excluded, as were participants with a history of psychosis,  pervasive developmental disorders, severe Axis II disorders or severe substance dependence… Participants  were also excluded if they had a chronic or an acute medical condition or illness that could have been negatively affected by the study medication. Those with a history of hypothyroidism, hypertension, or a resting systolic blood pressure  4140 mmHg or diastolic blood pressure 490 mmHg were ineligible. Participants who were directly affiliated with the study team, and those who were receiving treatment with an unregulated medication or had participated in a clinical trial within 30 days prior to screening, were also excluded. Individuals could not participate if they weighed less than 30 kg or more than 120 kg at the time of informed consent.” “All investigational medications, tricyclic antidepressants, STRATTERAs, antipsychotics, neuroleptics, psychostimulants (other than entry defined use of YVANSE™, CONCERTAs, RITALIN™, FOCALIN™, and/or ADDERALL™) were prohibited. These included sympathomimetics, appetite suppressants, modafinil, cough/cold preparations containing stimulants, other medications containing amphetamine, clonidine and guanfacine, monoamine oxidase inhibitors, anticonvulsant medications, any antibiotics with a CNS effect were prohibited.” | Severe comorbid psychiatric diagnoses (e.g., Axis I disorders such as mood disorders, anxiety disorders, posttraumatic  stress disorder, obsessive compulsive disorder, etc.), history of psychosis,  pervasive developmental disorders, severe Axis II disorders or  severe substance dependence | No | “A screening period of up to 30 days was used to determine the participants' eligibility to participate in the study and to engage in the appropriate washout of any excluded medications” |
| Biederman 2012, p. 484 [27] | “Subjects were both male and female outpatients, 18-26 years of age, who met full DSM-IV criteria for ADHD based on a clinical evaluation supplemented by structured diagnostic interview. Subjects had an onset of symptoms in childhood, a persistence of impairing symptoms into adulthood, and did not have pharmacological treatment for ADHD in the past month.” | “We excluded potential subjects if they had any other clinically significant psychiatric or medical conditions, including clinically significant laboratory or ECG values, hypertension, pre-existing structural cardiac abnormalities, or a known hypersensitivity to LDX or any amphetamine compounds. We also excluded individuals who used psychotropics or any medication in the past month with clinically significant central nervous system effects. Individuals with an IQ < 80, or a history of substance dependence or abuse within six months preceding the study were also excluded, as were pregnant or nursing females and individuals who had never held a valid driver’s license.” | “Clinically significant psychiatric conditions” | No: “Out of seventy-five subjects enrolled in this study — and of 61 completers — 30 subjects had a history of prior ADHD medication treatment” | “...did not have pharmacological treatment for ADHD in the past month” |
| Sutherland 2012, p. 445 [65] | “Adults aged 18-60 years who met the Diagnostic and Statistical Manual of Mental Disorders, Fourth Edition, Text Revision (DSM-IV-TR) criteria for ADHD, via the Adult ADHD clinician Diagnostic Scale version 1.2 and scored >=24 on the adult ADHD Investigator Symptom Rating Scale (AISRS) were eligible to participate.” | “Individuals were excluded if they had a lifetime or current history of psychosis, bipolar disorder, mental retardation or learning disability; had current anxiety or depressive disorders; had substance abuse or dependence within 3 months of screening or positive urine screen or drugs of abuse at screening; used atomoxetine, buspirone, or a monoamine oxidase inhibitor within 2 weeks prior to screening; had seizure disorder, urinary retention, narrow-angle glaucoma, or cardiac conduction defects; had any current general medical conditions considered clinically significant as judged by the investigator; or were poor metabolizers of cytochrome P450 2D6 (CYP2D6). Use of substances with psychoactive properties and potent CYP3A4 or CYP2D6 inducers or inhibitors was prohibited.” | Lifetime or current history of psychosis, bipolar disorder, mental retardation or learning disability; current anxiety or depressive disorders | No | NR |
| Adler 2009, p. 212 [22] | “Adult patients, 18–65-years old, meeting the DSM-IV-TR diagnoses for both ADHD and social anxiety disorder, were enrolled. The diagnostic criteria for ADHD were assessed with the Conners’ Adult ADHD Diagnostic Interview for DSM-IV and for social anxiety disorder by the Structured Clinical Interview for DSM-IVTR Axis I Disorders-Research Version. Additionally, patients had an LSAS Total score of at least 50 at Visit 1, no more than a 30% decrease in LSAS Total score at Visit 2, and a Clinical Global Impression-Overall-Severity (CGI-O-S) score of 4 or greater at Visits 1 and 2. Concomitant Axis I diagnoses (current or lifetime)-specific phobias, Generalized Anxiety Disorder (GAD), and dysthymia were allowed. Current diagnosis of major depressive disorder was allowed only if diagnosed more than 6 months before Visit 1” | “Exclusionary criteria included current or lifetime diagnosis of obsessive–compulsive disorder, bipolar affective disorder, psychosis, factitious disorder, or somatoform disorders, and/or current diagnosis of panic disorder, posttraumatic stress disorder, or an eating disorder within the year preceding Visit 1. Current diagnosis of alcohol, drugs of abuse, or prescription medication abuse meeting DSM-IV-TR criteria were also excluded.” | Current or lifetime diagnosis of obsessive–compulsive disorder, bipolar affective disorder, psychosis, factitious disorder, or somatoform disorders, and/or current diagnosis of panic disorder, posttraumatic stress disorder | No | 2-week medication-free evaluation period. “ Patients taking a stimulant for ADHD were required to be stimulant-free for 24 hr before the second screening visit“ |
| Weiss 2006, p. 611 [70] | “Participants were patients, 18 to 66 years of age, recruited from clinical outpatient psychiatric services, who met DSM-IV diagnostic criteria for ADHD” | "Adults with eating disorders, substance abuse disorders, organic brain syndrome, neurologic disease, psychosis, and active suicide risk were excluded. Other comorbid conditions were permitted if in the opinion of the investigator they did not require treatment with psychotropic medications other than those provided in this protocol. | Eating disorders, substance abuse disorders, organic brain syndrome, neurologic disease, psychosis, and active suicide risk | NR | “After a full baseline assessment, eligible participants entered a 1-week single blind placebo washout.” |
| Biehl 2016, p. 1 [31] | “patients with ADHD were recruited from the ADHD outpatient clinic... Diagnoses were made by an experienced psychiatrist according to DSM-IV-TR (2000). Patients had to be medication naïve or without medication for at least 3 months prior to testing with no obvious comorbid disorders to be approached for participation” | NR | NR | No | NR |
| Schrantee 2016, p. 955 (Bottelier 2014) [60] | “...stimulant treatment–naive men (23-40 years old) diagnosed as having ADHD” based on “strict criteria for ADHD according to the DSM-IV16 and were diagnosed by an experienced psychiatrist (M.A.B.), which was confirmed with the Diagnostic Interview Schedule for Children (authorized Dutch translation)17 and the Diagnostic Interview for ADHD in Adults.” | “Patients with comorbid Axis I psychiatric disorders requiring treatment with medication at study entry, a history of major neurological or medical illness, or a history of clinical treatment with drugs influencing the DAergic system (for adults before age 23 years), such as stimulants, neuroleptics, antipsychotics, and dopamine 2 and 3 (D2 and D3) agonists, were excluded.” | Comorbid Axis I psychiatric disorders requiring treatment with medication at study entry, history of major neurological or medical illness | Participants were naive to stimulants but no information provided about non-stimulant use | NR |
| Fan 2017, p. 4850 [35] | Twenty-four drug-naive adults…  who had current and childhood ADHD diagnosis according to the DSM-IV diagnostic criteria based on clinical assessment by the corresponding  author (SSG)... native Mandarin-Chinese speakers, had standard scores of the IQ greater than 80 as assessed  by the Wechsler Adult Intelligence Scale, third version [Wechsler, 1997], with normal hearing and normal or  corrected-to-normal vision” | “Participants, who had any systemic medical illness, a history of other psychiatric disorders or had been treated with any psychotropic agent,  including medications for ADHD were excluded” | History of other psychiatric disorders or had been treated with any psychotropic agent,  including medications for ADHD | Yes | NA |
| Goodman 2017, p. 105 [38] | “Eligible participants were adults aged 18 to 65 years with a diagnosis of ADHD, as defined by the Diagnostic and Statistical Manual of Mental Disorders, Fourth Edition, and as evaluated at baseline with the adult ADHD Clinical Diagnostic Scale (ACDS), version 1.2, and the Mini-  International Neuropsychiatric Interview.24 Prospective subjects had an adult ADHD Investigator Symptom Rating Scale (AISRS) score > 24 at the screening/baseline visit. Those with mild depression according to the Hamilton Depression Rating Scale (HDRS; HDRS score < 18) or  mild anxiety according to the Hamilton Anxiety Rating Scale (HARS; HARS score < 21) were eligible for study participation.” | “Subjects excluded were those with a history of diagnosis of substance or alcohol dependence or admission/hospitalization for rehabilitation for dependence, moderate  or severe anxiety (HARS score ≥ 21), moderate or severe depression (HDRS score ≥ 18), and a history of stimulants or atomoxetine use within 5 years or other ADHD medications within 30 days and those for whom, in the investigator’s opinion, methylphenidate posed an unacceptable risk through a potential drug interaction or a concurrent medical, neurologic, or psychiatric illness.” | Moderate  or severe anxiety (HARS score ≥ 21), moderate or severe depression (HDRS score ≥ 18); concurrent medical, neurologic, or psychiatric illness | NR | NR |
| Goto 2017, p. 100 [39] | “Patients were adults ≥18 years of age who met the *Diagnostic and Statistical Manual of Mental Disorders* (4^th^ ed., text rev.; *DSM-IV-TR*; American Psychiatric Association, 2000) criteria for current ADHD and had a historical diagnosis of ADHD during childhood, as assessed by the Conners’ Adult ADHD Diagnostic Interview (Conners, Erhardt, & Sparrow, 1999). Patients were required to meet the following additional criteria: scored ≥2 on at least 6 items of either the inattentive or hyperactive/impulsive subscale scores at Visits 1 and 2 on the Conners’ Adult ADHD Rating Scale–Investigator Rated: Screening Version (CAARS-Inv: SV); and a CGI-ADHD-S score ≥4 at Visits 1 and 2.” | “Major exclusion criteria included a history of bipolar disorder or schizophrenia, depressive disorder  with a score ≥12 on the 17-item Hamilton Depression Rating Scale, or any current anxiety disorder” | History of bipolar disorder or schizophrenia, depressive disorder  with a score ≥12 on the 17-item Hamilton Depression  Rating Scale, or any current anxiety disorder | About 20% had prior stimulant exposure | “Study period I was a screening and  wash-out period lasting 3 to 28 days” |
| Philipsen 2010, p. 203 [56](Philipsen 2014, Philipsen 2015) | “Age 18–60 years, inclusive; Diagnosis of ADHD according to DSM-IV criteria Chronic course of ADHD symptoms from childhood to adulthood, and Wender-Utah Rating Scale short version C 30 No pathological abnormality detected on physical examination, routine blood testing (blood count, renal, hepatic, and thyroid function), ECG, and EEG” | “IQ <85 (Multiple-Choice Vocabulary Test <17); schizophrenia, bipolar disorder, borderline personality disorder, antisocial personality disorder, suicidal or self-injurious behavior, autism, motor tics, Tourette’s syndrome; Substance abuse/dependence within 6 months prior to screening (episodic abuse is not an exclusion criterion); positive drug screening; Neurological diseases, seizures, glaucoma, diabetes mellitus, hyperlipidemia, uncontrolled arterial hypertension, angina pectoris, tachycardia arrhythmia, arterial occlusive disease; Previous stroke; Current eating disorder (bulimia and anorexia), low weight (BMI<20, later protocol amendment BMI<19); Pregnancy (current or planned) or breast-feeding; no reliable contraception (Pearl Index >1%); Participation in another clinical trial (up to 3 months prior); Treatment with stimulants or ADHD-specific psychotherapy in the 6 months prior to study inclusion; Known MPH intolerance; Treatment with antidepressants (e.g., SSRI, TCA), NRI (e.g., atomoxetine), bupropion, neuroleptic medication, theophylline, amantadine, anticoagulants derived from coumarin, phenylbutazone, antacids, and alpha-adrenergic agonists (e.g., clonidine) in the 2 weeks prior to baseline assessment (T1); Treatment with fluoxetine, monoamine oxidase inhibitors in the 4 weeks prior to baseline assessment (T1); Refusal to comply with study requirements; Other psychotherapeutic or psychopharmacological treatment incompatible with the protocol” | Schizophrenia, bipolar disorder, borderline personality disorder, antisocial personality disorder, suicidal or self-injurious behavior, autism, motor tics, Tourette’s syndrome; | No; Known MPH intolerance excluded; patients with treatment with stimulants or ADHD-specific psychotherapy in the 6 months prior were excluded | NR |
| Hamedi 2014, p. 675 [40] | “Subjects were outpatients who were referred to a psychiatrist for psychiatric evaluation.”…”All subjects were between 20 and 60 years of age” | “Subjects with these conditions were excluded from the study: Any chronic medical condition such as cardiovascular disease, epilepsy and brain organic disease; Substance abuse or dependence during last 6 months; Pregnancy or breastfeeding . Mental retardation (IQ > 75); Unstable psychiatric state (e.g. suicide, aggression, psychosis) ; Any psychotropic medication usage currently; Any usage of methylphenidate, atomoxetine, amphetamine, and other ADHD related medication during last 3 months; Bipolar mood disorder” | Unstable psychiatric state (e.g. suicide, aggression, psychosis), bipolar mood disorder | No: “Any psychotropic medication usage currently . Any usage of methylphenidate, atomoxetine, amphetamine, and other ADHD related medication during last 3 months.” | No formal washout period; study only included only participants who had not used ADHD medications within the last 3 mo |
| Lee 2014, p. 386 [46] | “Patients were adults ≥18 years of age who met the Diagnostic and Statistical Manual of Mental Disorders, 4th Edition, Text Revision (DSM-IV-TR) (American Psychiatric Association, 2000) criteria for current ADHD and had a historical diagnosis of ADHD during childhood, as assessed by the Conners’ Adult ADHD Diagnostic Interview for DSM-IV”... “Patients were required to meet additional criteria, which included a score of ≥2 on ≥6 items of either the inattentive or hyperactive/ impulsive subscale scores at visits 1 and 2 on the Conners’ Adult ADHD Rating Scale-Investigator-rated: Screening Version (CAARS-Inv:SV); and a Clinical Global Impression-ADHD-Severity (CGIADHD-S) score of ≥4 at visits 1 and 2.” | “Key exclusion criteria included a history of bipolar disorder or schizophrenia, depressive disorder with ≥12 on the 17-item Hamilton Depression Rating Scale, and current anxiety disorders.” | History of bipolar disorder or schizophrenia, depressive disorder with ≥12 on the 17-item Hamilton Depression Rating Scale, current anxiety disorders | NR | “The overall study consisted of two study periods: a screening and washout period lasting for 3–28 days and a double-blind treatment period lasting up to 70 days” |
| Kollins 2014, p. 158 [42] | “To be eligible for the study, participants had to be between 18 and 50 years of age, express interest in quitting smoking, smoke at least 10 cigarettes/day, have an expired air CO level of at least 10 ppm, meet full Diagnostic and Statistical Manual of Mental Disorders (4th ed.; DSM-IV; American Psychiatric Association, 1994) criteria for ADHD, and have an estimated IQ score of ≥80.” | “Exclusionary criteria included the presence of any other psychiatric condition, use of illicit drugs (confirmed by urine drug screen), and for females of childbearing potential, pregnancy. Participants were also excluded if they were currently taking psychoactive medication, although those taking medication for ADHD (n = 4) were allowed to wash out of their medication for a period of 5 half-lives prior to the first postscreening visit.” | “any other psychiatric condition” | No | “Although those taking medication for ADHD (n = 4) were allowed to wash out of their medication for a period of 5 half-lives prior to the first postscreening visit” |
| Konstenius 2014, p. 440 [44] | “The study included men aged between 18 and 65 years, recruited from three medium-security prisons in Stockholm County, Sweden.”…” The study included participants who met the diagnostic criteria for ADHD according to the Diagnostic and Statistical Manual of Mental Disorders (DSM-IV) [21] and the DSM-IV diagnostic criteria for amphetamine dependence during the last 12 months prior to the current incarceration, and had used amphetamines on a minimum of 12 occasions during the last 12 weeks preceding the incarceration.” | “The study exclusion criteria were: (i) DSM-IV diagnosis of any other substance dependence except nicotine, currently or during the 12 months prior to incarceration, (ii) a major psychiatric disorder (e.g. schizophrenia, severe depression), (iii) current antipsychotic medication, (iv) current use of benzodiazepine, (v) traces of any of the following substances in urine: amphetamine, benzodiazepine, cannabis, cocaine, dextropropoxyphene and opiates, (vi) serious somatic disease (e.g. moderate to severe hypertension >150/95 mm Hg, hyperthyroidism) and (vii) known hypersensitivity to methylphenidate.” | Major psychiatric disorder (e.g. schizophrenia, severe depression) | No | NR |
| Takahashi 2014, p. 488 [66] | “Eligible patients were men and women between 18 and 64 years of age, who met the DSM-IV Text Revision (DSM-IV-TR) criteria for ADHD (American Psychiatric Association 2000) both at present and in childhood (onset of symptoms before the age of 7 years according to DSM-IV-TR criteria) based on Conners ’Adult ADHD Diagnostic Interview for DSM-IV (CAADID) Japanese version at screening.” | “Patients were excluded from the study if they were a non-responder to MPH and/or had a history of hypersensitivity or intolerance to MPH or had been treated with MPH or any other medications for ADHD within 4 weeks before the screening visit”…” Other exclusion criteria included diagnosis of bipolar I disorder, schizophrenia, schizoaffective disorder, severe obsessive compulsive disorder, pervasive developmental disorder (e.g., autistic disorder or Asperger’s disorder) or suicidality. Patients with confirmed cancer or other serious illnesses (e.g., hepatic or renal insufficiency or significant cardiac, gastrointestinal, psychiatric, or metabolic disturbances) were also excluded.” | Bipolar I disorder, schizophrenia, schizoaffective disorder, severe obsessive compulsive disorder, pervasive developmental disorder (e.g., autistic disorder or Asperger’s disorder) or suicidality | No | “The screening period also allowed for washout of prohibited medications in order to appropriately assess the safety and efficacy of OROS MPH. Up to 4 weeks before study drug administration were allowed for washout of medications used for the treatment of ADHD (e.g., MPH and atomoxetine HCl).” |
| Adler 2013, p. 694 [20] (Adler 2013,Weisler 2017, p.1198) | “Adults aged 18–55 years who met full DSM-IV-TR40 criteria for a primary diagnosis of ADHD were eligible. Participants were required to be in a close domicile relationship (eg, spouse or significant other) for ≥6 months prior to screening to ensure the availability of an informant who was willing to report on the participant’s behavior and symptoms. Additional inclusion criteria included a baseline BRIEF-A Global Executive Composite (GEC) T-score ≥ 65, indicating clinically significant executive function impairment at baseline, and a baseline total score ≥28 on the ADHD-RS-IV with adult prompts.” | “Adults with comorbid psychiatric conditions that were controlled with a prohibited medication or were uncontrolled and associated with significant symptoms, including severe Axis I or II disorders, were excluded from the study. Other key exclusion criteria included cardiovascular disease, which may increase vulnerability to the sympathomimetic effects of a psychostimulant; a history of moderate to severe hypertension; ADHD that was well controlled on current ADHD therapy; and a history of failure to respond to an adequate course of amphetamine therapy.” | Psychiatric conditions controlled with a prohibited medication or were uncontrolled and associated with significant symptoms, including severe Axis I or II disorders | NR | “Participants underwent a screening and washout period lasting up to 4 weeks” |
| Durell 2013, p. 45 [98] (Leuchter 2014, Adler 2014) | “Adults, aged 18 to 30 years, met DSM-IV, Text Revision (DSM-IV-TR) criteria for ADHD as determined by a clinical interview and assessed by the Adult ADHD Clinician Diagnostic Scale version 1.2. All participants also must have had a Clinical Global Impression-ADHD-Severity (CGI-S) score of 4 (moderate symptoms) or greater to be eligible for study participation. Participants with concomitant current or lifetime diagnoses of specific phobias, generalized anxiety disorder, or social anxiety disorder were allowed in the trial, as were participants with a history of dysthymia within 2 years of study screening” | “Potential participants were excluded from the trial if they had current major depression, panic disorder, posttraumatic stress disorder, an eating disorder, or substance abuse or dependence, as well as current or lifetime obsessive-compulsive disorder, bipolar disorder, or psychosis.” | Current major depression, panic disorder, posttraumatic stress disorder, an eating disorder, or substance abuse or dependence, as well as current or lifetime obsessive-compulsive disorder, bipolar disorder, or psychosis. | No | “Participants underwent a washout period if they had been taking medications excluded by the study protocol.” |
| Ni 2013, p. 1959 [54] (Ni 2016, Ni 2017) | “Those who met the diagnostic criteria of DSM-IV for ADHD and whose clinical diagnosis of ADHD was confirmed by the modified adult version of the ADHD supplement of the Chinese version of the Schedule for Affective Disorders and Schizophrenia–Epidemiological Version (K-SADS-E; Chang, 2012) for childhood and current diagnosis of ADHD would be enrolled…All participants were assessed by the Wechsler Adult Intelligence Scale – Revised to confirm their IQ >80.” | “Participants were excluded if they had any systemic medical illness, such as cardiovascular disease, a history of bipolar disorder, psychosis, major depression, substance use disorder, learning disability, pervasive developmental disorder or mental retardation. Participants who currently had depressive or anxiety symptoms or suicidal ideation or who had been treated with any psychotropic agent, including medications for ADHD, were also excluded.” | History of bipolar disorder, psychosis, major depression, substance use disorder, learning disability, pervasive developmental disorder or mental retardation; currently had depressive or anxiety symptoms or suicidal ideation or who had been treated with any psychotropic agent, including medications for ADHD | NR | NR |
| Ginsberg 2012, p. 68 [37] (Ginsberg 2012) | “To enter the trial, participants had to have confirmed ADHD in accordance with DSM-IV and to agree not to behave violently during the study. Participants with comorbid disorders such as autism-spectrum disorder, anxiety and depression could take part if they were considered to be stable at baseline. Previous drug elicited episodes of psychosis were not a cause for exclusion, other than chronic psychoses. Concurrent medication not interfering with methylphenidate was permitted for treating comorbid disorders, as long as doses were stable for at least 1 month at baseline. Medications interfering with methylphenidate had to be tapered off before the baseline visit took place.” | “Participants were excluded if they were known to be non- responsive or intolerant to methylphenidate, or intolerant to lactose. In addition, participants were excluded if they showed evidence of substance misuse up to 3 months before baseline, assessed in urine samples. Intellectual disability, epilepsy, glaucoma, uncontrolled hypertension, angina pectoris, cardiac arrhythmias, cardiac abnormality or a family history of serious cardiac illnesses were exclusion criteria, but hepatitis C without liver insufficiency did not preclude inclusion.” | Unstable comorbid disorders (eg autism-spectrum disorder, anxiety and depression) | No | “The screening visit took place up to 2 weeks before randomisation to enable medications excluded from the study to be tapered off prior to the baseline visit.” |
| Retz 2012, p. 48 [58] | “Subjects were outpatients with ADHD aged 18 years and older. For study inclusion the subject had to fulfill the DSM-IV criteria for ADHD (314.00 and 314.01). The diagnosis was established by clinical assessment and by use of a German standardized diagnostic instrument for psychiatric experts (ADHD-DC, R ö sler et al. 2004). A retrospective assessment of DSM-IV ADHD symptoms was made in the presence of an informant whenever possible. In addition, the German short version of the Wender Utah Rating scale (WURS, Wender 1995) was administered to all subjects in order to make sure that childhood ADHD symptoms were present by a retrospective self report of the patient. A cut-off score of at least 30 points served as an indicator of apparent childhood ADHD psychopathology (Retz-Junginger et al. 2002, 2003). Subjects with WURS-k scores < 30 were not included.” | “Individuals with low intelligence (IQ 85), dementia, schizophrenia, bipolar disorder, current major depression, acute anxiety disorders and other unstable psychiatric conditions were excluded, as were subjects with any serious medical illness. Also subjects with drug or alcohol dependence during the 6 months before screening, pregnant or nursing women, persons with a Body Mass Index <20 or body weight >=130 kg, and individuals treated with any psychopharmacological drug in addition to study medication were not included.” | Dementia, schizophrenia, bipolar disorder, current major depression, acute anxiety disorders and other unstable psychiatric conditions | No | “A wash-out period of at least 2 weeks was necessary for any psychopharmacological drug before study inclusion” |
| Sobanski 2012, p. 100 [61] (Sobanski 2013) | “Subjects were 18-50 years old and presented with a clinical diagnosis of ADHD according to DSM-IV criteria and a chronic course of ADHD from child- to adulthood. … Only patients with an IQ >85, no clinically relevant medical condition including ECG, laboratory values and physical examination and reliable contraception (Pearl Index < 1) were included” | “Main exclusion criteria for study participation were acute suicidality, total score in the Beck Depression Inventory >=19 points, affective disorders, schizophrenia, generalized anxiety and panic disorders, substance abuse within 6 months prior to screening, or antisocial personality or a history of schizophrenia or bipolar disorder as assessed with the SCID-I and II interviews. Other exclusion criteria were serious chronic medical illnesses, hyperthyroidism, myocardial infarction, or stroke within 6 months of screening, history of acute seizures, glaucoma, un controlled hypertension, pregnancy and breastfeeding, positive baseline drug screening and psychopharmacologic treatment within the last 2 weeks before baseline assessment.” | Acute suicidality, total score in the Beck Depression Inventory >=19 points, affective disorders, schizophrenia, generalized anxiety and panic disorders, substance abuse within 6 months prior to screening, or antisocial personality or a history of schizophrenia or bipolar disorder | NR | “Max. 4 weeks washout if needed” |
| Young 2011, p. 51 [75] (Wietecha 2012) | “Adults 18 years of age or older were required to meet DSM-IV-TR1 criteria for adult ADHD and have a historical diagnosis of ADHD during childhood, both of which were assessed by the Conners’ Adult ADHD Diagnostic Interview for DSM-IV. 9 Additionally, patients were required to have a Clinical Global Impressions YADHD-Severity (CGI-ADHD-S)10 score of 4 (moderate symptoms) or greater and meet family unit criteria (reciprocal relationship with a person of the opposite sex and living in the same defined household with at least 1 child between ages 6 and 17 years old).” | “Based on clinical history and the Structured Clinical Interview for DSM-IV-TR11 axis disorders, patients were excluded if diagnostic criteria were met for any history of bipolar or psychotic disorder, current major depression, anxiety disorder, or DSM-IV-TR criteria1 for substance abuse. Patients who were currently taking or had previously taken atomoxetine or were taking any psychotropic medication on a regular basis were excluded.” | Bipolar or psychotic disorder, current major depression, anxiety disorder | NR | “After an initial washout, screening, and entry period (study period I), patients were randomized…” (Fig 1: up to 38 days) |
| Konstenius 2010, p. 130 [43] | “Amphetamine dependent patients newly diagnosed with ADHD were referred to the project manager and their eligibility was ascertained via phone interviews…. The participants were required to fulfill the Diagnostic and Statistical Manual of Mental Disorders (DSM IV; APA, 1994) criteria for amphetamine dependence during the last 12-month period.” | “Exclusion criteria included: (1) current or past DSM IV diagnosis of any other  substance dependence except nicotine, (2) history of any major psychiatric disorder (e.g., schizophrenia and major depression) or any current psychiatric condition requiring medication, (3) use of any month, (4) current use of benzodiazepines, (5) traces of any illicit substance in the urine (amphetamine, cannabis, cocaine [bensoylecgonine], and opiates), (6) serious somatic disease (e.g., severe hypertension), (7) pregnant or lactating women, (8) known hypersensitivity for methylphenidate and (9) IQ < 70” | History of any major psychiatric disorder (e.g., schizophrenia and major depression) or any current psychiatric condition requiring medication | No | Yes: “The patients were required to stay abstinent from any psychoactive substance for a minimum of 4weeks prior to inclusion.” |
| Biederman 2010, p. 549 [30] (Biederman 2010) | “Subjects were outpatient adults with ADHD between 19 and 60 years of age. To be included, subjects had to satisfy full diagnostic criteria of ADHD based on DSM-IV with childhood onset and persistent symptoms based on clinical assessment and confirmed by structured diagnostic interview and an Adult ADHD Investigator Symptom Report Scale (AISRS) score of 24 or higher. Subjects treated for anxiety disorders and depression who were on a stable medication regimen for at least 3 months and who had a disorder-specific Clinical Global Impression (CGI)-Severity score of 3 or lower (mildly ill) were included.” | “We excluded potential subjects if they had clinically significant chronic medical conditions, abnormal baseline laboratory values, IQ of less than 80, delirium, dementia, or amnestic disorders, other clinically unstable psychiatric conditions (ie, bipolar disorder, psychosis, suicidality), drug or alcohol abuse or dependence within the 6 months preceding the study, or a previous adequate trial of MPH. We also excluded pregnant or breast-feeding females” | “clinically significant chronic medical conditions”; other clinically unstable psychiatric conditions (ie, bipolar disorder, psychosis, suicidality) | NR | NR |
| McRae-Clark 2010, p. 481 [52] | “... subjects had to be between 18 and 65 years of age and meet DSM-IV criteria for marijuana dependence. Participants also had to meet DSM-IV criteria for ADHD with the exception of the criterion that the age of onset of symptoms had to be prior to 7 years of age… Participants were therefore included if symptoms of ADHD were present prior to the age of 12.” | “Exclusion criteria included dependence on any other substance (with the exception of caffeine or nicotine); history of psychotic disorder; current major depression or eating disorder; current treatment with a psychoactive medication; major medical illnesses; cognitive impairment; and pregnancy, nursing, or inadequate birth control.” | History of psychotic disorder; current major depression or eating disorder; current treatment with a psychoactive medication | NR | NR |
| Winhusen 2010, p. 1680 [74] (Heffner 2012, Westover 2013, Nunes 2013, Berlin 2012, Covey 2011, Covey 2010, Covey 2011, Mamey 2017) | “Eligible participants were interested in quitting smoking and were between 18 and 55 years of age in good physical health as determined by a medical history, electrocardiogram, and vital signs… Participants were required to meet the Diagnostic and Statistical Manual of Mental Disorders, Fourth Edition (DSM-IV) criteria for ADHD as assessed by the Adult Clinical Diagnostic Scale, version 1.2; to have a DSM-IV ADHD Rating Scale (ADHD-RS) total score > 22; to smoke at least 10 cigarettes per day; to have a carbon monoxide (CO) level >= 8pm; to have smoked cigarettes for at least 3 months.” | “Candidates were excluded if they were a significant suicidal/homicidal risk; had used tobacco products other than cigarettes in the past week; had a positive urine screen for an illicit drug; or met DSM-IV criteria for current abuse or dependence of any psychoactive substance other than nicotine, current major depression, any current anxiety disorder except specific phobias, antisocial personality disorder, or a lifetime diagnosis of bipolar disorder or psychosis. Other exclusion criteria included a history of narrow angle glaucoma or seizure disorder, tics, or a family history of Tourette syndrome. Individuals were excluded if they had been treated for ADHD with psychomotor stimulants or had used smoking cessation counseling programs or medications within the last 30 days, if they were currently taking a medication that could adversely interact with OROS-MPH, had a known allergy to OROS-MPH, or if they had been non-responders to a reasonable course of MPH treatment. “ | Current major depression, any current anxiety disorder except specific phobias, antisocial personality disorder, or a lifetime diagnosis of bipolar disorder or psychosis. | No | NR |
| Adler 2009, p. 239 [25] | “The subjects were adults between 18 to 65 years of age (inclusive) with ADHD and weighed a minimum of 100 lb (45.4 kg). At subject screening, the diagnosis of ADHD inattentive, hyperactive/impulsive, or combined type as defined by the Diagnostic and Statistical Manual of Mental Disorders, Fourth Edition (DSM-IV) criteria was established through clinical evaluation by the investigator. The subject must have described a chronic course of ADHD symptoms from childhood to adulthood, have had an AISRS score of 24 or greater, and have had a global assessment of functioning score of between 41 and 60 (inclusive), indicating moderate or serious symptoms (according to DSM-IV criteria). Previous formal diagnosis of and/or treatment of ADHD were not required. Diagnosis of ADHD was confirmed by using the Adult ADHD Clinical Diagnostic Scale version 1.2 at baseline.” | “The patients who met the DSMIV criteria for depressive or anxiety disorders were excluded from the study, even if their HAM scores did not reach these cutoffs… Known nonresponders to methylphenidate were also excluded, as were subjects with a history of allergy to methylphenidate; any coexisting medical condition or taking any medication that was likely to interfere with the safe administration of methylphenidate; known or suspected structural cardiac abnormality as assessed by history, physical examination, or electrocardiogram (ECG); diagnosis or family history of Tourette syndrome or motor or verbal tics; or history of seizure disorder, uncontrolled hyperthyroidism, or hypothyroidism. Patients with comorbid psychiatric diagnosis per DSM-IV criteria of bipolar disorder, cyclothymic disorder, schizophrenia, pervasive developmental disorder, severe obsessive-compulsive disorders, or any other diagnosis that in the judgment of the investigator could have deemed the subject to be inappropriate for the study were excluded. Subjects with a history of drug or alcohol abuse within the past 6 months or with suicidal ideation or behavior during the past year were also excluded, as were subjects with a current or history of an eating disorder for the last 3 years.” | Depressive or anxiety disorders; comorbid psychiatric diagnosis per DSM-IV criteria of bipolar disorder, cyclothymic disorder, schizophrenia, pervasive developmental disorder, severe obsessive-compulsive disorder | No | “Subjects being treated for ADHD at screening were washed out from all ADHD medication for 7 to 14 days before beginning the study” |
| Adler 2009, p. 44 [23] (Brown 2011) | “Adults, aged 18 to 54 years, who met DSM-IV, Text Revision (DSM-IV-TR) criteria for adult ADHD as assessed by the Adult ADHD Clinician Diagnostic Scale version 1.2, had a Clinical Global Impressions YADHDY Severity of Illness (CGIADHD-S)12 score of 4 (moderate symptoms) or higher, had AISRS Symptom Checklist scores that did not change by more than 25% between visits 1 and 2, and had impairment due to ADHD symptoms in the home setting as indicated in the diagnostic interview were eligible to participate.” | “...patients were excluded from the study if they met diagnostic criteria for current major depression, a current anxiety disorder, any history of bipolar disorder, or any history of a psychotic disorder. Failure to respond to an adequate trial of treatment with ADHD stimulant medication, bupropion, or other nonstimulant medications (based upon the clinician’s judgment) was also exclusionary” | Current major depression, a current anxiety disorder, any history of bipolar disorder, or any history of a psychotic disorder | No | NR |
| Rosler 2009, p. 120 [59] (Rosler 2010) | “Subjects were outpatients with ADHD aged >18 years. For study inclusion the subject had to fulfil the DSM-IV criteria for ADHD.” | “Individuals with low intelligence (IQ < 85), schizophrenia, bipolar disorder, acute depressive episode, acute anxiety disorders and other unstable psychiatric conditions were excluded, as were subjects with any serious medical illness. Also subjects with evidence of drug or alcohol dependence during the preceding 6 months, pregnant or nursing women, persons who had participated in a previous drug trial in the last 30 days and individuals treated with any psychopharmacological drug in addition to study medication were not included” | Schizophrenia, bipolar disorder, acute depressive episode, acute anxiety disorders and other unstable psychiatric conditions | No | A washout period of at least 2 weeks was necessary for any psychopharmacological drug before study inclusion |
| Adler 2008, p. 720 [24] | “Participants had to meet the following criteria: be from ages 18 to 50 years old, meet criteria for current ADHD and a historical childhood diagnosis of ADHD according to the Diagnostic and Statistical Manual of Mental Disorders (4th ed., text revision; DSM-IV-TR; American Psychiatric Association, 2000), have a severity of illness of at least 4 (moderate) on the Clinician Global Impressions Severity Scale (CGI; Guy, 1976), and be employed for at least 20 hours per week for 6 months prior to study entry” | “Participants were excluded if they had a diagnosis of current major depression, an anxiety disorder (including generalized anxiety disorder, panic disorder, or social phobia), any current alcohol or substance abuse, or any lifetime history of bipolar illness or psychotic disorder. They were also excluded if they had any medical illness that would contraindicate the use of atomoxetine, current or past hypertension, and any history of organic brain disease or seizures other than febrile” | current major depression, an anxiety disorder (including generalized anxiety disorder, panic disorder, or social phobia), lifetime history of bipolar illness or psychotic disorder | No | “Participants were free of all psychotropic medications for at least 1 week prior to randomization”; included “a screening or washout phase (up to 28 days)” |
| Spencer 2008, p. 1437 [64] (Spencer 2008) | “...ages of 18 and 55 years, inclusive; meet the DSM-IV-TR criteria for a primary diagnosis of ADHD; have a satisfactory medical assessment with no clinically significant or relevant abnormalities; have a baseline ADHD Rating Scale-IV (ADHD-RS-IV) score >= 24…” | “Subjects were excluded if they had a [BMI] < 18.5, morbid obesity; comorbid psychiatric diagnosis with, in the opinion of the investigator, significant symptoms; seizure history, tic disorder, or diagnosis or family history of Tourette’s syndrome; current or chronic acute illness or unstable medical condition; mental retardation; known cardiac structural abnormality or any other cardiac condition that could affect cardiac performance; clinically significant electrocardiogram (ECG) or laboratory abnormalities at screening; used psychotropic medications that require more than a 28-day washout period; a history of controlled or uncontrolled hypertension or a resting, sitting systolic blood pressure > 139 mm Hg or diastolic blood pressure > 89 mm Hg at screening; allergy, intolerance, or nonresponse to methylphenidate or amphetamines; drug dependence or substance use disorder (excluding nicotine) within 6 months before screening; a positive urine drug test result at screening or baseline; participation in another investigational trial within 30 days of screening; pregnancy or lactation.” | Comorbid psychiatric diagnosis with “in the opinion of the investigator, significant symptoms” | No | “Those currently receiving stimulant therapy underwent at least a 7-day washout period before the baseline visit; those receiving bupropion, atomoxetine, or other medication for ADHD…were required to undergo a 28-day washout period before baseline evaluation.” |
| Wilens 2008, p. 145 [71] (Wilens 2011) | “…included adults ≥18 years of age meeting DSM-IV-TR (American Psychiatric Association, 2000) criteria for ADHD (any subtype), determined by clinical interview and confirmed by the Adult ADHD Clinician Diagnostic Scale (Adler and Cohen, 2004). ADHD symptom severity was ≥20 on the ADHD Investigator Symptom Rating Scale (AISRS) (Adler and Cohen, 2004). Subjects also met Diagnostic and Statistical Manual of Mental Disorders, Fourth Edition, Text Revision (DSM-IV-TR; American Psychiatric Association, 2000) criteria for alcohol use disorders (abuse or dependence)” | “Exclusion criteria included diagnosis of current bipolar disorder, major depressive disorder, or psychosis as determined by Structured Clinical Interview for DSM-IV-TR Axis I Disorders (First et al., 2002) or Hamilton Depression Rating Scale (HAM-D-17) (Hamilton, 1960, 1967) or Hamilton Anxiety Scale (HAM-A) (Hamilton, 1959) scores >18 at the evaluation visit. Subjects with significant cognitive impairment, judged by the investigator, were excluded. No other psychopharmacological treatments were permitted during the study, other than limited, intermittent hypnotic use | Current bipolar disorder, major depressive disorder, or psychosis as determined by Structured Clinical Interview for DSM-IV-TR Axis I Disorders or Hamilton Depression Rating Scale or Hamilton Anxiety Scale scores >18 | NR | NR |
| Levin 2007, p. 20 [48] | “Study inclusion required participants between the ages of 18–60 to meet DSM-IV criteria for cocaine dependence and persistent adult attention deficit hyperactivity disorder (ADHD).” | “Participants were excluded if they (1) met DSMIV criteria for current psychiatric disorders (other than ADHD or substance abuse) which required psychiatric intervention, (2) were physiologically dependent on opioids, sedatives or alcohol such that medical attention was required during periods of abstinence or significant reductions in use, (3) exhibited suicidal or homicidal behavior within the past 2 years, (4) were prescribed any psychotropic medication, (5) had an unstable medical condition that would make participation hazardous (i.e. uncontrolled diabetes), (6) had a known sensitivity to MPH, (7) were nursing and/or pregnant and (8) were unable to give full and informed consent.” | Current psychiatric disorders (other than ADHD or substance abuse) which required psychiatric intervention | No | Study “included a 1-week PBO lead-in phase” |
| Biederman 2006, p. 829 [29] | “Subjects were outpatient adults with ADHD aged 19 – 60 years. To be included, subjects had to satisfy full diagnostic criteria for DSM-IV ADHD on the basis of clinical assessment and confirmation by structured diagnostic interview… ““Subjects treated for anxiety disorders and depression who were receiving a stable medication regimen for at least 3 months and who had a disorder-specific Clinical Global Impression Scale (CGI)-Severity score of 3 or less (mildly ill) were not excluded. Thus, subjects receiving stable doses of non–monoamine oxidase inhibitor antidepressants or benzodiazepines for more than 3 months were eligible for this study…” | “We excluded potential subjects if they had clinically significant chronic medical conditions, abnormal baseline laboratory values, intelligence quotient less than 80, delirium, dementia, or amnesic disorders, other clinically unstable psychiatric conditions (i.e., bipolar disorder, psychosis, suicidality), drug or alcohol abuse or dependence within the 6 months preceding the study, or a previous adequate trial of MPH” | Other clinically unstable psychiatric conditions (i.e., bipolar disorder, psychosis, suicidality) | Previous MPH users were excluded but unclear if other ADHD medications were eligible | NR |
| Reimherr 2005, p. 245 [57] | “Outpatient subjects aged at least 18 years were required to meet not only DSM-IV but also the more restrictive Utah Criteria for ADHD in adults (Wender 1995) and to have a minimum score of 15 on the Wender-Reimherr Adult Attention Deficit Disorder Scale (WRAADDS). | “Any history of stimulant drug abuse or other recent substance abuse would exclude a patient from clinical trials involving stimulants… The Utah Criteria also exclude patients with the following characteristics or disorders: 1. bipolar and depressive mood disorders 2. signs and symptoms of schizophrenic spectrum disorders 3. borderline personality disorder 4. antisocial personality disorder… In addition to the exclusion factors in the Utah Criteria, eating disorders, seizure disorders, history of significant head injury, and situational stresses that were severe enough to confuse interpretation of outcome measures were exclusionary factors. Women who were pregnant or breast feeding, subjects under custody of the criminal justice system, subjects with a history of treatment with bupropion, and subjects at risk for suicide were excluded. Finally, individuals with other axis I disorders were excluded.” | Bipolar and depressive mood disorders; schizophrenic spectrum disorders; borderline personality disorder; antisocial personality disorder; other axis I disorders | Past bupropion was excluded; other ADHD drugs not mentioned | “1-week baseline phase with single-blind placebo administration” |
| Spencer 2005, p. 456 [62] | “Subjects had to satisfy full diagnostic criteria for DSM-IV ADHD based on clinical assessment and confirmed by structured diagnostic interview (Biederman et al 1993).” | “We excluded potential subjects if they had clinically significant chronic medical conditions; abnormal baseline laboratory values; IQ<80; delirium, dementia, or amnestic disorders; other clinically unstable psychiatric conditions (i.e., bipolar disorder, psychosis, suicidality); drug or alcohol abuse or dependence within the 6 months preceding the study; previous adequate trial of stimulant ( .5 mg/kg/day of MPH or equivalent); or current use of other psychotropics. We also excluded pregnant or nursing women” | “Other clinically unstable psychiatric conditions (i.e., bipolar disorder, psychosis, suicidality)”; delirium, dementia, or amnestic disorders | Previous use of MPH or other stimulant excluded | NR |
| Wilens 2005, p. 793 [72] | “Men and women aged 18 to 60 years were eligible for the study if they met criteria for a current diagnosis of ADHD (all types) as defined by the Diagnostic and Statistical Manual of Mental Disorders, 4th Edition (DSM-IV) (American Psychiatric Association 1994). Subjects were required to have met full DSM-IV criteria for a diagnosis of ADHD by age 7 (as determined by the Schedule for Affective Disorders and Schizophrenia for School-Age Children, Epidemiologic Version 5), with a chronic course of ADHD from childhood to adulthood (Ambrosini 2000). In addition, subjects were required to have a moderate to severe level of impairment due to symptoms of ADHD at the randomization visit, with a minimum score of 4 (moderately ill) out of 7 on the Clinical Global Impression-Severity of Illness (CGI-S) scale as well as a 25 out of 54 on the investigator-rated ADHD Rating Scale (ADHD-RS) (Adler and Cohen 2004; Barkley 1990; DuPaul 1990). In addition, subjects were required to be in good general health based on physical and laboratory examinations and medical history” | “Subjects with a current diagnosis of major depressive disorder; a current or lifetime diagnosis of bipolar or psychotic disorders; a current primary diagnosis of panic disorder, obsessive-compulsive disorder, posttraumatic stress disorder, or acute stress disorder; or who met criteria for alcohol or substance abuse within the last year were excluded. Subjects were also excluded if they were found during medical examination or interview to have an unstable medical disorder or a predisposition to seizures. In addition, subjects were queried during screening regarding previous pharmacotherapy for ADHD; those with a reported history of inadequate response to bupropion (for the treatment of ADHD) or inadequate responses to two or more adequate trials of psychostimulants were not eligible. A history of past successful treatment with bupropion or psychostimulants was not exclusionary” | Major depressive disorder; a current or lifetime diagnosis of bipolar or psychotic disorders; a current primary diagnosis of panic disorder, obsessive-compulsive disorder, posttraumatic stress disorder, or acute stress disorder | No | NR |
| Michelson 2003, p. 112 [19] (Faraone 2005) *two studies | “Adults who met DSM-IV (American Psychiatric Association 2000) criteria for ADHD as assessed by clinical interview and confirmed by the Conners’ Adult ADHD Diagnostic Interview for DSM-IV (CAAR-D; Conners et al 1999) were recruited from clinics and by advertisement. Patients were required to have at least moderate symptom severity, and the diagnosis had to be corroborated by a second reporter for either current symptoms (by a significant other) or childhood symptoms (by a parent or older sibling).” | “Patients who met diagnostic criteria for current major depression or anxiety disorder or for current or past bipolar or psychotic disorders were excluded, as were patients with serious medical illness and patients who met DSM-IV criteria for alcohol dependence. A history of episodic recreational drug use did not exclude patients, but patients actively using drugs of abuse at the time of study entry were excluded.” | Major depression or anxiety disorder or for current or past bipolar or psychotic disorders | No | 1-week medication washout and evaluation period, then 2-week placebo lead-in phase |
| Levin 2001, p. 83 [47] | “Adults ages 19-56 (25 men and 15 women) were the participants in this study. All were positive for ADHD according to Diagnostic and Statistical Manual of Mental Disorders (4th ed.; American Psychiatric Association, 1994) criteria, and all were nonsmokers verified by endtidal carbon monoxide measurements less than 8 ppm… Each participant was assessed by a trained professional for the presence of ADHD symptoms by completion of the Wender Utah Rating Scale (Wender, 1995), the Conners/Wells Adolescent and Adult SelfReport (Conners et al., 1997), a modified version of Barkley's adult ADHD semistructured interview (Barkley, Murphy, & Kwasnik, 1996), and the CGI scale (National Institute of Mental Health, 1985” | “Participants with diagnoses of major depressive disorder or generalized anxiety disorder were excluded… Medical exclusion criteria covered all relevant concerns for use of nicotine in a transdermal patch form: hypertension, cardiac disease, cerebrovascular disease, impaired renal function, history of seizure, skin disease, sensitivity to medical dressings or tapes, and history of skin allergies” | Major depressive disorder or generalized anxiety | None of the participants were on methylphenidate or other stimulant medication before the study | NR |
| Wilens 2001, p. 282 [73] | “Subjects were outpatient adults with ADHD who were between 20 and 59 years of age and who were recruited from advertisements and clinical referrals to a clinical psychopharmacology clinic” | “We excluded potential subjects if they had any clinically significant chronic medical conditions, a history of cardiac arrhythmias or seizures, mental retardation (IQ< 75), organic brain disorders, clinically unstable psychiatric conditions, bipolar disorder, drug or alcohol abuse or dependence within the 6 months preceding the study, or current use of psychotropics” | Clinically unstable psychiatric conditions, bipolar disorder | Participants with past bupropion use were excluded; NR for other medications | NR |
| Matochik 1994, p. 658 [51] | “For inclusion in the study each subject had to 1) meet the DSM-III-R criteria for ADHD, 2) meet the Utah criteria for attention deficit disorder in adulthood (5, 6), 3) have a definite childhood history of attention deficit disorder with hyperactivity, and 4) have no history of other major psychiatric disorders.” | “Any adult with a history of alcohol or substance abuse, conduct problems in childhood, or adult antisocial personality disorder was excluded.” | Adult antisocial personality disorder | No | “The subjects took no medications for at least I month before beginning the study...” |
| Weisler 2017, p. 685 [68] | “Men and non-pregnant women (aged 18–55 years) meeting the Diagnostic and Statistical Manual of Mental Disorders, Fifth Edition criteria for a primary ADHD diagnosis and having ADHD Rating Scale with Adult Prompts (ADHDRS-AP) total scores C28 at baseline were enrolled. Eligible participants had satisfactory medical assessments with no clinically significant abnormalities and they were either not currently on ADHD therapy or they reported that they were not completely satisfied with any aspect, including issues related to efficacy and tolerability, of their current ADHD therapy.” | “Participants were excluded if they had a comorbid psychiatric diagnosis that was controlled with prohibited medications or uncontrolled and associated with significant symptoms that contraindicated SHP465 MAS treatment or could confound study assessments. Participants were also ineligible if they were considered a suicide risk, had previously made a suicide attempt, or were currently demonstrating active suicidal ideation (those with intermittent passive suicidal ideation could be included based on investigator judgment). Other exclusion criteria included a body mass index (BMI) <18.5 kg/m2 (underweight) or >40 kg/m2 (very severely obese) at screening; a history of moderate-to-severe hypertension, average sitting systolic blood pressure (SBP) >139 mmHg, or average sitting diastolic blood pressure (DBP) >89 mmHg; use of combination antihypertensive medications (participants with well-controlled mild hypertension on a single antihypertensive agent could participate); a known history of symptomatic cardiovascular issues or serious cardiac problems; a known family history of sudden cardiac death or ventricular arrhythmia; a clinically significant electrocardiogram; a documented allergy, a hypersensitivity, or an intolerance to amphetamine or any excipient in SHP465 MAS; a failure to respond to an adequate course of amphetamine therapy (to ensure that response to treatment is not artificially reduced by the inclusion of non-responders); a history of suspected substance abuse or dependence disorder (excluding nicotine) based on the Diagnostic and Statistical Manual of Mental Disorders, Fifth Edition criteria; a lifetime history of amphetamine, cocaine, or other stimulant abuse and/or dependence; and participation in a clinical study within 30 days before screening.” | “...comorbid psychiatric diagnosis that was controlled with prohibited medications or uncontrolled and associated with significant symptoms that contraindicated SHP465 MAS treatment or could confound study assessments.” suicide risk, had previously made a suicide attempt, or were currently demonstrating active suicidal ideation; | No | 7–30 days washout “depending on use of prohibited medications at screening” |
| Levin 2015, p. 593 [50] (Notzon 2016, Levin 2018) | “1) were between the ages of 18-60, 2) met DSM-IV-TR criteria for current cocaine dependence, reported that cocaine was their primary drug of abuse and used cocaine at least 4 days in the prior month, 3) met DSM-IV for current adult ADHD, 4) must have a body mass index (BMI) ≥ 18 kg/m2 , 5) alcohol breathalyzer (BraC) at consent of ≤ 0.04%, and 6) capable of giving informed consent.” | “Exclusion criteria were the following: past mania,  schizophrenia, or any psychotic disorder other than transient psychosis due to drug abuse; current treatment, an unstable  psychiatric or medical condition such as uncontrolled hypertension,  or coronary vascular disease as indicated by history  or suspected by abnormal electrocardiographic results, cardiac symptoms, fainting, open-heart surgery, and/or arrhythmia; and legally mandated to substance abuse treatment” | Past mania,  schizophrenia, or any psychotic disorder other than transient psychosis due to drug abuse; “unstable” psychiatric conditions | No | 1-wk placebo  lead-in phase |
| Casas 2013, p. 268 [33](Kooij 2013) | “Eligible subjects were adults (18 – 65 years) with ADHD according to the criteria described in the Diagnostic and Statistical Manual for Mental Disorders 4th Edition Text Revision (DSM-IV-TR) (American Psychiatric Association 2000), confirmed using Conners ’Adult ADHD Diagnostic Interview Part II for DSM - IV (Conners et al. 1999). To be eligible, subjects had to score 24 on the 18 DSM-IV items measured by the investigator-rated Conners Adult ADHD Rating Scale –Screening Version (CAARS-O:SV) (Conners et al. 1999). The Structured Clinical Interview for DSM-IV Axis I Disorders was used to evaluate the presence of comorbidities and exclude other disorders (First et al. 1994); ADHD was not diagnosed if the symptoms were better accounted for by another psychiatric (e.g., mood, anxiety, psychotic or personality) disorder” | “Key exclusion criteria included known nonresponse to MPH; any clinically unstable psychiatric condition; family history of schizophrenia or affective psychosis; autism, Asperger’s syndrome, eating disorder, motor tics or history (including family history) of Tourette ’ s syndrome; substance use disorder (not including caffeine or nicotine dependence), hyperthyroidism, myocardial infarction or stroke 6 months before screening; history of seizures, glaucoma or uncontrolled hypertension; and angina pectoris or cardiac arrhythmias. Women who were pregnant or breastfeeding were also excluded.” | Any clinically unstable psychiatric condition; family history of schizophrenia or affective psychosis; autism, Asperger’s syndrome, eating disorder, motor tics or history (including family history) of Tourette’ s syndrome | No | “ADHD treatments were discontinued during the screening period” |
| Weisler 2012, p. 421 [69] | “The study included men and women (aged 18–55 years) who met the following inclusion criteria: (a) an established DSM-IV-TR diagnosis of ADHD as confirmed by the Conners Adult ADHD Diagnostic Interview for DSM-IV (CAADID);[25] (b) a Clinical Global Impression-Severity (CGI-S) score of ‡4 at screening and baseline;[26] and (c) a Conners Adult ADHD Rating Scale Self-Report: Screening Version (CAARS-S:SV) DSM-IV ADHD Total Symptoms subscale score depending on age and gender (18–39 years: ‡26 men and ‡32 women; ‡40 years: ‡29 men and ‡27 women) to ensure adequate symptom severity at baseline.” | “The main exclusion criteria were any current Axis I psychiatric condition including major depressive disorder, bipolar disorder, schizophrenia, generalized anxiety disorder, obsessive-compulsive disorder, post-traumatic stress disorder, borderline personality disorder, or eating disorder; taken any mood stabilizer, antipsychotic, antidepressant or anxiolytic within 3 months prior to screening; history of a previous suicide attempt, participants currently experiencing acute suicidal ideation or behaviour; history of alcohol or substance use disorder within 6 months prior to screening (nicotine and caffeine dependence were not exclusionary) or positive result for urine drug screen at screening or baseline; known or suspected mental retardation; and demonstrated history of nonresponse to treatment with a psychostimulant medication or to treatment with atomoxetine or methylphenidate.” | Current Axis I psychiatric condition, inc. major depressive disorder, bipolar disorder, schizophrenia, generalized anxiety disorder, obsessive-compulsive disorder, post-traumatic stress disorder, borderline personality disorder, or eating disorder; taken any mood stabilizer, antipsychotic, antidepressant or anxiolytic within 3 months prior to screening; history of suicide attempt; current acute suicidal ideation or behaviour | No | NR |
| Levin 2006, p. 137 [49] | “Study inclusion required participants to meet DSM-IV (American Psychiatric Association, 1994) criteria for opiate dependence and adult ADHD, to be between the age of 18 and 60, and on the same dose of methadone for at least 3 weeks.” | “Participants were excluded if they (1) met DSM-IV criteria for current psychiatric disorders (other than ADHD or substance abuse) which required psychiatric intervention or had a history of an eating disorder; (2) were physiologically dependent on either sedatives or alcohol, such that medical attention was required during periods of abstinence or significant reduction in amount of use; (3) exhibited suicidal or homicidal behavior within the past 2 years; (4) were taking any prescription psychotropic medications other than methadone; (5) had an unstable medical condition that would make participation hazardous (i.e., uncontrolled diabetes); (6) had a known sensitivity to MPH or BPR; (7) were nursing and/or pregnant; and (8) could not read or understand the self-report assessment forms unaided and/or were so severely impaired they could not comply with the requirements of the study, and were therefore unable to give full and informed consent.” | DSM-IV criteria for current psychiatric disorders (other than ADHD or substance abuse) which required psychiatric intervention or had a history of an eating disorder | Excluded participants taking any prescription psychotropic medications other than methadone | 2-week placebo lead-in phase |
| Kuperman 2001, p. 129 [45] | “To be given a diagnosis of ADHD, the patient met the following conditions: 1) the presence of full DSMIV criteria for a diagnosis of ADHD at the time of study entry; 2) the presence of a chronic course of ADHD symptoms from childhood to adulthood; and 3) endorsement of moderate or severe level of impairment attributed to the ADHD symptoms.” | “Patients were excluded if they had a clinically significant chronic medical condition(s), another current Axis 1 diagnosis, a history of tic disorders, mental retardation (IQ < 80), organic brain disorders, clinically unstable psychiatric symptoms (suicidal behaviors, psychosis, violence, criminality), or substance abuse within 6 months. Since bupropion SR is contraindicated in individuals with seizure disorders, any patient with a recent seizure history was excluded (14). Patients with eating disorders were excluded since they are predisposed to bupropion induced seizures (15). Patients were excluded if they were taking other psychotropic medications.” | Current Axis 1 diagnosis, a history of tic disorders, mental; clinically unstable psychiatric symptoms (suicidal behaviors, psychosis, violence, criminality) | Patients were excluded if they were taking other psychotropic medications | Included a single-blind 7-day placebo lead-in |
| Frick 2017, p. 1 [36] | “Adults (men or nonpregnant, nonlactating women aged 18-55 years) meeting Diagnostic and Statistical Manual of Mental Disorders (4th ed., text rev.; DSM-IV-TR; American Psychiatric Association, 2000) criteria for a primary ADHD diagnosis established using the Adult ADHD Clinical Diagnostic Scale Version 1.2 and having baseline ADHDRS-IV (Adler, Spencer, et al., 2009) total scores ≥32 were eligible. All eligible participants had satisfactory medical assessments, with no clinically significant or relevant abnormalities.” | “Key exclusion criteria included current comorbid psychiatric disorders (defined by the Structured Clinical Interview for the DSM-IV-TR [SCID] Axis I Disorders and controlled with prohibited medications or uncontrolled and associated with significant symptoms); any conditions/ symptoms that could confound clinical assessments at screening; chronic or acute illnesses or unstable medical conditions that could confound safety assessments, lead to increased risk, or make it difficult to comply with the protocol; a history of seizures (excluding infantile febrile seizures), any tic disorder, or a current diagnosis and/or family history of Tourette disorder; known cardiac abnormalities or conditions affecting cardiac performance, a history of hypertension, or sitting SBP >139 mmHg or DBP >89 mmHg; a clinically significant electrocardiogram (ECG) or laboratory abnormality at baseline or screening; the use of a psychoactive prescription medication or over-the-counter medication requiring more than a 28-day washout period (excluding hormonal contraceptives); participation in a clinical study within 30 days of screening; a drug dependence or substance abuse disorder (excluding SCID-defined nicotine dependence) within 6 months before screening or a positive urine drug result at screening or baseline (excluding current psychostimulant medications); and a documented allergy, intolerance, or history of nonresponse to MPH or amphetamine.” | Current comorbid psychiatric disorders | No | 7-day washout for those taking amphetamine or methylphenidate for ADHD |
| Huss 2014, p. 44 [41] (Ginsberg 2014) | “Adult patients (18–60 years) with diagnosis of ADHD, all types, with a confirmed childhood onset according to DSM-IV diagnostic criteria and a DSM-IV ADHD RS total score of C30 at screening and baseline were included in the study.” | “Exclusion criteria were: pre-existing cardiovascular or cerebrovascular disease, or any other co-morbid psychiatric disorder requiring medical intervention/therapy or that might interfere with the study conduct at the time of enrollment; patients demonstrating a >30% improvement in DSM-IV ADHD RS total score at baseline relative to that at screening were also excluded from this study. Any psychological or behavioral therapies for the treatment of ADHD were discontinued at least 1 month prior to the screening visit. Patients who initiated these therapies within 3 months prior to screening visit for reasons other than ADHD were excluded from the trial. Additionally, patients with either hypersensitivity or history of poor response or intolerance to stimulants as per the investigator’s judgment were excluded from this study” Patients with use of other investigational drugs at the time of enrollment, or within 30 days or 5 half-lives of enrollment (whichever was longer), were excluded from the study. In patients receiving any psychotropic medications the minimum discontinuation period varied according to drug class as follows: 1 week prior to the screening visit for stimulants including MPH, antidepressants other than fluoxetine, antipsychotics, anticonvulsants for nonepilepsy uses, mood stabilizing medications such as lithium, and herbal preparations with psychotropic potential; 2 weeks prior to the screening visit for benzodiazepines, barbiturates, all other sedatives or hypnotics, and monoamine oxidase inhibitors and 4 weeks prior to the screening visit for fluoxetine. . Other exclusion criteria included pregnancy, seizures, recent alcohol or drug abuse and patients with body mass index <18.5 kg/m2 or >35 kg/m2” | Any other co-morbid psychiatric disorder requiring medical intervention/therapy or that might interfere with the study conduct at the time of enrollment | No | Minimum discontinuation period varied according to drug class (see exclusions) |
| Adler 2008, p. 1364 [21] (Faraone 2012, Mattingly 2013, Babcock 2012, Kollins 2011, Ginsberg 2011, Adler 2009, Adler 2009) | “Adults aged 18-55 years with a primary diagnosis of ADHD by Diagnostic and Statistical Manual of Mental Disorders, Fourth Edition Text Revision. “All subjects were required to meet at least 6 of the 9 DSM-IV-TR subtype criteria and to have moderate to severe ADHD as rated by a clinician at baseline (ADHD-RS scores >28). Other inclusion criteria included 12-lead electrocardiogram (ECG) with QT/QTc-F interval <450 ms for men and <470 ms for women, resting heart rate 40 to 100 bpm, PR interval < 200 ms, and QRS interval < 100 ms.” | “Exclusion criteria included comorbid psychiatric diagnosis with significant symptoms that, in the judgment of the investigator, might preclude treatment with lisdexamfetamine; history of seizures; taking medications that affect the central nervous system or blood pressure (excluding current ADHD medications, which were washed out); known cardiac structural abnormality or any other condition that might affect cardiac performance; clinically significant ECG or laboratory abnormality at screening or baseline; history of hypertension, or a resting sitting systolic blood pressure (SBP) > 139 mm Hg or diastolic blood pressure (DBP) > 89 mm Hg; pregnancy or lactation; and positive urine drug results at screening or baseline (except for subject’s current stimulant therapy).” | Comorbid psychiatric diagnosis with significant symptoms that, in the judgment of the investigator, might preclude treatment with lisdexamfetamine | No | 7 to 28 day washout |
| Medori 2008 [53] (Buitelaar 2012, Rosler 2013, Buitelaar 2011) | “adult men and women with a diagnosis of ADHD according to the criteria of the Diagnostic and Statistical Manual of Mental Diseases, Fourth Edition (DSM-IV) (25) and confirmed by the Conners’ Adult ADHD Diagnostic Interview for DSM-IV (CAADID) (26). Other requirements for inclusion were age 18 to 65 years; chronic course of ADHD symptomatology from childhood to adulthood with some symptoms present before age 7 years, as determined by investigators following the CAADID interview; and CAARS total score of 24 at screening (26)” | “Patients were excluded if the investigator judged they (or their child) had a history of poor response or intolerance to methylphenidate; they had been diagnosed with any current clinically unstable psychiatric condition (e.g., acute mood disorder, bipolar disorder, acute obsessive-compulsive disorder), as determined by the investigator; or they had been diagnosed with substance use disorder (abuse/dependence) according to DSM-IV criteria within the last 6 months. Other exclusion criteria included family history of schizophrenia or affective psychosis; serious illnesses (e.g., hepatic or renal insufficiency or significant cardiac, gastrointestinal, psychiatric, or metabolic disturbances); hyperthyroidism, myocardial infarction, or stroke within 6 months of screening; and history of seizures, glaucoma, or uncontrolled hypertension” | Current clinically unstable psychiatric condition (e.g., acute mood disorder, bipolar disorder, acute obsessive-compulsive disorder); family history of schizophrenia or affective psychosis | No | “The trial included a washout period of up to 4 weeks during which current therapy was tapered to discontinuation” |
| Weisler 2006, p. 625 [67] | “Subjects were outpatients >18 years of age who were referred by clinics and had a primary diagnosis of ADHD established by psychiatric evaluation using DSM-IV-TR criteria.2 Diagnosis required identification of at least six of nine symptoms for hyperactive/impulsive and inattentive subtype criteria and ADHD onset by 7 years of age. Subjects were in good physical health, with normal vital signs and 12-lead electrocardiogram (ECG) measurements” | “Subjects incapable of following study instructions or having an intelligence quotient <80 were excluded from the study. Several comorbid psychiatric diagnoses were excluded: psychosis, bipolar illness, pervasive developmental disorder, severe obsessive-compulsive disorder, and severe depressive (17-item Hamilton Rating Scale for Depression score >19) and anxiety disorders (14-item Hamilton Rating Scale for Anxiety score >17). Subjects were excluded for a positive drug screen or substance abuse history (or living with someone with a substance abuse disorder); glaucoma; hyperthyroidism; seizure, tic disorder, or Tourette syndrome; and pregnancy or lactation. Also excluded were subjects who were taking within 30 days of the screening visit any anticonvulsant drugs, clonidine, guanfacine, systemic steroids, medications that affect blood pressure (BP) or the heart or have central nervous system effects, pemoline, or investigational drugs.” | Psychosis, bipolar illness, pervasive developmental disorder, severe obsessive-compulsive disorder, and severe depressive and anxiety disorders | No | 1 wk |
| Spencer 2007, p. 1380 [63] | “Participants eligible for inclusion were aged 18 to 60 years, diagnosed with DSM-IV ADHD (any subtype) with childhood onset of symptoms. They had to have a DSM-IV ADHD Rating Scale (ADHD-RS) total score of at least 24 at screening and baseline.” “they were required to display functional impairment, defined as a Global Assessment of Functioning (GAF) score of 60 or less.” | “Patients with a history of alcohol or substance abuse within the last 6 months were excluded, as were patients with any psychiatric or medical comorbidity that may have interfered with study participation or assessments or for which MPH treatment may have posed a risk. Patients were also excluded if the investigator judged that they had a history of poor response or intolerance to stimulants (e.g., MPH, d-MPH, amphetamine salts, or dextroamphetamine salts). No patient had previously used d-MPH-ER. Women were excluded if they were pregnant, nursing, or not using acceptable methods of contraception.” | Any psychiatric or medical comorbidity that may have interfered with study participation or assessments or for which MPH treatment may have posed a risk | No | “Patients were required to discontinue all psychotropic drugs within 1 to 4 weeks prior to the screening visit.” |
| Arnold 2014, p. 133 [26] | “Patients were included in the study if they met the full Diagnostic and Statistical Manual of Mental Disorders (4th ed., text rev.; DSM-IV-TR; American Psychiatric Association [APA], 2000) criteria for ADHD (combined type, predominantly inattentive subtype, or predominantly hyperactive-impulsive subtype), for which symptoms were present before the age of 7 years and persisted for at least the prior 6 months, according to a psychiatric/clinical evaluation using the Adult ADHD Clinical Diagnostic Scale (ACDS).” “Eligible patients were also required to have a Hamilton Anxiety Scale (HAM-A) and Hamilton Depression Scale (HAM-D; Hamilton, 1960; Maier, Buller, Philipp, & Heuser, 1988) score 24 at the screening and baseline visits, with a difference in the AISRS total score from screening to baseline < 25%. In addition, a CGI Severity of Illness (CGI-S; Guy, 1976) rating for ADHD of ≥4 at the baseline visit was required for study entry. Women of childbearing potential were required to use a medically accepted method of contraception during the study and for 30 days following their participation.” | “Exclusion criteria included a history or current diagnosis of schizophrenia, bipolar disorder, or other psychotic disorders; suicidal ideation, history of suicide attempt, or a clinical assessment of suicide risk; any acute psychiatric comorbidity (including but not limited to depression or other mood or anxiety disorder) that required pharmacotherapy, as determined by the Structured Clinical Interview for DSM-IV-TR (SCID) module assessment (Ekselius, Lindstrom, von Knorring, Bodlund, & Kullgren, 1994); a clinically significant sleep disorder; being intellectually challenged, as determined by the investigator; being satisfied with his or her current ADHD medication and having no unacceptable side effects; previous use of modafinil; use of other prescription medications for ADHD with psychoactive properties as of the baseline visit; drug or alcohol dependence within the prior 6 months; use of any antidepressant within 2 weeks before baseline; being pregnant or lactating; and presence of any clinically significant uncontrolled medical conditions.” | History or current diagnosis of schizophrenia, bipolar disorder, or other psychotic disorders; suicidal ideation, history of suicide attempt, or a clinical assessment of suicide risk; any acute psychiatric comorbidity (including but not limited to depression or other mood or anxiety disorder) that required pharmacotherapy | No | “The washout period was a minimum of 7 days after the last dose of an ADHD medication.” |
|  |  |  |  |  |  |
| Herring 2012, p. E891 [81] | “Patients meeting DSM-IV criteria for ADHD of either inattention or combined subtype and having a chronic course of behaviour disorder (initiated by age 7 years)...were enrolled” “The main inclusion criteria were age 18-55 years, a total symptom severity score on the Conners Adult ADHD Rating-Scales-Observer Screening Version (CAARS-O:SV) of >24, and a score of >4 (moderately ill) on the Clinical Global Impression-Severity of Illness scale (CGI-S).” | “The main exclusion criteria were history of other psychiatric disorders (including sleep disorders and substance abuse) or neurologic disorders and history of poor or no response to a prior course of methylphenidate or other stimulant for ADHD” | History of other psychiatric disorders (including sleep disorders and substance abuse) | No | “1-week placebo run-in period” |
| Wigal 2018, p. 111 | “Adults (aged 18 to 55 years) with a primary diagnosis of ADHD were enrolled, based on criteria outlined in the Diagnostic and Statistical Manual of Mental Disorders, Fourth Edition, Text Revision (DSM-IV-TR™).” “All subjects were also required to have scores on the ADHD-RS-IV with adult prompts ≥ 28 at baseline and a level of intellectual functioning equivalent to an intelligence quotient of ≥ 80 on the Kaufman Brief Intelligence Test” | “Key exclusion criteria were the presence of a comorbid psychiatric diagnosis with significant symptoms, a history of, or perceived risk for future suicide attempt, a recent history of substance abuse, or other medical conditions that would contraindicate treatment with psychostimulants or confound efficacy and safety assessments. Exclusion criteria also included a history of seizures; hypertension, with a resting systolic blood pressure (SBP) > 139 mm Hg or diastolic blood pressure (DBP) > 89 mm Hg; or a history of symptomatic cardiovascular disease; a structural cardiac abnormality; or a positive family history of sudden cardiac death or ventricular arrhythmia. Other exclusion criteria included adverse reactions or lack of response to previous amphetamine therapy, concomitant medications affecting the central nervous system or blood pressure (with the exception of ADHD medications that were washed out), pregnancy or lactation, a body mass index < 18.5 and ≥ 40, or a clinically significant laboratory or ECG abnormality. Subjects whose current ADHD medication provided effective control of symptoms with acceptable tolerability were also excluded” | Comorbid psychiatric diagnosis with significant symptoms, a history of, or perceived risk for future suicide attempt | No | “screening and washout (6 weeks)” |
| Bron 2014, p. 519 [80] | “We recruited drug-naive patients between 18 and 55 years of age who were diagnosed with the combined subtype of ADHD, from the PsyQ outpatient Adult ADHD clinic in The Hague, The Netherlands. ADHD diagnoses were based on having at least 6 of 9 DSM-IV symptoms of inattention and hyperactivity/ impulsivity in childhood, a chronic persisting course of symptoms and impairment, and having at least 4 of 9 DSM-IV symptoms of inattention and hyperactivity/impulsivity in adulthood” | “Exclusion criteria were: severe comorbid psychiatric disorders at time of the screening interview (using the Structured Clinical Interview for DSM disorders; SCID, First et al., 2002), treatment with stimulants, antipsychotics, clonidine, benzodiazepines, or beta-blockers within one month prior to study participation or any medication that could influence the CPT performance (i.e. TCA or SSRI), any cognitive disorder like dementia or amnesic disorder, mental retardation, or being pregnant or nursing.” | Severe comorbid psychiatric disorders at time of the screening interview | Yes | NA |
| Martin 2014, p. 147 [84] | “Individuals with a history of successful treatment with an amphetamine-based agent were recruited...” “Participants were males and nonpregnant, non-lactating females, aged 18–55 years, with a primary diagnosis of ADHD (Adult ADHD Clinical Diagnostic Scale, version 1.2) [24]. Entry requirements included a baseline score C28 on the ADHD Rating Scale IV (ADHD-RS-IV)[25] with adult prompts; a satisfactory medical assessment with no significant or relevant abnormality in medical history, physical examination, vital signs, and laboratory evaluation; a body mass index between 18.5 and 32.0 kg/m2 , inclusive; and normal or clinically insignificant screening electrocardiogram (ECG) findings, as assessed by the investigator. Female participants were postmenopausal (12 consecutive months of spontaneous amenorrhea and aged C51 years), surgically sterile, or were using or agreed to use acceptable methods of contraception and had a negative pregnancy test at screening.” | “Excluded were individuals diagnosed with a severe comorbid Axis I or Axis II disorder, based on a psychiatric evaluation that included the Mini International Neuropsychiatric Interview-Plus [26]; family history of sudden cardiac death or personal history of cardiovascular disease or structural cardiac abnormality; personal history of or current suicide risk, attempts, or ideations; documented allergy, hypersensitivity, or intolerance to amphetamines, closely related compounds, or any of the stated ingredients; history of seizures (other than infantile febrile seizures), tic disorder, or diagnosis and/or family history of Tourette disorder; and history (B12 months prior) or screening evidence of substance abuse or dependence” | Severe comorbid Axis I or Axis II disorder; history of or current suicide risk, attempts, or ideations | No | Not reported |
| Bain 2013, p. 405 [76] | “Adult male and female patients (aged 18–60 years) met the DSM-IV-TR criteria for ADHD, confirmed by the Adult ADHD Clinical Diagnostic Scale V 1.2 (Adler and Spencer, 2004) at Screening. Eligible individuals also demonstrated scores >2 (pretty much, often) on at least 6 of 9 items on the Inattentive score or the Hyperactive/Impulsive score of the Conners’ Adult Rating Scale–Investigator Rated Scale (CAARS:Inv), a total CAARS:Inv score of >20, and a Clinical Global Impression-ADHD Severity (CGI-ADHD-S) score of moderate or more impairment (>4) at Screening and Baseline.” | “Potential participants were excluded if they had any history of lifetime psychotic disorder, bipolar disorder, obsessive-compulsive disorder, or mental retardation; current generalized anxiety disorder, post-traumatic stress disorder, sleep disorder requiring treatment, or a current major depressive episode; any unstable medical condition; any condition that could affect cognitive performance; or if they were a pregnant or lactating female. Excluded psychotropic medication included anxiolytics, antipsychotics, antidepressants, mood stabilizers, nicotine replacement therapies, or varenicline. The use of atomoxetine was prohibited within 3 months before screening, and subjects receiving psychostimulants required a 7-day washout before randomization” | History of lifetime psychotic disorder, bipolar disorder, obsessive-compulsive disorder; current generalized anxiety disorder, post-traumatic stress disorder, sleep disorder requiring treatment, or a current major depressive episode | No | 7 days before randomization |
| Wender 2011, p. 36 [93] | “...adults, ages 21 to 55 years, who met the Utah Criteria for ADHD and whose symptom severity and diagnosis were assessed by the Wender-Reimherr Adult Attention Deficit Disorder Scale (WRAADDS).” | “Other Axis I and Axis II diagnoses were excluded to avoid confounding by similar symptoms arising from other psychiatric disorders. Their existence was determined via clinical interviews by the psychiatrists (PHW, FWR).” | “Other Axis I and Axis II diagnoses” | NR | 1-wk placebo run-in |
| Wigal 2010, p. 34 [96](Wigal 2011, Brams 2011) | “Adults (aged 18 to 55 years) with a primary diagnosis of ADHD were enrolled, based on criteria outlined in the Diagnostic and Statistical Manual of Mental Disorders, Fourth Edition, Text Revision (DSM-IV-TR™)... All subjects were also required to have scores on the ADHD-RS-IV with adult prompts ≥ 28 at baseline and a level of intellectual functioning equivalent to an intelligence quotient of ≥ 80 on the Kaufman Brief Intelligence Test” | “Key exclusion criteria were the presence of a comorbid psychiatric diagnosis with significant symptoms, a history of, or perceived risk for future suicide attempt, a recent history of substance abuse, or other medical conditions that would contraindicate treatment with psychostimulants or confound efficacy and safety assessments. Exclusion criteria also included a history of seizures; hypertension, with a resting systolic blood pressure (SBP) > 139 mm Hg or diastolic blood pressure (DBP) > 89 mm Hg; or a history of symptomatic cardiovascular disease; a structural cardiac abnormality; or a positive family history of sudden cardiac death or ventricular arrhythmia. Other exclusion criteria included adverse reactions or lack of response to previous amphetamine therapy, concomitant medications affecting the central nervous system or blood pressure (with the exception of ADHD medications that were washed out), pregnancy or lactation, a body mass index < 18.5 and ≥ 40, or a clinically significant laboratory or ECG abnormality” | Psychiatric diagnosis with significant symptoms, a history of, or perceived risk for future suicide attempt | No | “Except for stimulant medications and sedating antihistamines, which were discontinued 7 days prior to assessment of baseline measures, all prohibited medications were discontinued 30 days prior to screening” |
| Kay 2009, p. 316 [18] – Cohort 1 and 2 | “Eligible subjects were men or women, aged 19 to 25 years, who satisfied Diagnostic and Statistical Manual of Mental Disorders–Fourth Edition, Text Revision (DSM-IV-TR; American Psychiatric Association, 2000) criteria for a primary diagnosis of ADHD, any subtype, confirmed by a psychiatric evaluation and the Structured Clinical Interview for DSM-IV, conducted by a qualified clinician.” | “Women who were pregnant or lactating were excluded from study participation. Additional exclusion criteria included a recent history (past 6 months) of drug dependence or substance abuse (excluding nicotine); a positive urine drug screen; alcohol use 24 hours before any test day; any cardiac condition that, in the opinion of the investigator, would require exclusion; a current comorbid psychiatric diagnosis (controlled or uncontrolled) with significant symptoms that, in the opinion of the investigator, would confound efficacy or safety assessments; documented allergic or adverse reactions to MAS XR or atomoxetine; documented history of failure to respond clinically to amphetamines or atomoxetine; history of at least one seizure within the past 2 years, a tic disorder, or family history of Tourette’s syndrome; inadequately treated thyroid dysfunction; history of glaucoma; any concurrent chronic or acute illness (including severe allergic rhinitis or severe cold) that might interfere with assessments; and use of any medication that is contraindicated with MAS XR or atomoxetine or that might have confounded results of the safety assessment. In addition, subjects who were naïve to pharmacologic treatment for ADHD were excluded from study participation.” | Current comorbid psychiatric diagnosis (controlled or uncontrolled) with significant symptoms | No | “Washout period for other medications was a minimum of five half-lives and no fewer than 7 days prior to the baseline familiarization visit for stimulant medication and up to 28 days for selective serotonin reuptake inhibitors. Use of any other investigational drug within the past 30 days was prohibited.” |
| Verster 2008, p. 230 [92] (Verster 2014) | “Patients were recruited from two sources, that is referrals to outpatient clinics for adult ADHD (GGZ Delfland/PsyQ, N=10) and via an advertisement (N=8). Patients from the outpatient clinics (N= 10) underwent a standardized clinical assessment consisting of a review of prior records and a psychiatric evaluation by experienced psychiatrists using a semi-structured diagnostic interview for the presence of ADHD and co-morbid disorders both current and in childhood....Inclusion criteria were being adult (21–55 years old), having a driver’s license for at least three years, for women of childbearing potential, a negative urine pregnancy test result and the use of a medically acceptable method of contraception, normal static binocular acuity and being considered as reliable and mentally capable of adhering to the protocol.” | “Exclusion criteria included insensitivity to methylphenidate treatment, a history or presence of alcohol dependence or drug addiction, a positive alcohol breath test, the use of medication known to affect driving performance, having a psychiatric disease or excessive caffeine consumption (5 cups/day) and nicotine use (10 cigarettes/day).” | “having a psychiatric disease” | No | 3 days of no treatment |
| Barkley 2007, p. 306 [77] | “They must have received a clinical diagnosis of ADHD from a previous clinician and met Diagnostic and Statistical Manual of Mental Disorders (4th ed.; DSM-IV; American Psychiatric Association, 1994) diagnostic criteria on a structured diagnostic interview administered by a licensed clinical psychologist (D.L.A.) at the time of their screening appointment. DSM criteria had to be met both for current functioning and using retrospective reports of childhood behavior between 5 and 12 years of age” | “We excluded individuals from participation if they had any of the following conditions: met diagnostic criteria for any history of bipolar disorder or any history of a psychotic disorder (confirmed by the SCID structured interview); currently pregnant or breastfeeding; diagnosis of organic brain disease, for example, dementia, or traumatic brain injury residua; current or prior diagnosis of any seizure disorder (other than febrile seizures) or current or past use of anticonvulsant medication for seizure control; history of severe allergies to more than one class of medications or multiple adverse drug reactions; current regular use of any psychotropic medication (e.g., bupropion or a tricyclic antidepressant) on a regular basis, including health-food supplements that the investigator felt has central nervous system activity (e.g., St. John’s Wort, melatonin); current use of alcohol, drugs of abuse, or any prescribed or over-the-counter medication in a manner that the investigator considers indicative of abuse or who meet DSM-IV criteria for substance abuse; are at serious suicidal or homicidal risk in the opinion of the evaluating clinician; prior or current diagnosis of hyperthyroidism or hypothyroidism; significant prior or current medical conditions that, in the judgment of the investigator, could be exacerbated by or compromised by ATX; any current medical condition that would increase sympathetic nervous system activity markedly (e.g., catecholamine-secreting neural tumor); any current daily use of medication that has sympathomimetic activity (e.g., albuterol, inhalation aerosols, pseudoephedrine); use of monoamine oxidase inhibitors (MAOIs) during the 2 weeks (14 days) prior to Visit 2; use of any antipsychotic medication or mood stabilizers within 8 weeks of Visit 1; current or past history of hypertension (average systolic or diastolic blood pressure, measured on at least two separate occasions, greater than or equal to 140/90); current or past history of glaucoma; history of difficulty starting a stream of urine or other symptoms suggestive of prostate enlargement or other evidence of urinary hesitancy on clinical history; previous completion or withdrawal from this study or any other study investigating ATX or previous inability to tolerate ATX by history; noncompliance with taking ATX/placebo in accordance with protocol guidelines” “Any participants taking antidepressant or other forms of psychiatric medication were excluded from this project because of the prolonged washout time required prior to participants being eligible to enter into this protocol... Those on stimulant medication at the time of recruitment were instructed to obtain approval from their prescribing physicians of this requirement to be off medication for the testing session before undertaking the study procedures.” | History of bipolar disorder or any history of a psychotic disorder | No | “Participants being treated with stimulant medication at the time of study recruitment ceased taking their medication at least 48 hr prior to enrollment.” |
| Jain 2007, p. 268 [82] | “...adults 18-60 years of age with a childhood history consistent with ADHD and meeting the DSM-IV diagnosis of ADHD...Patients were eligible to participate in the study if they had a T score greater than or equal to 65 on the ADHD Index of 1 of the 2 Conners’ Adult ADHD Rating Scales-Self-rated (CAARS-S) forms completed during the baseline week and 1 of the 2 Conners’ Adult ADHD Rating Scales-Observer-rated (CAARS-O) forms completed during the baseline week; if they weighed between 50 and 90 kg at baseline assessment; if they had an IQ greater than or equal to 80 as assessed using the Wechsler Adult Intelligence Scale-III (WAIS-III) visit 1 or during the prior 5 years; and if they were able to comply with the study protocol” | “Patients were excluded from the study if they had a true allergy to methylphenidate or amphetamines; a history of serious adverse reactions to methylphenidate or were known to be methylphenidate nonresponders; serious or unstable medical illness; serious hypertension, defined as any values above 100 mm Hg diastolic and 170 mm Hg systolic; anxiety of sufficient severity to warrant treatment, based upon the Hamilton Rating Scale for Anxiety (HAM-A); depression of sufficient severity to warrant treatment, based upon the Hamilton Rating Scale for Depression (HAM-D); a history of drug or alcohol abuse; disorders of the sensory organs; autism; or psychosis or any other psychiatric conditions requiring treatment. Patients treated with the following medications were excluded from the study: guanethidine, pressor agents, monoamine oxidase inhibitors, coumarin anticoagulants, anticonvulsants, phenylbutazone, tricyclic antidepressants, selective serotonic reuptake inhibitors, or herbal remedies.” | Anxiety of sufficient severity to warrant treatment; depression of sufficient severity to warrant treatment; autism, psychosis, or any other psychiatric conditions requiring treatment | No | 1-wk washout/baseline period |
| Reimherr 2007, p. 93 [85] (Robison 2010) | “The subjects were required to have a current diagnosis of adult ADHD using the DSM-IV-TR criteria for current ADHD based on the Conners Adult ADHD Diagnostic Interview for DSM-IV with at least moderate ADHD symptoms and the Utah Criteria for ADHD in adults. Subjects were between 18 and 65 years of age. | “The following DSM-IV Axis I disorders were exclusionary: current diagnosis of major depressive disorder, generalized anxiety disorder, panic disorder, obsessive-compulsive disorder, post-traumatic stress disorder, bipolar disorder, schizophrenia, or other psychotic disorder. Subjects with a seizure disorder were also excluded. Subjects with hyperthyroidism or hypothyroidism were excluded. Finally, subjects with significant medical conditions likely to become unstable during the trial or likely to be destabilized by treatment with methylphenidate (eg cardiovascular disease) were excluded.” | Current diagnosis of major depressive disorder, generalized anxiety disorder, panic disorder, obsessive-compulsive disorder, post-traumatic stress disorder, bipolar disorder, schizophrenia, or other psychotic disorder | NR | NR |
| Kooij 2004, p. 973 [83] (Boonstra 2007, Boonstra 2005) | “Subjects were 45 out-patient adults with ADHD. They were self-referred or referred by other clinicians for assessment of ADHD to the out-patient clinic of GGZ Delfland in Delft, The Netherlands. The DSM-IV diagnosis of childhood-onset and current ADHD was determined by a psychiatrist’s clinical evaluation supplemented by the Dutch version of the DSM-IV ADHD rating scale for current symptoms (DuPaul et al. 1998). All ADHD types were eligible.” | “Subjects with co-morbid psychiatric disorders were included, unless these disorders required to be treated first or when treatment with methylphenidate was contra-indicated. We prospectively excluded subjects with clinically significant medical conditions, abnormal baseline laboratory values, a history of tic disorders, mental retardation (IQ < 75), organic brain disorders, clinically unstable psychiatric conditions (i.e. suicidal behaviours, psychosis, mania, physical aggression, currently ongoing substance abuse), current use of psychotropics, prior use of methylphenidate or amphetamines, as well as pregnant or nursing women.” | “co-morbid psychiatric disorders were included, unless these disorders required to be treated first or when treatment with methylphenidate was contraindicated”; clinically unstable psychiatric conditions (i.e. suicidal behaviours, psychosis, mania, physical aggression, currently ongoing substance abuse) | No | NR |
| Bouffard 2003, p. 546 [79] | “Subjects had to meet the following inclusion-exclusion criteria to participate in the study: DSM-IV criteria for ADHD; 1.5 or more on at least 1 ADHD self reporting questionnaire (either Conners’ Adult ADHD Rating Scale or the Adult ADHD Problem Behaviours scale); Estimated IQ of 80 or above on abbreviated WAIS-R; no psychiatric conditions that better accounted for their current symptoms or required other treatment; no medical condition contraindicating stimulants (that is, hypertension or cardiac disease) | | Psychiatric conditions that better accounted for their current symptoms or required other treatment | NR | NR |
| Tenenbaum 2002, p. 49 [91] | “Each participant met the diagnostic criteria for ADHD, Combined Type, did not meet exclusion criteria (noted below), and had a “significant other” who was willing to participate (as described in the Design and Procedure section). The diagnosis of ADHD, Combined Type was determined using criteria from the Diagnostic and statistical manual of mental disorders (4th edition; DSM-IV; American Psychiatric Association, 1994), as operationalized by clinical interview and standard rating scales.” | “Potential participants were excluded if they had any clinically significant medical conditions such as heart condition, untreated thyroid condition, or tic disorder. Participants with active substance or alcohol abuse/dependence in the six months preceding the study were also excluded. Pregnant or nursing females were excluded on the basis of self-report. Other exclusionary criteria included neurological trauma or disorder (e.g., concussion, epilepsy), chronic diseases, poor physical health, and poor vision (unless corrected). Individuals who were taking psychoactive medications (including methylphenidate) were excluded from the study unless they discontinued such medications under the supervision of their prescribing physician for the duration of the study.” | “Individuals were not excluded for the presence of psychiatric disorders unless treatment with methylphenidate was contraindicated (e.g., Panic Disorder, Major Depression - moderate or more severe) or they were clinically unstable (e.g., suicidal behavior, psychosis, criminality/violence, Bipolar Disorder).” | No | NR |
| Spencer 2001, p. 775 [86] | “...adults with ADHD between 19 and 60 years of age ascertained from clinical referrals. To be included, subjects had to satisfy full diagnostic criteria for DSM-IV ADHD based on clinical assessment confirmed by structured diagnostic interview. Attention-deficit/hyperactivity disorder diagnosis, including age of onset by 7 years, were determined by self-report as well as school records and reports by other as available.” | “We excluded potential subjects if they had any clinically significant chronic medical conditions, abnormal baseline laboratory values, IQ less than 80, delirium, dementia, or amnestic disorders, any other clinically unstable psychiatric conditions (ie bipolar disorder, psychosis), drug or alcohol abuse or dependence within the 6 months preceding the study, previous adequate trial of Adderall, or current use of psychotropics.” | Any other clinically unstable psychiatric conditions (ie bipolar disorder, psychosis) | No | No |
| Spencer 1998, p. 693 [87] | “...outpatients with ADHD who were between 19 and 60 years of age.” “To be given a diagnosis of adult ADHD, subjects had to have 1) met full DSM-III-R criteria for ADHD by age 7 as well as currently, 2) described a chronic course of ADHD symptoms, and 3) endorsed impairment associated with the disorder.” | “Exclusion criteria consisted of clinically significant chronic medical conditions, abnormal baseline laboratory values, mental retardation (IQ <75), organic brain disorders, clinically unstable psychiatric conditions, drug or alcohol abuse within the last 6 months, current use of psychotropics, and for women, pregnancy or nursing.” | Clinically unstable psychiatric conditions | NR | NR |
| Spencer 1995, p. 434 [88] | “...outpatient adults of both sexes with ADHD, between 18 and 60 years of age...the DSM-III-R diagnosis of childhood-onset and current ADHD was determined by a psychiatrist’s clinical evaluation and confirmed by a structured diagnostic interview” | “Subjects were not excluded for the presence of other psychiatric disorders unless treatment with methylphenidate was contraindicated or compliance with a double-blind protocol was judged to be jeopardized. We excluded prospective subjects if they had any clinically significant chronic medical conditions or abnormal baseline laboratory values or a history of tic disorders, mental retardation (IQ < 75), organic brain disorders, clinically unstable psychiatric conditions (ie suicidal behaviors, psychosis, delinquency, criminality, or violence) or substance or alcohol abuse or dependence within the 6 months preceding the study or currently using psychotropics. We also excluded pregnant or nursing women.” | “Subjects were not excluded for the presence of other psychiatric disorders unless treatment with methylphenidate was contraindicated or compliance with a double-blind protocol was judged to be jeopardized.” Excluded: clinically unstable psychiatric conditions (ie suicidal behaviors, psychosis, delinquency, criminality, or violence) | NR | NR |
| Wender 1985, p. 547 [94] | “...the subject must first have had a history of attention deficit disorder with hyperactivity in childhood as well as both hyperactivity and attentional deficit persisting from childhood. In addition, he or she must have had two of the following characteristics: 1) affective lability, 2) inability to complete tasks, 3) hot or explosive temper, 4) impulsivity, and 5) stress intolerance.” “additional inclusion criteria: IQs greater than 90; no contraindications to stimulant drug therapy; no history of alcohol or substance abuse in the preceding 6 months; if female, nongravid and nonlactating.” | “The exclusion criteria were that the subject had never met DSM-III criteria for schizophrenia or schizoaffective disorder, currently had no major mood disorder (including mild forms), and had none of the specific features of schizoid, schizotypal, or borderline personality disorder, such as unstable and intense interpersonal relationships with idealization and devaluation, identity disturbances, intolerance of being alone, and physically self-damaging acts, including self-mutilation and suicidal gestures.” | Schizophrenia or schizoaffective disorder, currently had no major mood disorder (including mild forms), and had none of the specific features of schizoid, schizotypal, or borderline personality disorder | NR | NR |
| Barkley 2005, p. 121 [78] | “Adults clinically diagnosed with ADHD. All participants met the following entry criteria: (a) chronological age between 18 years and 65 years; (b) composite IQ greater than 80 on the Shipley Institute of Living Test (Shipley, 1946); (c) corrected or uncorrected visual acuity of no worse than 20/30 based on a brief screening using a Snelling chart; (d) a valid state drivers license; and (e) no evidence of deafness, blindness, severe language delay, cerebral palsy, epilepsy, autism, or psychosis as established through clinical diagnostic interview and medical history” “They were required to have received an expert clinical diagnosis of ADHD established not only by meeting the DSM-IV diagnostic criteria (American Psychiatric Association, 1994) but also the judgment of an expert clinician (Dr. Murphy). The DSM criteria were amended such that the criterion for onset of symptoms was set at 12 rather than 7 years of age.” | “We excluded individuals from participation if they had any of the following conditions: (a) a history of motor or vocal tics or Tourette’s Syndrome, given some controversy over whether stimulants may create or exacerbate these conditions; (b) a history of cardiac surgery, high blood pressure (sustained blood pressure levels above the 95th percentile for age and sex) at baseline, or cerebral vascular accident, given the known cardiac presser effects of stimulant medication; (c) pregnancy; (d) a history of previous adverse reactions to stimulant medications; (e) receiving any medications that might adversely affect driving performance or might be contra-indicated with stimulants as determined by Dr. Connor; or (f) medical conditions that might affect driving performance (e.g. diabetes, retinal disease).”  “Any participants taking antidepressant or other forms of psychiatric medication were excluded from this project because of the prolonged washout time such medications typically require before they could be entered in to this protocol” | Autism, psychosis | No | “For each testing session the participants were instructed not to take any medication 24 hours prior to their testing” |
| Taylor 2001, p. 223 [90] | “...outpatient adults with ADHD” “To be given a diagnosis of adult ADHD, subjects had to meet DSM-IV criteria for the disorder from 7 years old on, with a corroborating history from at least one relative; examples of report cards, schoolwork, and prior psychologic testing; and other evidence of the disorder from childhood. To support the diagnosis, the ADHD Behavior Checklist for Adults was used... To be eligible for the study, patients had to score above the 93rd percentile of severity on both the childhood and adult versions of this scale.” | “Exclusion criteria consisted of conditions already associated with frontostriatal pathology, including organic brain disorders, schizophrenia, and Tourette disorder. ... exclude subjects with psychopathology possibly caused by neurologic insult... Medical conditions likely to affect mood or cognition, such as metabolic disorders, central nervous system conditions, mental retardation, untreated endocrine disorders, and pregnancy precluded entry into the study. Subjects using substances such as cannabis, amphetamines, cocaine, and heroin within 6 months of beginning drug trials were excluded. Subjects taking tricyclics, venlafaxine, or bupropion within 3 months, or stimulants within 2 weeks, before the beginning of the study were not included because the efficacy of these drugs for ADHD symptoms1 would make the interpretation of our results more difficult.” | Schizophrenia; medical conditions likely to affect mood or cognition | No | NR |
| Taylor 2000, p. 311 [89] | “The subjects were people more than 21 years old from a single local community... All patients underwent an assessment for ADHD, including a neurological exam; clinical, developmental, and childhood histories; and a semi-structured interview. To be given a diagnosis of adult ADHD, subjects had to (a) meet the full Diagnostic and Statistical Manual of Mental Disorders, fourth edition (DSM-IV; American Psychiatric Association 1994) criteria for the disorder by the age of 7 years as well as currently, (b) describe a chronic course of ADHD symptoms, (c) endorse at least a moderate level of impairment from the symptoms, and (d) provide corroborating history of the disorder from at least one parent or older sibling. The history included inquiry as to the nature and extent of the subject's behaviors that could be indicative of childhood ADHD. We also looked for other evidence of ADHD, such as from report cards, schoolwork, or results of prior psychological testing.” | “Exclusion criteria included narcolepsy and conditions associated with altered cognitive abilities including schizophrenia, Tourette's disorder, and diagnosable neurologic conditions... Medical conditions likely to affect mood and cognition, such as metabolic disorders, mental retardation, untreated endocrine disorders, and pregnancy, precluded entry into the study. Subjects using any cannabis, cocaine, heroin, or nonprescription amphetamines within 6 months of beginning drug trials were excluded. Subjects taking tricyclic antidepressants, venlafaxine, or bupropion within 3 months of starting the study or prescription stimulants within 2 weeks prior to the beginning of the study were not included because the efficacy of these drug s for ADHD symptoms (Wilens et al. 1995) would make the interpretation of our results more difficult” | Conditions associated with altered cognitive abilities including schizophrenia, Tourette's disorder; Medical conditions likely to affect mood and cognition | No | NR |
| Wigal 2018, p. 481 [95] | “men or nonpregnant, nonlactating women 18−55 years of age at the time of consent) meeting Diagnostic and Statistical Manual of Mental Disorders, Fourth Edition, Text Revision (DSM-IVTR) criteria for a primary ADHD diagnosis were eligible. In addition, all eligible participants had to have baseline ADHD-RS, Version IV (ADHD-RS-IV) total scores ≥24; an intelligence quotient ≥80; satisfactory medical assessments, with no clinically significant or relevant abnormalities based on medical history, physical examinations, or clinical and laboratory evaluations; and had to be able to understand and comply with study procedures.” | “A key exclusion criterion was the presence of comorbid psychiatric disorders (defined by the Structured Clinical Interview for the DSM-IV-TR [SCID] [25]), such as Axis II or Axis I disorders (e.g. posttraumatic stress disorder, psychosis, bipolar illness, severe obsessive compulsive disorder, severe depressive or anxiety disorders), that were either controlled with prohibited medications or uncontrolled with symptoms either in need of treatment or that contraindicated SHP465 MAS treatment or could confound study assessments. Other exclusion criteria included a body weight 250 lb; an unstable medical condition that could confound safety assessments, lead to increased risk, or make it difficult to comply with study procedures; a history of seizures (past 2 years); hypertension (systolic blood pressure [SBP] >140 mmHg and/or diastolic blood pressure [DBP]) >90 mmHg); antihypertensive use (excluding diuretics); a clinically significant electrocardiogram (ECG) or laboratory abnormalities; drug dependence or substance abuse disorder defined by the SCID (currently or within 12 months), excluding nicotine dependence; a positive urine drug test at screening (excluding current stimulant medication); participation in a clinical trial within 30 days of screening; a documented allergy or intolerance to amphetamines, methylphenidate, MAS IR, or extended-release MAS (MAS XR); psychoactive medication use within 30 days of screening or psychoactive over-the-counter medications requiring more than a 7-day washout (participants using methylphenidate or amphetamines at screening underwent a 7-day washout period before baseline assessments); use of atomoxetine within 30 days of screening.” | Comorbid psychiatric disorders such as Axis II or Axis I disorders (e.g. posttraumatic stress disorder, psychosis, bipolar illness, severe obsessive compulsive disorder, severe depressive or anxiety disorders), that were either controlled with prohibited medications or uncontrolled with symptoms either in need of treatment or that contraindicated SHP465 MAS treatment or could confound study assessments. | No | Psychoactive medication use within 30 days of screening or psychoactive over-the-counter medications requiring more than a 7-day washout (participants using methylphenidate or amphetamines at screening underwent a 7-day washout period before baseline assessments); use of atomoxetine within 30 days of screening. |

# **Appendix F. Risk of bias assessment**

ROB was assessed by use of Cochrane’s Risk of Bias tool for publications* that reported at least one outcome of interest.

| **Author, yr, page** | **Sequence generation** | **Allocation concealment** | **Blinding objective outcomes** | **Blinding, subjective outcomes** | **Incomplete outcome data, efficacy** | **Incomplete outcome data, safety** | **Overall assessment** |
| --- | --- | --- | --- | --- | --- | --- | --- |
| Adler 2008, p. 1364 | Unclear | Unclear | Low | Low | Low | Low | Unclear |
| Adler 2008, p. 720 | Unclear | Unclear | Low | High | High | High | High |
| Adler 2009, p. 212 | Low | Low | Low | Unclear | High | High | High |
| Adler 2009, p. 239 | Low | Low | Low | Low | High | High | High |
| Adler 2009, p. 44 | Low | Low | Low | Unclear | High | High | High |
| Adler 2013, p. 694 | Low | Low | Low | Low | High | High | High |
| Arnold 2014, p. 133 | Unclear | Unclear | Low | Low | High | High | High |
| Bain 2013, p. 405 | Unclear | Unclear | NA | Unclear | Low | Low | Unclear |
| Barkley 2005, p. 121 | Unclear | Unclear | NA | Low | Low | NA | Unclear |
| Barkley 2007, p. 306 | Unclear | Unclear | Low | Low | Unclear | Unclear | Unclear |
| Biederman2006, p. 829 | Unclear | Unclear | Low | Unclear | Unclear | Unclear | Unclear |
| Biederman 2010, p. 549 | Unclear | Unclear | Low | Unclear | Low | Unclear | Unclear |
| Biederman 2012, p. 484 | Unclear | Unclear | Low | Unclear | Low | Low | Unclear |
| Biehl 2016, p. 1 | Low | Low | Low | Unclear | High | High | High |
| Bouffard 2003, p. 546 | Low | Low | NA | Unclear | Unclear | NA | Unclear |
| Bron 2014, p. 519 | Unclear | Unclear | NA | Unclear | Low | NA | Unclear |
| Butterfield 2016, p.136 | Low | Low | NA | Low | Unclear | Unclear | Unclear |
| Casas 2013, p. 268 | Low | Low | Low | Low | High | Low | High |
| Durell 2013, p. 45 | Low | Low | Low | Low | High | High | High |
| Fan 2017 | Low | Unclear | Unclear | Unclear | Low | NA | Unclear |
| Frick 2017, p. 1 | Low | Low | Low | Low | Low | Low | Low |
| Ginsberg 2012, p. 68 | Low | Low | Low | Low | Low | Low | Low |
| Goto 2017, p. 100 | Unclear | Unclear | Low | Low | Low | Low | Unclear |
| Goodman 2017, p. 105 | Low | Low | Low | Low | Low | Low | Low |
| Hamedi 2014, p. 675 | Unclear | Low | NA | Unclear | Unclear | Unclear | Unclear |
| Herring 2012, p. e891 | Low | Low | Low | Unclear | Unclear | Unclear | Unclear |
| Huss 2014, p. 44 | Low | Low | Low | Low | Unclear | Low | Unclear |
| Jain, 2007, | Unclear | Unclear | NA | Unclear | High | High | High |
| Kay 2009, p. 316 | Unclear | Unclear | Low | Low | Unclear | Low | Unclear |
| Kollins 2014, p. 158 | Unclear | Unclear | Low | Unclear | High | Low | High |
| Konstenius 2010, p. 130 | Low | Low | NA | Unclear | Unclear | Unclear | Unclear |
| Konstenius 2014, p. 440 | Low | Low | Low | Unclear | High | High | High |
| Kooij 2004, p. 973 | Low | Unclear | NA | Low | Low | NA | Unclear |
| Kuperman 2001, p. 129 | Unclear | Unclear | NA | Unclear | Unclear | Unclear | Unclear |
| Lee 2014, p. 386 | Unclear | Unclear | Low | Unclear | High | High | High |
| Levin 2001, p. 83 | Unclear | Unclear | Low | Low | High | Unclear | High |
| Levin 2006, p. 137 | Unclear | Unclear | Low | Low | High | High | High |
| Levin 2007, p. 20 | Unclear | Unclear | Low | Unclear | High | High | High |
| Levin, 2015, p593 | Low | Low | Low | Low | High | High | High |
| Martin 2014, p. 147 | Unclear | Unclear | NA | Unclear | Low | Low | Unclear |
| Matochik 1994, p. 658 | Unclear | Unclear | NA | High | Unclear | NA | High |
| McRae-Clark 2010, p. 481 | Unclear | Low | Low | Low | High | High | High |
| Medori 2008, p.981 | Low | Low | Low | Unclear | Low | Low | Unclear |
| Michelson 2003, p. 112 | Low | Low | Low | Low | High | High | High |
| Ni 2013, p. 1959 | Low | Unclear | Low | High | High | High | High |
| Paterson 1999, p. 494 | Unclear | Unclear | Low | Unclear | Low | High | High |
| Philipsen 2015, p. 1199 | Low | Low | Unclear | Unclear | Low | Low | Unclear |
| Reimherr 2005, p. 245 | Unclear | Unclear | NA | Unclear | Unclear | Unclear | Unclear |
| Reimherr 2007, p. 93 | Unclear | Unclear | NA | Unclear | Low | Low | Unclear |
| Retz 2012, p. 48 | Low | Low | Low | Unclear | Low | Low | Unclear |
| Rosler 2009, p. 120 | Unclear | Unclear | Low | Unclear | High | High | High |
| Schrantee 2016, p. 955 | Low | Low | Low | Low | Low | Low | Low |
| Sobanski 2012, p. 100 | Unclear | Unclear | Low | High | High | High | High |
| Spencer 1995, p. 434 | Unclear | Low | Low | Low | Low | Low | Low |
| Spencer 1998, p. 693 | Unclear | Unclear | Low | Unclear | Low | Unclear | Unclear |
| Spencer 2001, p. 775 | Unclear | Low | Low | Low | Low | Unclear | Unclear |
| Spencer 2005, p. 456 | Unclear | Unclear | Low | Low | Low | Low | Unclear |
| Spencer 2007, p. 1380 | Unclear | Unclear | Low | Unclear | Low | Low | Unclear |
| Spencer 2008, p. 1437 | Unclear | Low | Low | Unclear | High | High | High |
| Sutherland 2012, p. 445 | Unclear | Unclear | Low | Unclear | High | High | High |
| Takahashi 2014, p. 488 | Low | Low | Low | Low | Low | Low | Low |
| Taylor 2000, p. 311 | Unclear | Low | NA | Unclear | Low | Unclear | Unclear |
| Taylor 2001, p. 223 | Unclear | Unclear | NA | Low | Low | NA | Unclear |
| Tenenbaum 2002, p. 49 | Unclear | Unclear | NA | Low | High | NA | High |
| Verster 2008, p. 230 | Unclear | Low | Low | NA | Low | NA | Low |
| Weisler 2006, p. 625 | Unclear | Unclear | Low | Unclear | High | High | High |
| Weisler 2012, p. 421 | Low | Low | Low | Low | Unclear | Unclear | Unclear |
| Weisler 2017, p. 685 | Low | Low | Low | Low | Unclear | Unclear | Unclear |
| Weiss 2006, p. 611 | Unclear | Unclear | Low | Low | High | High | High |
| Wender 1985, p. 547 | Unclear | Unclear | NA | Low | Low | NA | Unclear |
| Wender 2011, p. 36 | Low | Unclear | NA | Unclear | Low | Low | Unclear |
| Wigal 2010, p. 34 | Unclear | Unclear | Low | Unclear | Unclear | Unclear | Unclear |
| Wigal 2018, p. 111 | Low | Unclear | NA | Unclear | Low | NA | Unclear |
| Wigal 2018, p. 481 | Low | Unclear | NA | Low | Unclear | NA | Unclear |
| Wilens 2001, p. 282 | Unclear | Unclear | Low | Low | Low | Low | Unclear |
| Wilens 2005, p. 793 | Unclear | Unclear | Low | Low | Low | Low | Unclear |
| Wilens 2008, p. 145 | Unclear | Unclear | Low | Unclear | High | High | High |
| Winhusen 2010, p. 1680 | Low | Low | Low | Low | Unclear | Low | Unclear |
| Young 2011, p. 51 | Low | Low | Low | Unclear | High | High | High |
| * Two included publications each reported data from two unique trials; only one ROB assessment was completed for each publication. | | | | | | | |

# **Appendix G. Publication bias**

Funnel plots for all outcomes that included data reported by at least 10 unique trials

**a) Clinical response, clinician-reported- continuous (15 RCTs)**


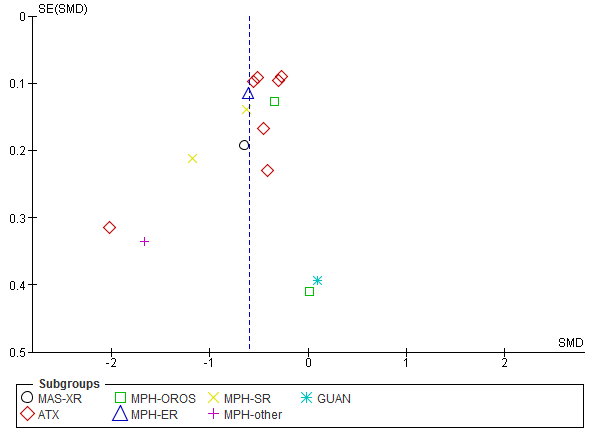


**b) Executive function (12 RCTs)**


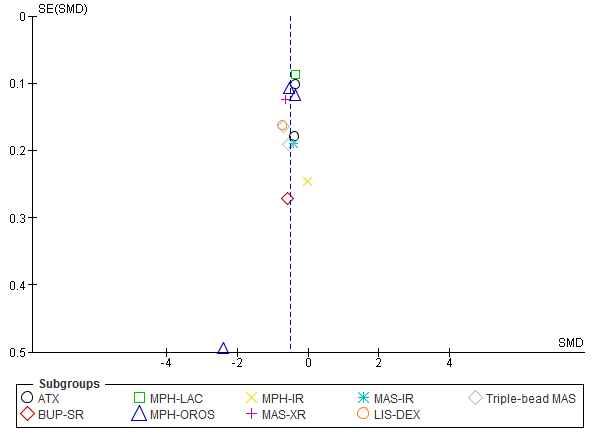


**c) Serious adverse events- any duration (33 RCTs)**


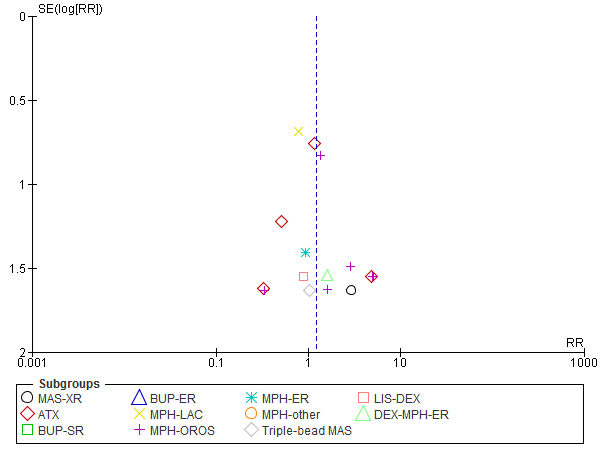


**d) Withdrawals due to adverse events- 12+ weeks (16 RCTs)**


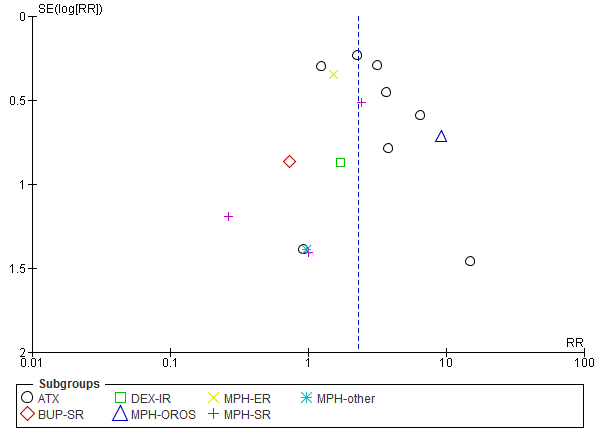


**e) Withdrawals due to adverse events- any duration (52 RCTs)**


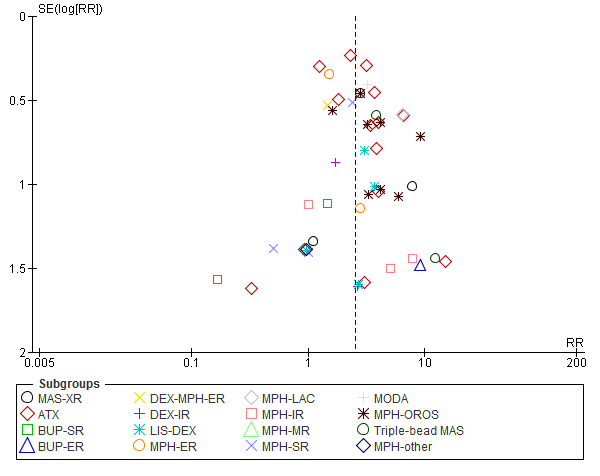


**f) Treatment discontinuation- 12+ weeks (16 RCTs)**


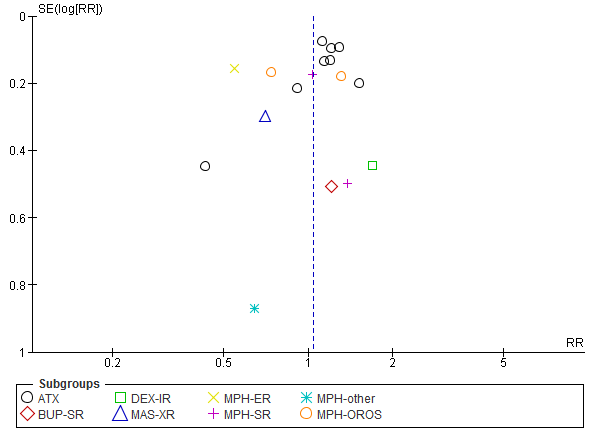


**g) Treatment discontinuation- any duration (51 RCTs)**


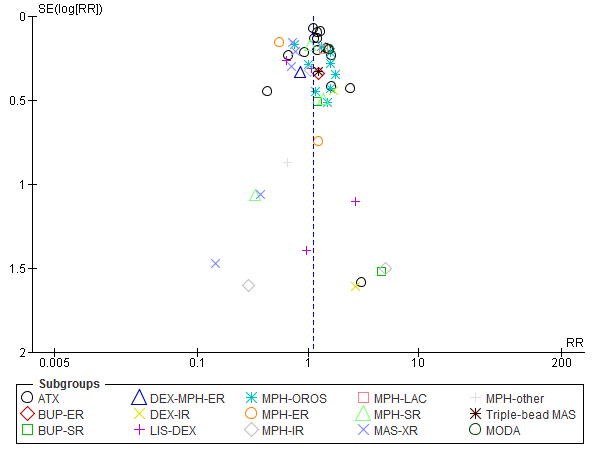


# **Appendix H. GRADE assessment**

**A. Outcomes analyzed via network meta-analysis**

**Patient-reported clinical response*: Continuous variable***

| **Comparison** | **No. of studies** | **Within-study bias** | **Reporting bias** | **Indirectness** | **Imprecision** | **Heterogeneity** | **Incoherence** | **Confidence rating** |
| --- | --- | --- | --- | --- | --- | --- | --- | --- |
| **MIXED evidence** | | | | | | | | |
| ATX-STD: Placebo | 4 | Major concerns | Undetected | Major concerns | No concerns | Some concerns | Major concerns | ⨁◯◯◯ VERY LOW |
| BUP-SR-STD: MPH-SR-HD | 1 | Major concerns | Undetected | Major concerns | Some concerns | Some concerns | Major concerns | ⨁◯◯◯ VERY LOW |
| BUP-SR-STD: Placebo | 1 | Major concerns | Undetected | Major concerns | Major concerns | No concerns | Major concerns | ⨁◯◯◯ VERY LOW |
| MPH-OROS-HD: Placebo | 1 | Major concerns | Undetected | Major concerns | Some concerns | Some concerns | Major concerns | ⨁◯◯◯ VERY LOW |
| MPH-OROS-STD: Placebo | 2 | Major concerns | Undetected | Major concerns | Some concerns | Some concerns | Major concerns | ⨁◯◯◯ VERY LOW |
| MPH-SR-HD: Placebo | 1 | Major concerns | Undetected | Major concerns | Some concerns | Some concerns | Major concerns | ⨁◯◯◯ VERY LOW |
| MPH-SR-STD: Placebo | 1 | Major concerns | Undetected | Major concerns | Some concerns | Some concerns | Major concerns | ⨁◯◯◯ VERY LOW |
| **INDIRECT evidence** | | | | | | | | |
| ATX-STD: BUP-SR-STD | 0 | Major concerns | Undetected | Major concerns | Some concerns | Some concerns | Major concerns | ⨁◯◯◯ VERY LOW |
| ATX-STD: MPH-OROS-HD | 0 | Major concerns | Undetected | Major concerns | Major concerns | No concerns | Major concerns | ⨁◯◯◯ VERY LOW |
| ATX-STD: MPH-OROS-STD | 0 | Major concerns | Undetected | Major concerns | Some concerns | Some concerns | Major concerns | ⨁◯◯◯ VERY LOW |
| ATX-STD: MPH-SR-HD | 0 | Major concerns | Undetected | Major concerns | No concerns | Some concerns | Major concerns | ⨁◯◯◯ VERY LOW |
| ATX-STD: MPH-SR-STD | 0 | Major concerns | Undetected | Major concerns | Some concerns | Some concerns | Major concerns | ⨁◯◯◯ VERY LOW |
| BUP-SR-STD: MPH-OROS-HD | 0 | Major concerns | Undetected | Major concerns | Some concerns | Some concerns | Major concerns | ⨁◯◯◯ VERY LOW |
| BUP-SR-STD: MPH-OROS-STD | 0 | Major concerns | Undetected | Major concerns | Some concerns | Some concerns | Major concerns | ⨁◯◯◯ VERY LOW |
| BUP-SR-STD: MPH-SR-STD | 0 | Major concerns | Undetected | Major concerns | Some concerns | Some concerns | Major concerns | ⨁◯◯◯ VERY LOW |
| MPH-OROS-HD: MPH-OROS-STD | 0 | Major concerns | Undetected | Major concerns | Major concerns | No concerns | Major concerns | ⨁◯◯◯ VERY LOW |
| MPH-OROS-HD: MPH-SR-HD | 0 | Major concerns | Undetected | Major concerns | Some concerns | Some concerns | Major concerns | ⨁◯◯◯ VERY LOW |
| MPH-OROS-HD: MPH-SR-STD | 0 | Major concerns | Undetected | Major concerns | Major concerns | No concerns | Major concerns | ⨁◯◯◯ VERY LOW |
| MPH-OROS-STD: MPH-SR-HD | 0 | Major concerns | Undetected | Major concerns | Some concerns | Some concerns | Major concerns | ⨁◯◯◯ VERY LOW |
| MPH-OROS-STD: MPH-SR-STD | 0 | Major concerns | Undetected | Major concerns | Major concerns | No concerns | Major concerns | ⨁◯◯◯ VERY LOW |
| MPH-SR-HD: MPH-SR-STD | 0 | Major concerns | Undetected | Major concerns | Some concerns | Some concerns | Major concerns | ⨁◯◯◯ VERY LOW |

**Patient-reported clinical response*: Dichotomous variable***

| **Comparison** | **No. of studies** | **Within-study bias** | **Reporting bias** | **Indirectness** | **Imprecision** | **Heterogeneity** | **Incoherence** | **Confidence rating** |
| --- | --- | --- | --- | --- | --- | --- | --- | --- |
| **MIXED evidence** | | | | | | | | |
| ATX-STD: Placebo | 1 | Major concerns | Undetected | Major concerns | No concerns | Major concerns | Major concerns | ⨁◯◯◯ VERY LOW |
| BUP-SR-STD: MPH-SR-HD | 1 | Major concerns | Undetected | Major concerns | Major concerns | No concerns | Major concerns | ⨁◯◯◯ VERY LOW |
| BUP-SR-STD: Placebo | 1 | Major concerns | Undetected | Major concerns | Major concerns | No concerns | Major concerns | ⨁◯◯◯ VERY LOW |
| MPH-OROS-HD: Placebo | 1 | Major concerns | Undetected | Major concerns | No concerns | Major concerns | Major concerns | ⨁◯◯◯ VERY LOW |
| MPH-SR-HD: Placebo | 1 | Major concerns | Undetected | Major concerns | Major concerns | No concerns | Major concerns | ⨁◯◯◯ VERY LOW |
| MPH-SR-STD: Placebo | 1 | Major concerns | Undetected | Major concerns | Major concerns | No concerns | Major concerns | ⨁◯◯◯ VERY LOW |
| **INDIRECT evidence** | | | | | | | | |
| ATX-STD: BUP-SR-STD | 0 | Major concerns | Undetected | Major concerns | No concerns | Major concerns | Major concerns | ⨁◯◯◯ VERY LOW |
| ATX-STD: MPH-OROS-HD | 0 | Major concerns | Undetected | Major concerns | Some concerns | Some concerns | Major concerns | ⨁◯◯◯ VERY LOW |
| ATX-STD: MPH-SR-HD | 0 | Major concerns | Undetected | Major concerns | No concerns | Major concerns | Major concerns | ⨁◯◯◯ VERY LOW |
| ATX-STD: MPH-SR-STD | 0 | Major concerns | Undetected | Major concerns | No concerns | Major concerns | Major concerns | ⨁◯◯◯ VERY LOW |
| BUP-SR-STD: MPH-OROS-HD | 0 | Major concerns | Undetected | Major concerns | Some concerns | Some concerns | Major concerns | ⨁◯◯◯ VERY LOW |
| BUP-SR-STD: MPH-SR-STD | 0 | Major concerns | Undetected | Major concerns | Major concerns | No concerns | Major concerns | ⨁◯◯◯ VERY LOW |
| MPH-OROS-HD: MPH-SR-HD | 0 | Major concerns | Undetected | Major concerns | No concerns | Major concerns | Major concerns | ⨁◯◯◯ VERY LOW |
| MPH-OROS-HD: MPH-SR-STD | 0 | Major concerns | Undetected | Major concerns | No concerns | Major concerns | Major concerns | ⨁◯◯◯ VERY LOW |
| MPH-SR-HD: MPH-SR-STD | 0 | Major concerns | Undetected | Major concerns | Major concerns | No concerns | Major concerns | ⨁◯◯◯ VERY LOW |

**Clinician-reported clinical response*: Continuous variable***

| **Comparison** | **No. of studies** | **Within-study bias** | **Reporting bias** | **Indirectness** | **Imprecision** | **Heterogeneity** | **Incoherence** | **Confidence rating** |
| --- | --- | --- | --- | --- | --- | --- | --- | --- |
| **MIXED evidence** | | | | | | | | |
| ATX-STD: Placebo | 7 | Major concerns | Undetected | Major concerns | No concerns | Some concerns | Major concerns | ⨁◯◯◯ VERY LOW |
| GUAN-STD: Placebo | 1 | Major concerns | Undetected | Major concerns | Major concerns | No concerns | Major concerns | ⨁◯◯◯ VERY LOW |
| MAS-XR-HD: Placebo | 1 | Major concerns | Undetected | Major concerns | No concerns | Some concerns | Major concerns | ⨁◯◯◯ VERY LOW |
| MPH-ER-STD: Placebo | 1 | Major concerns | Undetected | Major concerns | No concerns | Some concerns | Major concerns | ⨁◯◯◯ VERY LOW |
| MPH-LD: Placebo | 1 | Major concerns | Undetected | Major concerns | No concerns | No concerns | Major concerns | ⨁◯◯◯ VERY LOW |
| MPH-OROS-STD: Placebo | 2 | Major concerns | Undetected | Major concerns | Some concerns | Some concerns | Major concerns | ⨁◯◯◯ VERY LOW |
| MPH-SR-STD: Placebo | 2 | Major concerns | Undetected | Major concerns | No concerns | No concerns | Major concerns | ⨁◯◯◯ VERY LOW |
| **INDIRECT evidence** | | | | | | | | |
| ATX-STD: GUAN-STD | 0 | Major concerns | Undetected | Major concerns | Some concerns | Some concerns | Major concerns | ⨁◯◯◯ VERY LOW |
| ATX-STD: MAS-XR-HD | 0 | Major concerns | Undetected | Major concerns | Major concerns | No concerns | Major concerns | ⨁◯◯◯ VERY LOW |
| ATX-STD: MPH-ER-STD | 0 | Major concerns | Undetected | Major concerns | Some concerns | Some concerns | Major concerns | ⨁◯◯◯ VERY LOW |
| ATX-STD: MPH-LD | 0 | Major concerns | Undetected | Major concerns | No concerns | Some concerns | Major concerns | ⨁◯◯◯ VERY LOW |
| ATX-STD: MPH-OROS-STD | 0 | Major concerns | Undetected | Major concerns | Some concerns | Some concerns | Major concerns | ⨁◯◯◯ VERY LOW |
| ATX-STD: MPH-SR-STD | 0 | Major concerns | Undetected | Major concerns | Some concerns | No concerns | Major concerns | ⨁◯◯◯ VERY LOW |
| GUAN-STD: MAS-XR-HD | 0 | Major concerns | Undetected | Major concerns | Major concerns | No concerns | Major concerns | ⨁◯◯◯ VERY LOW |
| GUAN-STD: MPH-ER-STD | 0 | Major concerns | Undetected | Major concerns | Major concerns | No concerns | Major concerns | ⨁◯◯◯ VERY LOW |
| GUAN-STD: MPH-LD | 0 | Major concerns | Undetected | Major concerns | No concerns | Some concerns | Major concerns | ⨁◯◯◯ VERY LOW |
| GUAN-STD: MPH-OROS-STD | 0 | Major concerns | Undetected | Major concerns | Major concerns | No concerns | Major concerns | ⨁◯◯◯ VERY LOW |
| GUAN-STD: MPH-SR-STD | 0 | Major concerns | Undetected | Major concerns | Some concerns | Some concerns | Major concerns | ⨁◯◯◯ VERY LOW |
| MAS-XR-HD: MPH-ER-STD | 0 | Major concerns | Undetected | Major concerns | Major concerns | No concerns | Major concerns | ⨁◯◯◯ VERY LOW |
| MAS-XR-HD: MPH-LD | 0 | Major concerns | Undetected | Major concerns | Some concerns | No concerns | Major concerns | ⨁◯◯◯ VERY LOW |
| MAS-XR-HD: MPH-OROS-STD | 0 | Major concerns | Undetected | Major concerns | Some concerns | Some concerns | Major concerns | ⨁◯◯◯ VERY LOW |
| MAS-XR-HD: MPH-SR-STD | 0 | Major concerns | Undetected | Major concerns | Major concerns | No concerns | Major concerns | ⨁◯◯◯ VERY LOW |
| MPH-ER-STD: MPH-LD | 0 | Major concerns | Undetected | Major concerns | No concerns | Some concerns | Major concerns | ⨁◯◯◯ VERY LOW |
| MPH-ER-STD: MPH-OROS-STD | 0 | Major concerns | Undetected | Major concerns | Some concerns | Some concerns | Major concerns | ⨁◯◯◯ VERY LOW |
| MPH-ER-STD: MPH-SR-STD | 0 | Major concerns | Undetected | Major concerns | Some concerns | Some concerns | Major concerns | ⨁◯◯◯ VERY LOW |
| MPH-LD: MPH-OROS-STD | 0 | Major concerns | Undetected | Major concerns | No concerns | No concerns | Major concerns | ⨁◯◯◯ VERY LOW |
| MPH-LD: MPH-SR-STD | 0 | Major concerns | Undetected | Major concerns | Some concerns | Some concerns | Major concerns | ⨁◯◯◯ VERY LOW |
| MPH-OROS-STD: MPH-SR-STD | 0 | Major concerns | Undetected | Major concerns | Some concerns | No concerns | Major concerns | ⨁◯◯◯ VERY LOW |

**Clinician-reported clinical response: *Dichotomous variable***

| **Comparison** | **No. of studies** | **Within-study bias** | **Reporting bias** | **Indirectness** | **Imprecision** | **Heterogeneity** | **Incoherence** | **Confidence rating** |
| --- | --- | --- | --- | --- | --- | --- | --- | --- |
| **MIXED evidence** | | | | | | | | |
| ATX-STD: Placebo | 3 | Major concerns | Undetected | Major concerns | Some concerns | Some concerns | Major concerns |  |
| BUP-SR-STD: MPH-SR-HD | 1 | Major concerns | Undetected | Major concerns | Major concerns | No concerns | Major concerns |  |
| BUP-SR-STD: Placebo | 1 | Major concerns | Undetected | Major concerns | Major concerns | No concerns | Major concerns |  |
| MPH-ER-STD: Placebo | 1 | Major concerns | Undetected | Major concerns | Major concerns | No concerns | Major concerns |  |
| MPH-LD: Placebo | 1 | Major concerns | Undetected | Major concerns | No concerns | Major concerns | Major concerns |  |
| MPH-OROS-STD: Placebo | 1 | Major concerns | Undetected | Major concerns | Major concerns | No concerns | Major concerns |  |
| MPH-SR-HD: Placebo | 1 | Major concerns | Undetected | Major concerns | Major concerns | No concerns | Major concerns |  |
| MPH-SR-STD: Placebo | 1 | Major concerns | Undetected | Major concerns | Major concerns | No concerns | Major concerns |  |
| **INDIRECT evidence** | | | | | | | | |
| ATX-STD: BUP-SR-STD | 0 | Major concerns | Undetected | Major concerns | Major concerns | No concerns | Major concerns |  |
| ATX-STD: MPH-ER-STD | 0 | Major concerns | Undetected | Major concerns | Major concerns | No concerns | Major concerns |  |
| ATX-STD: MPH-LD | 0 | Major concerns | Undetected | Major concerns | No concerns | Major concerns | Major concerns |  |
| ATX-STD: MPH-OROS-STD | 0 | Major concerns | Undetected | Major concerns | Major concerns | No concerns | Major concerns |  |
| ATX-STD: MPH-SR-HD | 0 | Major concerns | Undetected | Major concerns | No concerns | Major concerns | Major concerns |  |
| ATX-STD: MPH-SR-STD | 0 | Major concerns | Undetected | Major concerns | Major concerns | No concerns | Major concerns |  |
| BUP-SR-STD: MPH-ER-STD | 0 | Major concerns | Undetected | Major concerns | Major concerns | No concerns | Major concerns |  |
| BUP-SR-STD: MPH-LD | 0 | Major concerns | Undetected | Major concerns | No concerns | Major concerns | Major concerns |  |
| BUP-SR-STD: MPH-OROS-STD | 0 | Major concerns | Undetected | Major concerns | Major concerns | No concerns | Major concerns |  |
| BUP-SR-STD: MPH-SR-STD | 0 | Major concerns | Undetected | Major concerns | Major concerns | No concerns | Major concerns |  |
| MPH-ER-STD: MPH-LD | 0 | Major concerns | Undetected | Major concerns | No concerns | Major concerns | Major concerns |  |
| MPH-ER-STD: MPH-OROS-STD | 0 | Major concerns | Undetected | Major concerns | Major concerns | No concerns | Major concerns |  |
| MPH-ER-STD: MPH-SR-HD | 0 | Major concerns | Undetected | Major concerns | Some concerns | Some concerns | Major concerns |  |
| MPH-ER-STD: MPH-SR-STD | 0 | Major concerns | Undetected | Major concerns | Major concerns | No concerns | Major concerns |  |
| MPH-LD: MPH-OROS-STD | 0 | Major concerns | Undetected | Major concerns | No concerns | Major concerns | Major concerns |  |
| MPH-LD: MPH-SR-HD | 0 | Major concerns | Undetected | Major concerns | No concerns | Major concerns | Major concerns |  |
| MPH-LD: MPH-SR-STD | 0 | Major concerns | Undetected | Major concerns | No concerns | Major concerns | Major concerns |  |
| MPH-OROS-STD: MPH-SR-HD | 0 | Major concerns | Undetected | Major concerns | Some concerns | Some concerns | Major concerns |  |
| MPH-OROS-STD: MPH-SR-STD | 0 | Major concerns | Undetected | Major concerns | Major concerns | No concerns | Major concerns |  |
| MPH-SR-HD: MPH-SR-STD | 0 | Major concerns | Undetected | Major concerns | Major concerns | No concerns | Major concerns |  |

**B. Outcomes analyzed via meta-analysis**

| **Certainty assessment** | | | | | | | **№ of patients** | | **Effect** | | **Certainty** |
| --- | --- | --- | --- | --- | --- | --- | --- | --- | --- | --- | --- |
| **№ of studies** | **Study design** | **Risk of bias** | **Inconsistency** | **Indirectness** | **Imprecision** | **Other considerations** | **CBD** | **standard care** | **Relative (95% CI)** | **Absolute (95% CI)** |  |
| **Executive function (assessed with: BRIEF-A; all durations)** | | | | | | | | | | | |
| 11 | randomised trials | serious ^a^ | not serious | serious ^b^ | not serious | none | 1870 | 1154 | - | MD 5.72 lower (7.15 lower to 4.29 lower) | ⨁⨁◯◯ LOW |
| **Quality of life (assessed with: AAQoL; > 12 weeks)** | | | | | | | | | | | |
| 5 | randomised trials | serious ^a^ | not serious | serious ^b^ | not serious | none | 992 | 870 | - | MD 4.21 higher (2.04 higher to 6.38 higher) | ⨁⨁◯◯ LOW |
| **Serious adverse events (> 12 weeks)** | | | | | | | | | | | |
| 5 | randomised trials | serious ^a^ | not serious | serious ^b^ | serious ^c^ | Publication bias strongly suspected ^d^ | 11/748 (1.5%) | 5/632 (0.8%) | RR 1.46 (0.52 to 4.09) | 4 more per 1,000 (from 4 fewer to 24 more) | ⨁◯◯◯ VERY LOW |
| **Withdrawals due to adverse events (> 12 weeks)** | | | | | | | | | | | |
| 16 | randomised trials | serious ^a^ | not serious | serious ^b^ | not serious | none ^d^ | 281/2014 (14.0%) | 91/1636 (5.6%) | RR 2.30 (1.62 to 3.25) | 72 more per 1,000 (from 34 more to 125 more) | ⨁⨁◯◯ LOW |
| **Treatment discontinuation (> 12 weeks)** | | | | | | | | | | | |
| 16 | randomised trials | serious ^a^ | serious ^e^ | serious ^b^ | serious ^c^ | none | 900/1993 (45.2%) | 661/1574 (42.0%) | RR 1.04 (0.91 to 1.20) | 17 more per 1,000 (from 38 fewer to 84 more) | ⨁◯◯◯ VERY LOW |
| Note: **CI:** Confidence interval; **MD:** Mean difference; **RR:** Risk ratio a. Most studies were at high or unclear risk of bias. Risk of unblinding due to adverse events is a concern despite double-blind designs.  b. RCTs exclude patients with psychiatric comorbidities, including anxiety and depression  c. Confidence interval includes both benefits and harms  d. Few studies report on long-term outcomes  e. High heterogeneity | | | | | | | | | | | |

# **Appendix I. Clinical response**

**i) Patient-reported clinical response in trials with a treatment duration of at least 12 weeks – Indirect comparison of ADHD pharmacotherapies**

|  | **Standardized mean difference (95% credible interval)*** | | | | | | |
| --- | --- | --- | --- | --- | --- | --- | --- |
|  | **Placebo** | **ATX-STD** | **BUP-SR-STD** | **MPH-OROS-STD** | **MPH-OROS-HD** | **MPH-SR-STD** | **MPH-SR- HD** |
| **Placebo** | — |  |  |  |  |  |  |
| **ATX-STD** | **-0.51  (-1.07, -0.03)** | — |  |  |  |  |  |
| **BUP-SR-STD** | -0.07  (-1.15, 0.97) | 0.44  (-0.71, 1.64) | — |  |  |  |  |
| **MPH-OROS-STD** | -0.40  (-1.20, 0.32) | 0.11  (-0.82, 1.02) | -0.34  (-1.65, 0.96) | — |  |  |  |
| **MPH-OROS-HD** | -0.54  (-1.64, 0.55) | -0.03  (-1.20, 1.22) | -0.47  (-2.00, 1.06) | -0.14  (-1.45, 1.21) | — |  |  |
| **MPH-SR-STD** | -0.41  (-1.41, 0.61) | 0.10  (-0.98, 1.29) | -0.34  (-1.76, 1.15) | 0.00  (-1.23, 1.30) | 0.13  (-1.31, 1.65) | — |  |
| **MPH-SR-HD** | 0.28  (-0.79, 1.33) | 0.79  (-0.34, 2.01) | 0.35  (-0.69, 1.42) | 0.69  (-0.56, 2.02) | 0.82  (-0.72, 2.34) | 0.69  (-0.79, 2.14) | — |
| Note: ATX = atomoxetine, BUP-SR = sustained release bupropion, HD = high dose, MPH = methylphenidate, OROS = osmotic-release oral system, STD = standard dose, SR = sustained release.  *Random-effects model. A negative SMD indicates improvement in clinical response. Statistically significant changes are indicated by use of bold and colour (green indicates that the row treatment is significantly better than the column treatment). White indicates no significant difference between treatments. | | | | | | | |

**ii) Clinician-reported clinical response in trials with a treatment duration of at least 12 weeks – Indirect comparison of ADHD pharmacotherapies**

|  | **Standardized mean difference (95% credible interval)*** | | | | | | |
| --- | --- | --- | --- | --- | --- | --- | --- |
|  | **Placebo** | **MAS-XR-HD** | **ATX-STD** | **GUAN-STD** | **MPH-OROS-STD** | **MPH-ER-STD** | **MPH-SR-STD** |
| **Placebo** | — |  |  |  |  |  |  |
| **MAS-XR-HD** | -0.65 (-1.89, 0.55) | — |  |  |  |  |  |
| **ATX-STD** | **-0.57 (-1.05, -0.13)** | 0.09 (-1.22, 1.37) | — |  |  |  |  |
| **GUAN-STD** | -0.09 (-1.46, 1.29) | 0.57 (-1.28, 2.41) | 0.48 (-0.95, 1.97) | — |  |  |  |
| **MPH-OROS-STD** | -0.21 (-1.09, 0.69) | 0.44 (-1.05, 1.95) | 0.36 (-0.61, 1.39) | -0.12 (-1.77, 1.52) | — |  |  |
| **MPH-ER-STD** | -0.61 (-1.79, 0.58) | 0.05 (-1.65, 1.75) | -0.04 (-1.29, 1.25) | -0.52 (-2.34, 1.30) | -0.40 (-1.90, 1.07) | — |  |
| **MPH-SR-STD** | **-0.89 (-1.75, -0.04)** | -0.23 (-1.73, 1.25) | -0.32 (-1.27, 0.66) | -0.80 (-2.41, 0.80) | -0.67 (-1.93, 0.55) | -0.28 (1.73, 1.16) | — |
| **MPH-LD** | **-1.62**  **(-2.96, -0.33)** | -0.97  (-2.74, 0.80) | -1.05  (-2.44, 0.34) | -1.53  (-3.45, 0.37) | -1.41  (-3.03, 0.15) | -1.01  (-2.77, 0.74) | -0.74  (-2.31, 0.82) |
| Note: ATX = atomoxetine, GUAN = guanfacine, HD = high dose, MAS-XR =mixed amphetamine salts, MPH =methylphenidate, OROS = osmotic-release oral system, ER = extended release, SR = sustained release, STD = standard dose, CrI = credible interval, SMD = standardized mean difference. *Random-effects model. A negative SMD indicates improvement in clinical response. Statistically significant changes are indicated by use of bold and colour (green indicates that the row treatment is significantly better than the column treatment). White indicates no significant difference between treatments. | | | | | | | |

# **Appendix J. Executive function**

Direct comparison (meta-analysis) of ADHD pharmacotherapies v. placebo among RCTs with any treatment duration. Negative SMD indicates improvement. Data in the main text are expressed on the BRIEF-A scale.

**
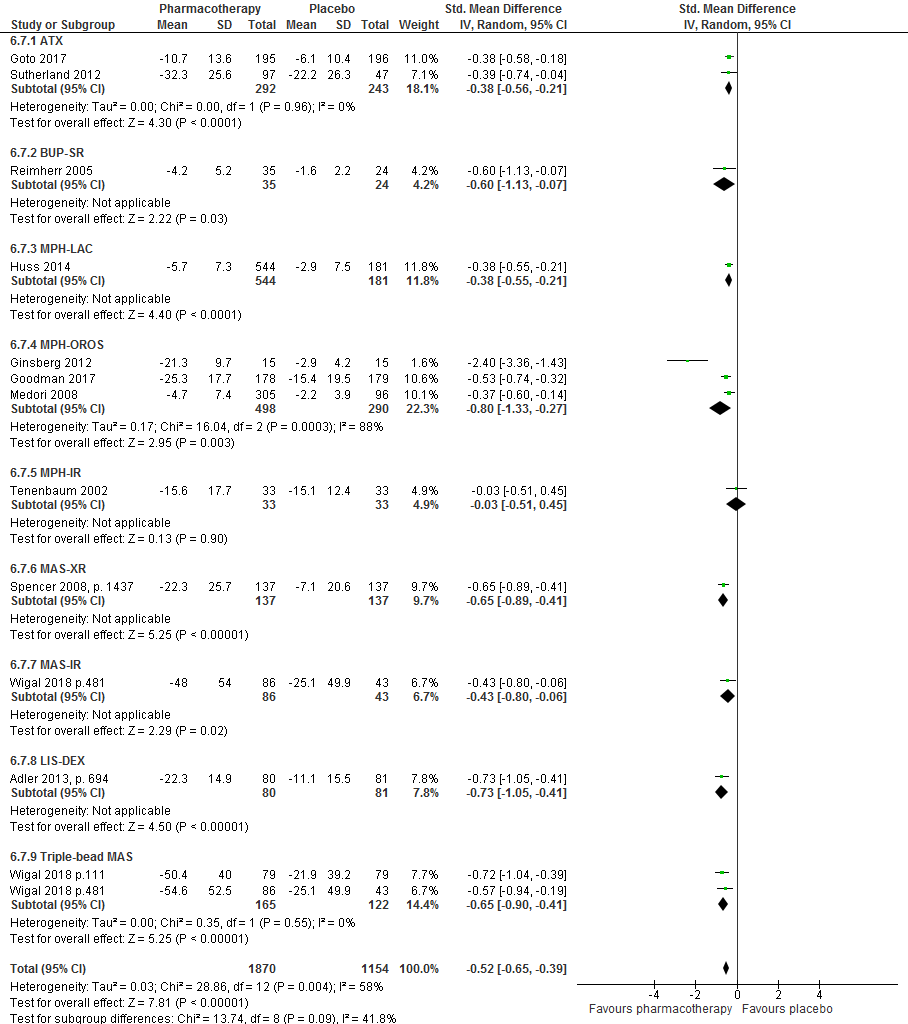
**

# **Appendix K. Quality of life**

Direct comparison (meta-analysis) of atomoxetine v. placebo among RCTs with a treatment duration of at least 12 weeks. Among the included RCTs, quality of life was assessed only for atomoxetine; no data available for other pharmacotherapies. Positive SMD indicates improvement. Data in the main text are expressed on the Adult ADHD Quality of Life Scale.

# **Appendix L. Driving behavior**

| **Author, year** | **Scale** | **Treatment duration** | **Comparison** | **Finding** |
| --- | --- | --- | --- | --- |
| **Durell**  **2013** | Driving Behavior Survey Self-Report (self, observer) | 12 wk | Placebo v. ATX (standard dose) | “After 12 weeks of treatment, there was no statistically significant difference between atomoxetine-treated and placebo-treated participants on the … DBS-Self, the DBS-Other, …” |
| **Sobanski 2012** | German Driver Coping Questionnaire (self) | 12 wk | No treatment  v. ATX (standard dose) | “In both groups coping strategies for driving-related stress did not change from baseline to endpoint” |
| **Biederman 2012** | Manchester Driving Behavior Questionnaire* (self) | 6 wk | Placebo v. LDX (high dose) | “LDX treatment was associated with significant improvements in self-reported driving behaviors that were independent of improvement in symptoms of ADHD. These results suggest that LDX may reduce behaviors associated with driving risks in young adults with ADHD.” |
| **Adler**  **2008** | Driving Behavior Survey (self, observer) | 6 mo | Placebo v. ATX (standard dose) | “At 6 months, both the atomoxetine and placebo groups reported better driving behaviors by self-report; however, there were no statistically significant differences between treatment groups using the self-reported total score. In the subsample where observer ratings were available, patients in the atomoxetine group (n = 156) were rated as significantly more improved in driving than those in the placebo group (n = 96)…” |
| **Barkley 2007** | Driving Anger Scale (self); Safe Driving Behaviour Rating Scale (self, observer) | 4 wk cross-over | Placebo v. ATX (standard dose) | Significant beneficial effects [of atomoxetine] were evident on … self-ratings of safe driving behavior... No beneficial effects were evident, however, on ratings of others on these same parameters…” No significant differences were observed on the Driving Anger Scale (self). |
| ATX = atomoxetine, DBS = Driving Behaviour Scale, DBQ = Driving Behavior Questionnaire, LDX = lisdexamfetamine. *US version | | | | |

# **Appendix M. Serious adverse events**

Direct comparison (meta-analysis) of ADHD pharmacotherapies v. placebo among RCTs with (a) treatment duration of at least 12 weeks or (b) any treatment duration.

**A) Duration of at least 12 weeks**

**
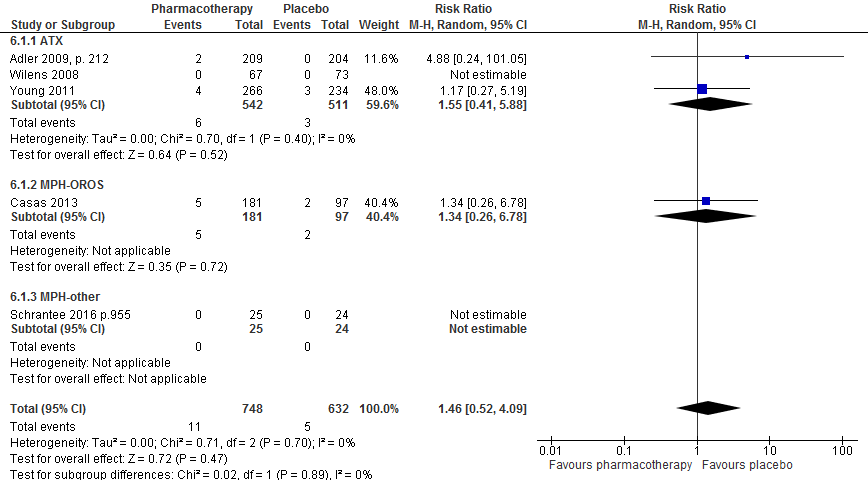
**

**B) Any treatment duration**

**
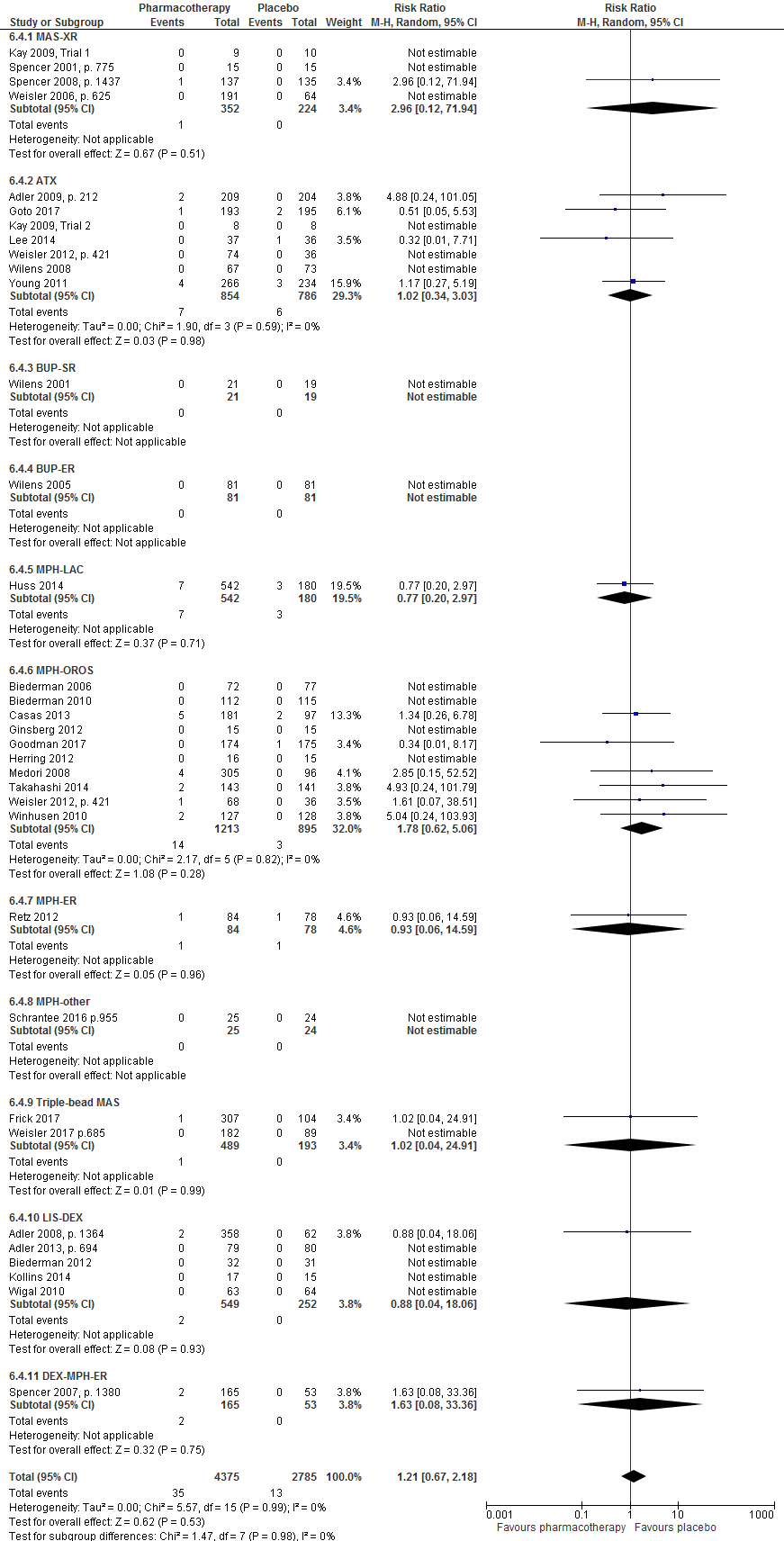
**

# **Appendix N. Withdrawals due to adverse events**

Direct comparison (meta-analysis) of ADHD pharmacotherapies v. placebo among RCTs with (a) treatment duration of at least 12 weeks or (b) any treatment duration.

**A) Duration at least 12 weeks**

**
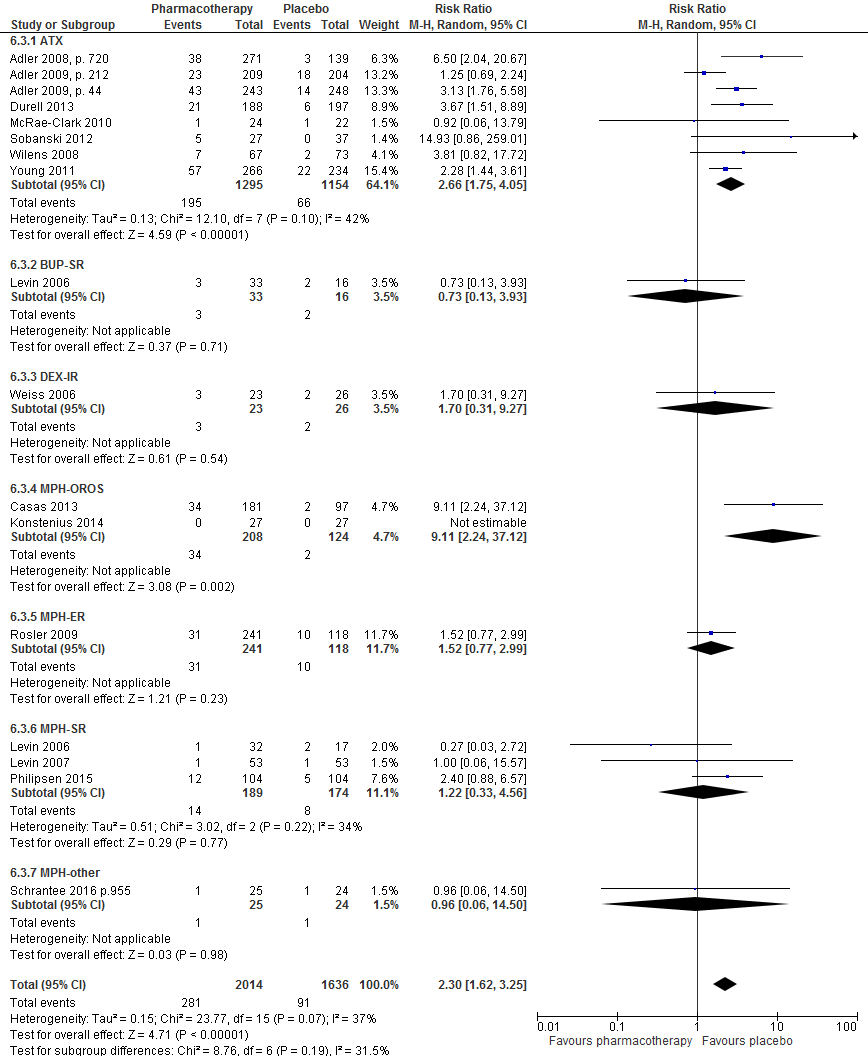
**

**B) Any treatment duration**

**
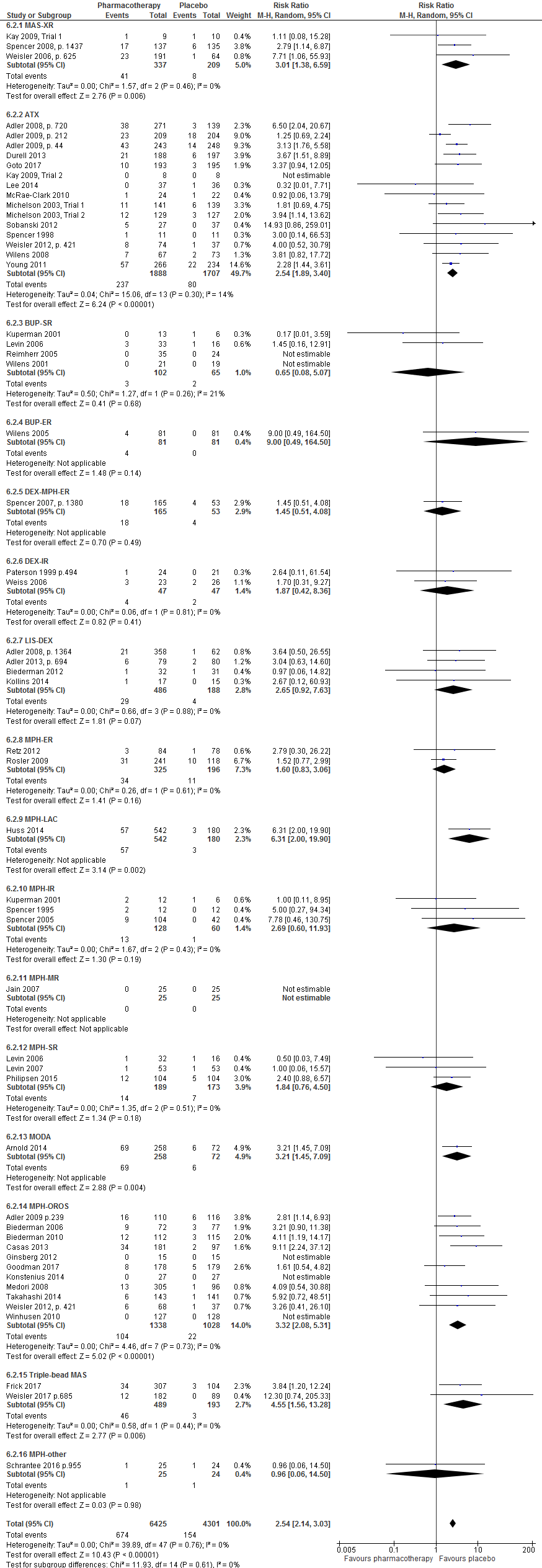
**

# **Appendix O. Treatment discontinuation**

Direct comparison (meta-analysis) of ADHD pharmacotherapies v. placebo among RCTs with (a) treatment duration of at least 12 weeks or (b) any treatment duration.

**A) Duration at least 12 weeks**

**
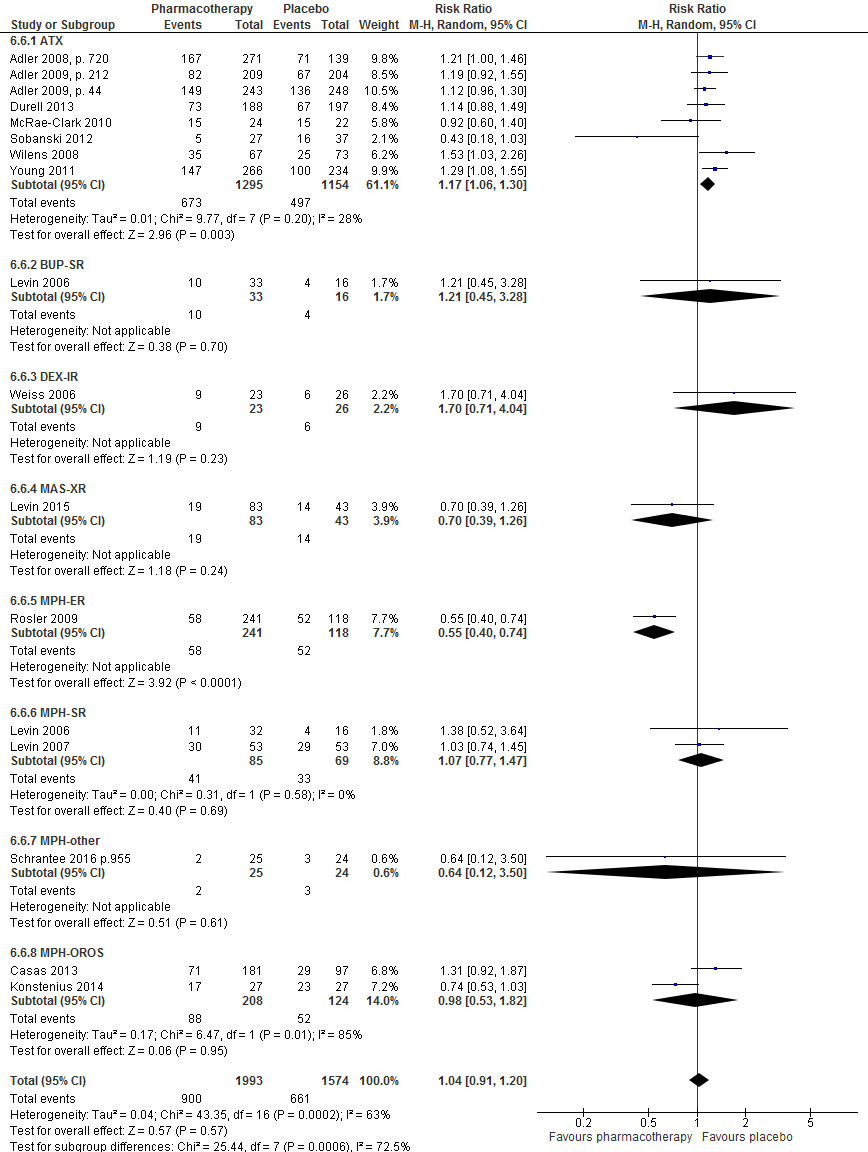
**

**B) Any treatment duration**


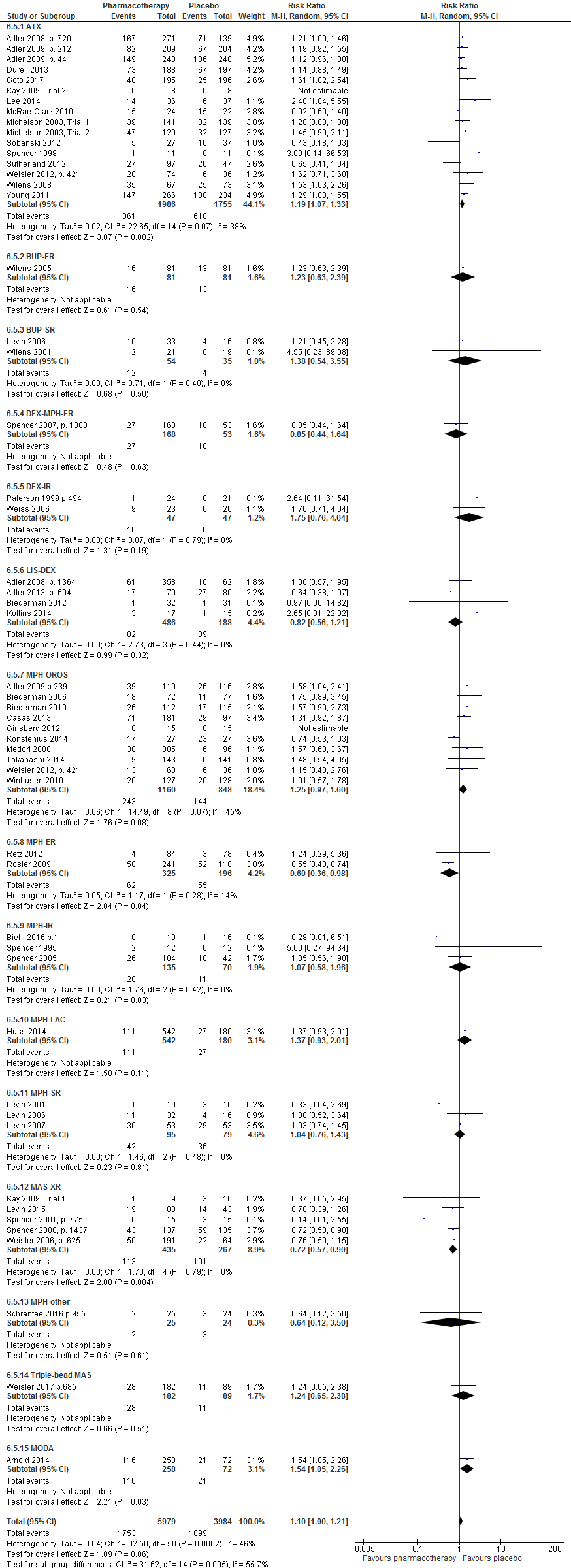


# **Appendix P. Sensitivity analyses**

**A) Patient-reported clinical response (continuous scale) — Low ROB for blinding only**

**MA findings**

**NMA findings**

|  | **Standardized mean difference (95% credible interval)*** | | | | |
| --- | --- | --- | --- | --- | --- |
|  | **Placebo** | **ATX-STD** | **BUP-SR-STD** | **MPH-OROS-STD** | **MPH-SR- HD** |
| **Placebo** | — |  |  |  |  |
| **ATX-STD** | -0.35  (-6.59, 5.69) | — |  |  |  |
| **BUP-SR-STD** | -0.05 (-6.34, 6.31) | 0.29 (-8.37, 9.10) | — |  |  |
| **MPH-OROS-STD** | -0.33 (-6.62, 5.90) | 0.01 (-8.82, 8.83) | -0.28  (-9.16, 8.52) | — |  |
| **MPH-SR-HD** | 0.30  (-5.98, 6.72) | 0.65 (-8.00, 9.40) | 0.36  (-5.90, 6.65) | 0.63 (-8.22, 9.56) | — |
| Note: ATX = atomoxetine, BUP = bupropion, HD = high dose, MPH = methylphenidate, OROS = osmotic-release oral system, STD = standard dose, SR = sustained release.  *Random-effects model. | | | | | |

**B) Patient-reported clinical response (dichotomous) — Low ROB for blinding only**

**MA findings**


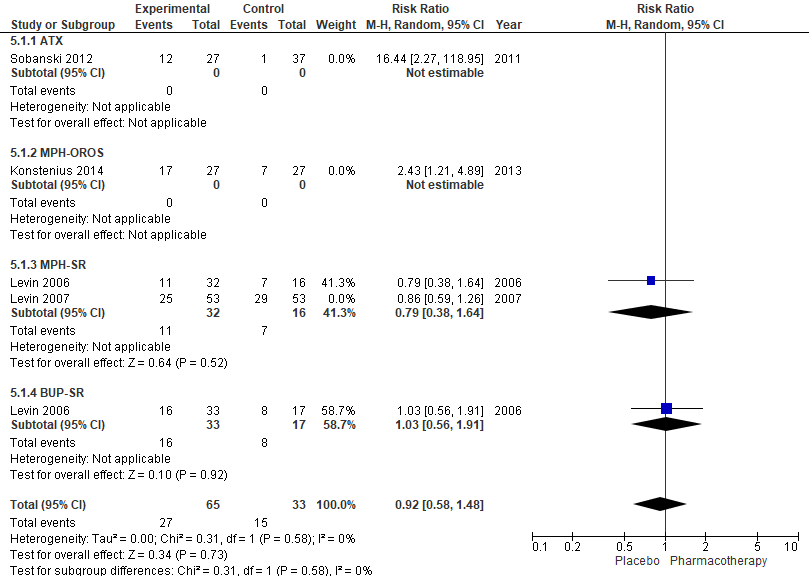


**NMA findings**

|  | **SMD (95% CrI)*** | | | | | |
| --- | --- | --- | --- | --- | --- | --- |
|  | **Placebo** | **MAS-XR-HD** | **ATX-STD** | **GUAN-STD** | **MPH-OROS-STD** | **MPH-LD** |
| **Placebo** | — |  |  |  |  |  |
| **MAS-XR-HD** | -0.66  (-4.55, 3.37) | — |  |  |  |  |
| **ATX-STD** | -0.38  (-3.16, 2.52) | 0.28  (-4.58, 5.26) | — |  |  |  |
| **GUAN-STD** | -0.11  (-4.29, 3.86) | 0.55  (-5.29, 6.14) | 0.27  (-4.80, 5.07) | — |  |  |
| **MPH-OROS-STD** | -0.35  (-4.42, 3.67) | 0.31  (-5.37, 5.91) | 0.03  (-4.99, 4.92) | -0.24  (-5.88, 5.52) | — |  |
| **MPH-LD** | -1.63  (-5.70, 2.42) | -0.97  (-6.69, 4.67) | -1.26  (-6.19, 3.62) | -1.52  (-7.22, 4.31) | -1.28  (-6.98, 4.47) | — |
| Note: ATX = atomoxetine, GUAN = guanfacine, HD = high dose, MAS-XR =mixed amphetamine salts, MPH = methylphenidate, OROS = osmotic-release oral system, ER = extended release, SR = sustained release, STD = standard dose, CrI = credible interval, SMD = standardized mean difference. *Random-effects model. | | | | | | |

**C) Clinician-reported clinical response (dichotomous) — Low ROB for blinding only**

**MA findings**

**NMA findings**

|  | **Relative risk (95% Crl)*** | | | | | |
| --- | --- | --- | --- | --- | --- | --- |
|  | **Placebo** | **ATX-STD** | **BUP-SR-STD** | **MPH-OROS-STD** | **MPH-SR-HD** | **MPH-LD** |
| **Placebo** | — |  |  |  |  |  |
| **ATX-STD** | 1.51  (0.74, 2.44) | — |  |  |  |  |
| **BUP-SR-STD** | 0.73  (0.22, 1.86) | 0.49  (0.14, 1.52) | — |  |  |  |
| **MPH-OROS-STD** | 1.48 (0.74, 2.45) | 0.98 (0.44, 2.27) | 2.01 (0.65, 6.98) | — |  |  |
| **MPH-SR-HD** | 0.42 (0.10, 1.26) | 0.28 (0.06, 1.01) | 0.58 (0.16, 1.89) | 0.29 (0.06, 1.03) | — |  |
| **MPH-LD** | **3.14 (1.87, 5.02)** | **2.09 (1.12, 4.65)** | **4.33 (1.52, 14.84)** | **2.12 (1.11, 4.70)** | **7.51 (2.22, 34.11)** | — |
| Note: ATX = atomoxetine, CrI = credible interval, , ER = extended release, HD = high dose, LD = low dose, MPH = methylphenidate, OROS = osmotic-release oral system, SR = sustained release, STD = standard dose. *Random-effects model. | | | | | | |

**D) Patient-reported clinical response (dichotomous scale) — Sobanski et al (2012) removed**

**E) Clinician-reported clinical response (continuous scale) — Sobanski et al (2012) removed**
